# Reaction of bis[(2-chlorocarbonyl)phenyl] Diselenide with Phenols, Aminophenols, and Other Amines towards Diphenyl Diselenides with Antimicrobial and Antiviral Properties †

**DOI:** 10.3390/molecules22060974

**Published:** 2017-06-12

**Authors:** Mirosław Giurg, Anna Gołąb, Jakub Suchodolski, Rafał Kaleta, Anna Krasowska, Egbert Piasecki, Magdalena Piętka-Ottlik

**Affiliations:** 1 Department of Organic Chemistry, Faculty of Chemistry, Wrocław University of Science and Technology, Wybrzeże Wyspiańskiego 27, 50-370 Wrocław, Poland; anna_golab@interia.pl (A.G.); rafal.kaleta@op.pl (R.K.); 2 Department of Biotransformation, Faculty of Biotechnology, University of Wrocław, Joliot-Curie 14a, 50-383 Wrocław, Poland; jakub.suchodolski@uwr.edu.pl (J.S.); anna.krasowska@uwr.edu.pl (A.K.); 3 Hirszfeld Institute of Immunology and Experimental Therapy, Polish Academy of Sciences, Weigla 12, 53-114 Wrocław, Poland; piasecki@iitd.pan.wroc.pl; 4 Department of Organic and Pharmaceutical Technology, Faculty of Chemistry, Wrocław University of Science and Technology, Wybrzeże Wyspiańskiego 27, 50-370 Wrocław, Poland

**Keywords:** ebselen, organoselenium compounds, diselenides, benzisoselenazol-3(2*H*)-ones, antimicrobial activity, antiviral activity, morpholine, benzohydrazide, 2-hydroxyacetanilide, antimycin A

## Abstract

A reaction of bis[(2-chlorocarbonyl)phenyl] diselenide with various mono and bisnucleophiles such as aminophenols, phenols, and amines have been studied as a convenient general route to a series of new antimicrobial and antiviral diphenyl diselenides. The compounds, particularly bis[2-(hydroxyphenylcarbamoyl)]phenyl diselenides and reference benzisoselenazol-3(2*H*)-ones, exhibited high antimicrobial activity against Gram-positive bacterial species (*Enterococcus* spp., *Staphylococcus* spp.), and some compounds were also active against Gram-negative *E. coli* and fungi (*Candida* spp., *A. niger*). The majority of compounds demonstrated high activity against human herpes virus type 1 (HHV-1) and moderate activity against encephalomyocarditis virus (EMCV), while they were generally inactive against vesicular stomatitis virus (VSV).

## 1. Introduction

Organoselenium chemistry and biology have been areas of continuous research over the last few decades [1,2,3,4,5,6,7,8,9,10,11,12,13,14,15,16,17]. Selenium-containing molecules have been of interest as reagents in new synthetic routes [1], promising catalysts of various oxidation reactions [1,2,3,4,5,6,7,18,19,20,21], and bioactive agents [8,9,10,11,12,13,14,15,16,22,23]. In the context of biological applications, organoselenium compounds have received particular attention as glutathione peroxidase (GPx) mimics [9,11,14,24], antioxidant, and anti-inflammatory agents [10,11,13,23]. Furthermore, a number of selenium-containing compounds have been studied as anticancer [12,22,25], anti-angiogenic [26], antiviral [27,28,29,30,31,32,33,34,35], antimicrobial [32,33,34,35,36,37,38,39,40,41,42,43,44,45,46], and antileishmanial agents [47].

The development of novel anti-infective agents is an area of intense activity due to the constant appearance of new infections, the emergence of multidrug resistance in common pathogens, as well as the need to eliminate undesirable side effects of available drugs. The urgent need for the next generation of antimicrobials and antivirals perpetuates research into designing novel molecules and investigating their mechanisms of action. The tremendous efforts in this area has led to the identification of many bioactive structures [27,28,29,30,31,32,33,34,35,36,37,38,39,40,41,42,43,44,45,46]. Several enzymes, among them thioredoxin reductase [36,48], bacterial urease [49], and plasma membrane H+-ATPase [50,51,52], were suggested as targets for organoselenium compounds with antimicrobial properties. Furthermore, it has been reported that the antiviral effects of organoselenium compounds might be related to the inhibition of hepatitis C virus NS3 helicase [28], HIV-1 capsid dimerization [29], and nucleocapsid protein 7 [30].

Inspired by the encouraging findings in the area of antimicrobial and antiviral properties of organoselenium compounds and as a continuation of our interest in the chemistry and biology of this class of compounds, we aimed to develop a series of new diphenyl diselenides and investigate their activity against various microbial strains and viruses, along with selected cyclic analogues. It seemed interesting to focus our attention on a group of 2,2’-dicarbamoylphenyl diselenides **4** as the most reduced analogues of ebselen, with a phenyl ring containing hydroxy and/or methoxy moieties. However, the rationale behind studying these compounds was based not only on the fact that the structural modifications in the diphenyl diselenide scaffold seemed to be interesting from the medicinal chemistry perspective; we also aimed to investigated a synthetic route to reduced forms of ebselenols (2-(hydroxyphenyl)-1,2-benzisoselenazol-3(2*H*)-ones) and its derivatives via the reaction of bis[(2-chlorocarbonyl)phenyl] diselenide **3** with various mono and bisnucleophiles such as aminophenols, phenols, and amines.

Up to the present time, several methods have been applied for the preparation of bis(2-arylcarbamoyl)phenyl diselenides **4**. One path is the reductive benzisoselenazolone ring opening of ebselenols and its derivatives [53], which can be obtained from 2-(chloroseleno)benzoyl chloride with moderate to excellent yields [54,55,56]. An alternative route to ebselenols requires metalation-selenenylation of appropriate benzanilides while protecting the hydroxyl group in the first step [57,58]. Although the reductive ring opening reaction has been successfully applied for the reduction of ebselen and its various alkyl analogues [59], in the case of ebselenols, yields were less satisfactory [53]. Only bis[2-(4-hydroxyphenylcarbamoyl)]phenyl diselenide was prepared in 46% yield while bis[2-(2-hydroxyphenylcarbamoyl)]phenyl diselenide and 2-(3-hydroxyphenylcarbamoyl)]phenyl diselenide were obtained in very low yields of 8.3% and 12%, respectively [53]. Another, general route to ebselenol-derived diselenides which we present in this study is the reaction of bis[(2-chlorocarbonyl)phenyl)] diselenide **3** with the corresponding anilines. In the past, this reaction was successfully applied to obtain various 2,2’-dicarbamoyldiphenyl diselenides [60]. However, to our best knowledge, there is only one example of a reaction with an electron-rich arene system bearing a hydroxyl group [26] but with no synthetic details. Thus, we aimed to investigate the behaviour of bis[(2-chlorocarbonyl)phenyl)] diselenide **3** in the reaction with *N* and *O* nucleophiles, such as aminophenols, phenols, and amines. The aminophenols were of particular interest as the ones containing two nucleophilic centers in the molecule, with both amine nitrogen and hydroxide oxygen potentially susceptible toward acylation. The reaction of various *O* and *N* nucleophiles with another selenium-containing chloride, 2-(chloroseleno)benzoyl chloride **9**, showed the preference of a primary amino group toward selenenylation-acylation [55]. As chloride **9** contains both hard and soft electrophilic centers localized on the carbonyl carbon and selenium atoms, respectively, the results of the reaction with *O*-nucleophiles strongly depended on the nucleophile structure. However, in the absence of the soft electrophilic center, no side reactions should occur. Thus, we expected that the reaction of bis[(2-chlorocarbonyl)phenyl] diselenide **3** with aminophenols could be considered as a convenient alternative for low-efficient reductive ring opening of ebselenols [53] and the preparation from 2,2’-dicarboxydiphenyl diselenide using active ester coupling agents [23,61], where the protection of the hydroxyl group would be recommended.

## 2. Results and Discussion

### 2.1. Synthesis

**Scheme 1:**
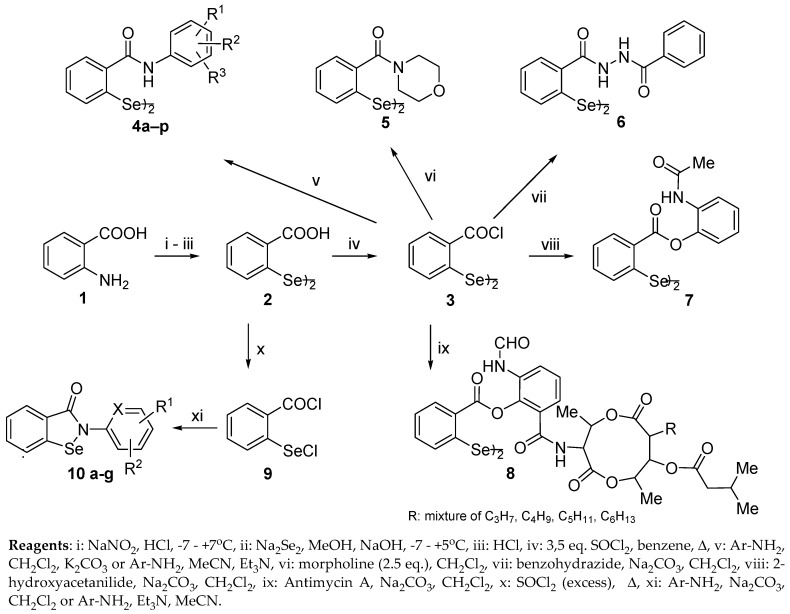
Synthesis of bis(2-carbamoy)diphenyl diselenides **4a**–**p**, **5** and **6**, phenolic esters **7**–**8**, and benzisoselenazol-3(2*H*)-ones **10a**–**g**.

Herein we present our investigation of synthetic route to diselenides **4**–**8** via a reaction of (bis[(2-chlorocarbonyl)phenyl] diselenide), hereafter called chloride **3**, with *N* and *O* nucleophiles, such as a series of various aminophenols and their derivatives as well as other specific reactants such as morpholine, benzohydrazide, 2-hydroxyacetanilide, and antimycin A (Scheme 1, Table 1). The key chloride **3** was prepared in a three-step synthesis starting from anthranilic acid **1**, which was diazotized and reacted with disodium diselenide in a methanolic alkaline medium to give, after acidification, 2,2’-diselenobisbenzoic acid **2** which was further reacted with thionyl chloride [59,60,62].

In a first step, reaction of chloride **3** was tested with various free and protected aminophenols, such as monohydroxyanilines, monomethoxyanilines, and their derivatives containing additional substituents in the benzene ring, such as electron-donating methyl and methoxy groups and electron-withdrawing chloro and carboxymethyl groups. As in the case of 2-(chloroseleno)benzoyl chloride **9**, which was intensively studied with various bisnucleophiles [55], the reaction occurred on the amine group of both protected (**4k**–**p**) and free phenols (**4a**–**j**). In the IR spectra, there were observed signals from primary amide bonds at ca. 3300–3500 cm^−1^ (N–H), carbonyl groups at ca. 1600–1650 cm^−1^ (C=O), amide groups at ca. 1500–1550 cm^−1^ (CONH), and in ^1^H-NMR there were present protons of N–H group at ca. 9.6–10.5 ppm (see the Supplementary Materials for details). All compounds were prepared with very good to excellent yields, significantly higher than those achieved by the reductive ring opening method [53]. The simplest three isomeric aminophenols reacted with 73–92% yield towards **4a**–**c**. Reaction with 2-aminophenols containing electron-withdrawing substituents (Cl and COOMe) at the vicinity carbon atom generally resulted in slightly lower yields (**4g**–**i**; 57–63%), when compared to those containing electron-donating ones (**4d**–**f**; 60–89%). As expected [35], methoxy derivatives **4j**–**p** were also prepared with very good yields (72–99%). As a result of our studies, seven new open-chain analogues of ebselenols **4d**–**j** and two new methoxy derivatives **4n** and **4p** were developed.

Reaction of benzoyl chloride **3** with secondary amines such as morpholine also resulted in secondary amide bond formation to produce compound **5** with a very good yield of 85%. A similar compound containing piperazine moiety was prepared with a comparable yield [64].

Another highly nucleophilic benzohydrazide reacted quantitatively with chloride **3** on its terminal nitrogen atom to form bishydrazide **6**. Encouraged by this result, we continued our studies with other *N*-nucleophiles, focusing on hydrazine, ethylene diamine, tri(hydroxymethyl)aminomethane, and isoniazyde (isonicotinic hydrazide). The resulting oligomeric products were, however, difficult to identify.

Further efforts were directed into the investigation of the behavior of aminophenol derivatives containing blocked amine groups such a 2-hydroxyacetanilide and natural antimycin A. In this case, reaction occurred on the free hydroxyl group only, resulting in phenolic ester derivatives **7** and **8**, however, highly sterically hindered antimycin A required an extremely long reaction time. In the IR spectrum of compound **7**, a characteristic phenolic ester bond was observed at 1760 cm^−1^. Antimycin A is a mixture of at least four closely related compounds, containing alkyl or acyl substituents of different lengths or degrees of branching (usually C_3_–C_6_) in the dilactone portion of the molecule [65]. Thus, the spectroscopic analysis is complicated and only diagnostic signals could be reported in ^13^C-NMR spectra. Fortunately, in HRMS spectra, seven diagnostic molecular species (M + Na^+^) were observed with R=C_5_H_11_ being a predominant one, which was consistent with the results of the elemental analysis.

Ebselen and several other benzisoselenazol-3(2*H*)-ones **10a**–**g**, including two new molecules, **10f** and **10g**, were prepared as a reference for biological tests. The 2,2’-diselenobisbenzoic acid **2** was converted to the 2-(chloroseleno)benzoyl chloride **9** that, upon reaction with corresponding anilines, formed ebselen and benzisoselenazol-3(2*H*)-ones **10a**–**g** through a tandem selenenylation-acylation sequence (Scheme 1, Table 1) [55,59]. To our best knowledge, compounds **4d**–**j**, **4n**, **4p**, **5**–**8**, **10f**–**g** are new structures and were not previously described in the literature.

### 2.2. Antimicrobial Activity

The in vitro antimicrobial activity of compounds **4**–**7**, **10** was screened against four Gram-positive bacterial strains (*Staphylococcus aureus*, *Staphylococcus epidermidis*, *Enterococcus faecalis*, *Enterococcus hirae*), two Gram-negative bacterial strains (*Escherichia coli*, *Pseudomonas aeruginosa*), two yeast-like fungi (*Candida albicans*, *Candida glabrata*), and filamentous fungus (*Aspergillus niger*) in a range of 0.125–128 µg/mL. The minimum inhibitory concentration values (MIC) for organoselenium compounds are summarized in Table 2 and Table 3, along with reference substances.

Generally, there is a noticeable tendency that the compounds tested have been more active against Gram-positive bacterial strains than against Gram-negative ones. These results are in line with our previous observations [33,34,35] and might indicate the possible mode of action of the whole group. Gram-positive bacteria are more susceptible to plasma membrane-binding agents, absorbing them directly into the plasma membrane, whereas a Gram-negative outer membrane may capture such agents before they penetrate through a cell wall and bind into the inner membrane, which usually results in subsequent cell permeabilization [66]. Using propidium iodide, no significant plasma membrane permeabilization was observed in *S. aureus* after treating with ebselen [67], however, membrane binding is not always sufficient for its permeabilization [68]. Membrane binding agents alter plasma membrane biophysics, which may be toxic itself.

The antibacterial activity towards Gram-positive species varied in a range of 1 to >128 µg/mL. Particularly interesting is the strong activity towards *E. faecalis* and *E. hirae*. These enterococci display highest vulnerability to most of the tested compounds and only compounds **4h**–**l**, **5** and **7** (as well as **4m** in the case of *E. hirae*) exhibit no activity in the tested range of concentrations. Enterococci seem to be significantly more susceptible towards organoselenium compounds than reference antibiotics, with the exception of **4o** and **10d**, displaying same MIC as ampicillin towards *E. faecalis*. This makes organoselenium compounds promising for the treatment of enterococci infections, which although being low virulent bacteria, often cause infections in immunocompromised patients [69]. In the case of staphylococci, it can be noticed that compounds **4a**–**c** and **10a**–**b**, containing hydroxyl substituent in the phenyl ring, were the most active against both *S. aureus* and *S. epidermidis*. Benzisoselenazol-3(2*H*)-ones **10a** and **10b** were slightly more active than their open-chain analogues **4a**–**c**. Only two compounds, **4e** and **10a,** showed inhibitory effect against *E. coli,* similar to commonly known antibiotics. None of the compounds showed activity towards *P. aeruginosa* in the screening range. However, it is worth pointing out that this microorganism was also not susceptible toward the majority of reference antibiotics in the tested range.

Contrary to their cyclic analogues, diselenides appeared to be practically inactive towards both *Candida* spp., with the exception of compound **4e**, which also displayed a high inhibitory effect against Gram-positive bacteria and moderate effects against Gram-negative *E. coli* (Table 1). The probable lack of inhibitory effect of the majority of compounds tested might be due to the fact that the antifungal activity of diphenyl diselenides is dependent on the presence of functional groups [40]. However, diphenyl diselenides may still display high activity in reducing germ tube formation, one of the crucial virulent factors of *C. albicans* [38]. Azoles (ketoconazole and itraconazole), commonly used in antifungal therapy, also did not show activity under the test conditions. However, these two compounds are considered static drugs [70], therefore it is difficult to obtain MIC_90_ values for them. Thus, it is possible that organoselenium compounds which lack inhibitory effects may still display synergistic effects. Benzisoselenazol-3(2*H*)-ones **10a**, **10c**, **10f**–**g**, and **10e** (in the case of *C. albicans* only) inhibited fungal growth in a range of 16–64 µg/mL (Table 2), comparable to nourseothricin. Amphotericin B exhibited a much higher activity than all the benzizsoelenazol-3(2*H*)-ones tested, however, this highly cytotoxic compound is considered as a drug of choice for the most severe fungal infections, when the patients are close to death [71]. The majority of diselenides have shown no activity against *A. niger* in the tested range. Only compound **4e** showed weak activity against this filamentous fungus. Benzisoselenazol-3(2*H*)-ones **4c** and **4f** showed moderate activity towards *A. niger*, comparable to other previously tested cyclic analogues of ebselen [33,34].

The compound **5**, prepared by reaction with a secondary amine, as well as phenolic ester **7** were inactive against all tested microorganisms.

### 2.3. Antiviral Activity

The antiviral activity of compounds **4**, **5**, **7**, and **10** has been evaluated in vitro towards HHV-1 (human herpes virus type 1, *Herpesviridae*, enveloped virus), EMCV (encephalomyocarditis virus, *Picornaviridae*, non-enveloped virus), and VSV (vesicular stomatitis virus, *Rhabdoviridae*, enveloped virus) in the human cell line A549. The minimum inhibitory concentrations (MICs) for the abovementioned viruses along with cytotoxicity against the testing cell line are summarized in Table 4.

The compounds exhibited high antiviral activity against HHV-1 and moderate to high activity against EMCV, but in almost all cases were inactive towards VSV (Table 4). A similar tendency was observed in our previous studies [32,33,34,35]. The anti-HHV-1 activity of most diselenides was in a range of 2–40 μg/mL; only the one containing morpholine moiety showed lower activity at 200 μg/mL. The benzisoselenazol-3(2*H*)-ones also showed high activity against HHV-1, in a range of 2–4 μg/mL, comparable to ebselen. The most promising anti-HHV-1 agents would be compounds **4k**, **4n**, and **10f** with higher chemotherapeutic indices (I), indicating that the concentration active against the virus was lower than that showing a cytotoxic effect. Up to the present time, there were only several organoselenium compounds identified with such high chemotherapeutic indices [33]. Generally, the compounds were less active against EMCV and only compounds **4e**, **4o**–**p**, and **10c** showed activity below 10 μg/mL.

## 3. Materials and Methods

### 3.1. Synthesis

#### General Information

Melting points were determined on an Electrothermal IA 91100 digital melting-point apparatus using the standard open capillary method. ^1^H- and ^13^C-NMR spectra were recorded in CDCl_3_ or DMSO-*d*_6_ on a Bruker DRX 300 Spectrometer (Bruker, Rheinstetten, Germany) (^1^H: 300.1 MHz, ^13^C: 75.4 MHz), on a Joel 400 Spectrometer (Joel, Tokyo, Japan) (^1^H: 399.8 MHz, ^13^C: 100.5 MHz) or on a Bruker Avance 600 Spectrometer (^1^H: 600.6 MHz, ^13^C: 151.0 MHz) at 295 K. Chemical shifts (δ) are given in parts per million (ppm) downfield relative to tetramethylsilane (TMS, Si(CH_3_)_4_), and coupling constants (*J*) are in Hz. Residual solvent central signals were recorded as follows: CDCl_3_, δ_H_ = 7.263, δ_C_ = 77.00; DMSO-*d*_6_, δ_H_ = 2.50, δ_C_ = 39.43. FT-IR spectra were recorded between 4000 and 400 cm^−1^ on a Perkin-Elmer 2000 FT-IR spectrometer. High-resolution mass spectra (HRMS) were recorded on a Waters LCD Premier XE instrument (Waters, Manchester, UK), and only the [M + H]^+^ or [M + Na]^+^ diagnostic molecular species are reported. Preparative column chromatography was performed on Merck Si60 silica gel (63–200 μm) (Sigma-Aldrich, Saint Louis, MO, USA) as the stationary phase. Analytical thin-layer chromatography (TLC) was performed on PET foils precoated with silica gel (Merck silica gel, 60 F254) (Sigma-Aldrich, Saint Louis, MO, USA), and visualised using UV light (λ_max_ = 254 nm), or by staining with iodine vapours. Selenium powder (100 mesh) (Sigma-Aldrich, Saint Louis, MO, USA) used for Na_2_Se_2_ preparation had a purity of ≥99.5%. Acetonitrile (MeCN) (POCH, Gliwice, Poland) and methylene chloride (CH_2_Cl_2_) (POCH, Gliwice, Poland) were distilled over P_2_O_5_, and CH_2_Cl_2_ was stored over sodium hydride (NaH) pills. Triethylamine (Et_3_N) (POCH, Gliwice, Poland), distilled over NaOH, was stored over NaOH pellets. Methanol (MeOH) (POCH, Gliwice, Poland) was refluxed over magnesium shavings (Mg) in the presence of elemental iodine (I_2_) and distilled over Mg(OMe)_2_ was formed and stored over 3A molecular sieves (Sigma-Aldrich, Saint Louis, MO, USA). Diethyl ether (DEE) (POCH, Gliwice, Poland) was distilled over a CaH_2_ and LiAlH_4_ mixture from water batch at ca. 45 °C. Morpholine (POCH, Gliwice, Poland) was distilled over NaOH pellets (POCH, Gliwice, Poland) before use. Anhydrous sodium carbonate (Na_2_CO_3_) (POCH, Gliwice, Poland) was ground in a mortar before use. Antimycin A natural product (Sigma-Aldrich, Saint Louis, MO, USA) was used as a mixture of co-crystalized *n*-alkylated compounds with R = C_3_–C_6_ predominantly. 2-Hydroxy-5-carboxymethylaniline was prepared from 3-amino-4-hydroxybenzoic acid (Sigma-Aldrich, Saint Louis, MO, USA) by direct metoksylation in a hot methanol environment and in the presence of H_2_SO_4_ (POCH, Gliwice, Poland), as mentioned below. Ebselen was prepared according to the procedure described earlier [59]. Other reagents and starting materials were directly used as obtained commercially. Purity of the products was confirmed by the comparison of their melting point with data given in literature and by spectroscopic methods. The new compounds were fully characterized.

*2-Hydroxy-5-carboxymethylaniline*. To 2-amino-4-hydroxybenzoic acid (1.53 g, 10 mmol) in dry MeOH (25 mL) was added H_2_SO_4_ (1.0 mL, 1.8 g, 19 mmol) portionwise during stirring, and the solution formed was stirred in solvent reflux (+65 °C) for 20 h. The reaction mixture was cooled and poured into 0.5% NaOH (200 mL) and kept in an ice/water bath, NaHCO_3_ (3.6 g, 44 mmol) was added and the mixture was washed with DEE (100 mL) followed by the addition of 3.5% HCl to a pH of 8.0, and was then extracted with degased DEE. The collected DEE solution was washed with water, dried over Na_2_SO_4_, and the solvent was distilled off from a water bath to obtain crude 2-hydroxy-5-carboxymethylaniline. Yield: (1.60 g, 96%); m.p.: 114.5–115.0 °C (110 °C [72]); as prisms (benzene, 40 mL/g). IR (KBr, cm^−1^) ν 3390, 3300 (NH), 2500–3200 (br OH), 1702 (C=O), 1610, 1429, 1306, 1294, 1284 (C–N), 1201 and 1112 (C–O), 858, 764, 421. ^1^H-NMR (300 MHz, CDCl_3_:DMSO-*d*_6_, 5:1, *v*/*v*): δ 3.81 (s, 3H, OCH_3_), 4.24 (br s, 2H, NH_2_), 6.75 (d, *J* = 8.2 Hz, 1H, Ar-H), 7.24 (dd, *J* = 8.2 Hz, *J* = 2.0 Hz, 1H, Ar-H), 7.33 (d, *J* = 2.0 Hz, 1H, Ar-H), 9.50 (br s, 1H, OH).

*2,2’-Diselenobisbenzoic acid* **2**. The compound was prepared from anthranilic acid **1** using disodium diselenide with a modified literature procedure [59,60,62]. The anthranilic acid **1** (20.5 g, 0.15 mol) was dissolved in a solution of 37% aq. HCl (30 mL) in distilled water (90 mL) at ca. 75 °C. The solution was cooled in an ice-water bath (0–5 °C) to form anthranilic acid hydrochloride precipitate. To the resulting thick suspension, a cold solution of NaNO_2_ (11.4 g, 0.165 mol) in distilled water (90 mL) at ca. −5 °C was added while stirring for 0.5 h, and the reaction was continued for an additional 15 min. The prepared cold solution of anthranilic acid diazonium chloride was added dropwise for 2.5 h to the freshly prepared methanolic solution of disodium diselenide (NaSeSeNa) at −10–7 °C during stirring with nitrogen evolution accompanied by the formation of red selenium . The reaction mixture was warmed to RT (room temperature) and stored overnight. (NaSeSeNa solution preparation: to the suspension of selenium element (11.2 g, 0.15 mol) in NaOH (18 g, 0.45 mol) solution with MeOH (300 mL) in a round-bottom flask equipped with an oil closure, hydrazine monohydrate (1.9 mL, 0.040 mol) was added and gently stirred at RT for 60 h.) The formed grey selenium was filtered, washed with water, and air-dried to obtain selenium powder (6.20 g, 0.083 mol) with 55% yield, which can be reused. The collected filtrates were acidified with 37% aq. HCl (25 mL), gently heated with mixing and left at RT overnight. The solid was filtered off, washed with hot water (ca. 1 L) (until no odor of salicylic acid in the filtrate was detected), and air-dried. The crude product was recrystallized from hot 1,4-dioxane (400 mL) by slow concentration under ca. 200 mmHg to give acid **2**. Yield: (11.1 g, 37%); m.p.: 298–300 °C (300–303 °C [59]); as a pale brick powder (1,4-dioxane).

*Bis[(2-chlorocarbonyl)phenyl)] diselenide* **3**. To 2,2’-diselenobisbenzoic acid (**2**) (20 g, 50 mmol) suspended in benzene (300 mL) in a round-bottom flask equipped with an oil closure and gas trapping, thionyl chloride (SOCl_2_) (15.5 g, 0.13 mol) was added. The mixture was gently refluxed at ca. +80 °C with stirring for 4 h and the evolution of gaseous HCl and SO_2_, following the method previously reported [60]. Benzene and excess of SOCl_2_ were removed under reduced pressure, then the residue was washed with dry n-hexane (3 × 25 mL) and crystallized from dry CH_2_Cl_2_ (15 mL/g) to obtain **3**. Yield: (16.4 g, 75%); m.p.: 174–175 °C (m.p.: 174–175 °C [60]); as green yellow prisms.

*General procedure for the synthesis of bis(2-carbamoylaryl)phenyldiselenides* **4a**–**p**. To a stirred mixture of substituted aniline (1.05 mmol) and sodium carbonate (Na_2_CO_3_) powdered in a mortar (0.265 g, 2.5 mmol) in dry CH_2_Cl_2_ (5 mL), the solution of bis[(2-chlorocarbonyl)phenyl)] diselenide (**3**) (0.218 g, 0.50 mmol) in dry CH_2_Cl_2_ was added dropwise for 30 min at RT. The reaction progress was monitored using TLC (CHCl_3_:EtOAc, 4:1, *v*/*v*). The stirring was continued for 24–168 h. Over a 3-day period, chloride **3** (10.9 mg, 25.0 μmol) was added. When the reaction was completed, the product was filtered off and washed with CH_2_Cl_2_ (2 × 5 mL), water (5 mL), and 3.5% aq. HCl (3 × 5 mL), and air-dried to obtain products **4a**–**p**.

*Bis[2-(2-hydroxyphenylcarbamoyl)]phenyl Diselenide* **4a**. The general procedure starting from 2-hydroxyaniline (0.115 g, 1.05 mmol) at RT was employed, with a 96-h reaction time to obtain **4a**. Yield: (0.28 g, 92% calculated on 2-hydroxyaniline used); m.p.: 233.5–236.5 °C (235–238 °C [53]); as a white solid (MeOH, 0.45 g/L). IR (KBr, cm^−1^) ν 3403 (NH), 3176 (br OH), 1636 (C=O), 1549 (CONH), 1456, 1351, 1286 (C–N), 1235 (C–O), 1194, 1029, 757, 729, 622 (C–Se), 594. ^1^H-NMR (300 MHz, DMSO-*d*_6_): δ 6.86 (ddd, *J* = 7.8 Hz, *J* = 7.3 Hz, *J* = 1.0 Hz, 2H, Ar-H), 6.95 (dd, *J* = 7.5 Hz, *J* = 1.0 Hz, 2H, Ar-H), 7.09 (ddd, *J* = 7.5 Hz, *J* = 7.3 Hz, *J* = 1.5 Hz, 2H, Ar-H), 7.40 (ddd, *J* = 7.3 Hz, *J* = 6.5 Hz, *J* = 1.5 Hz, 2H, Ph-H), 7.46 (ddd, *J* = 7.8 Hz, *J* = 6.5 Hz, *J* = 1.5 Hz, 2H, Ph-H), 7.63 (dd, *J* = 7.8 Hz, *J* = 1.5 Hz, 2H, Ar-H), 7.79 (dd, *J* = 7.8 Hz, *J* = 1.5 Hz, 2H, Ph-H), 8.03 (dd, *J* = 7.3 Hz, *J* = 1.5 Hz, 2H, Ph-H), 9.76 (s, 2H, NH), 9.78 (s, 2H, OH). ^13^C-NMR (75 MHz, DMSO-*d*_6_): δ 115.8 (2 × CH), 118.9 (2 × CH), 125.1 (2 × CH), 126.2 (2 × CH), 126.3 (2 × CH), 128.4 (2 × CH), 130.1 (2 × CH), 132.0 (2 × C), 132.1 (2 × CH), 132.2 (2 × C), 133.0 (2 × C), 150.0 (2 × C), 166.1 (2 × C=O). HRMS (TOF MS ESI): *m*/*z* for C_26_H_20_N_2_O_4_Se_2_ + Na^+^ calculated: 604.9654; found: 604.9662.

*Bis[2-(3-hydroxyphenylcarbamoyl)]phenyl Diselenide* **4b**. The general procedure starting from 3-hydroxyaniline (109 mg, 1.00 mmol) in anhydrous MeCN (5 mL) and benzoyl chloride **3** in anhydrous MeCN (10 mL) at RT was employed, with a 60-h reaction time followed by the dropwise addition of H_2_O (10 mL) during stirring. Solid precipitate was filtered off, washed with aq. HCl and EtOH:H_2_O (19:1, *v*/*v*), and air-dried to obtain **4b**. Yield: (244 mg, 84%); m.p.: 285.5–287.0 °C (m.p.: 285–286 °C [53]); as a pale yellow powder. ^1^H-NMR (400 MHz, DMSO-*d*_6_): δ 6.51–6.59 (m, 2H, Ar-H), 7.11–7.19 (m, 4H, Ar-H), 7.37–7.39 (m, 2H, Ar-H), 7.40 (ddd, *J* = 7.5 Hz, *J* = 7.4 Hz, *J* = 1.0 Hz, 2H, Ph-H), 7.46 (ddd, *J* = 7.9 Hz, *J* = 7.4 Hz, *J* = 1.4 Hz, 2H, Ph-H), 7.79 (dd, *J* = 7.9 Hz, *J* = 1.0 Hz, 2H, Ph-H), 7.93 (dd, *J* = 7.5 Hz, *J* = 1.4 Hz, 2H, Ph-H), 9.50 (s, 2H, OH), 10.45 (s, 2H, NH). ^13^C-NMR (101 MHz, DMSO-*d*_6_): δ 107.56 (2 × CH), 111.23 (4 × CH), 126.38 (2 × CH), 128.60 (2 × CH), 129.35 (2 × CH), 130.15 (2 × CH), 131.93 (2 × CH), 131.95 (2 × C), 133.93 (2 × C), 139.65 (2 × C), 157.55 (2 × C), 166.23 (2 × C=O). HRMS (TOF MS ESI): *m*/*z* for C_26_H_20_N_2_O_4_Se_2_ + Na^+^ calculated: 604.9654; found: 604.9660. Anal. Calcd. for C_26_H_20_N_2_O_4_Se_2_ (582.37): C, 53.62; H, 3.46; N, 4.81. Found: C, 53.87; H, 3.25; N, 4.89.

*Bis[2-(4-hydroxyphenylcarbamoyl)]phenyl Diselenide* **4c**. The general procedure starting from 4-hydroxyaniline (0.115 g, 1.05 mmol) at RT was employed, with a 120-h reaction time to obtain **4c** as a beige powder. Yield: (222 mg, 73% calculated on the 4-hydroxyaniline used). An analytically pure sample was obtained by column chromatography on silica gel using a mixture of CHCl_3_ and EtOAc (4:1, *v*/*v*) as an eluent to obtain pure **4c**; m.p.: 271–272 °C (282–284 °C [53]); as a pale solid (CH_2_Cl_2_). IR (KBr, cm^−1^) ν 3260 (br NH), 3071 (br OH), 1640 (C=O), 1619, 1589, 1510 (CONH), 1444, 1352, 1280 (C–N), 1235 (C–O), 828, 732, 670 (C–Se), 517. ^1^H-NMR (300 MHz, DMSO-*d*_6_): δ 6.83 (d, *J* = 8.7 Hz, 4H, Ar-H), 7.35 (d, *J* = 8.7 Hz, 4H, Ar-H), 7.46 (dd, *J* = 7.5 Hz, *J* = 7.2 Hz, 2H, Ph-H), 7.66 (dd, *J* = 8.0 Hz, *J* = 7.2 Hz, 2H, Ph-H), 7.87 (d, *J* = 7.5 Hz, 2H, Ph-H), 8.06 (d, *J* = 8.0 Hz, 2H, Ph-H), 9.60 (s, 4H, 2 × NH and 2 × OH). ^13^C-NMR (75 MHz, DMSO-*d*_6_): δ 115.49 (4 × CH), 125.67 (2 × CH), 126.04 (2 × CH), 126.70 (4 × CH), 127.74 (2 × CH), 128.21 (2 × C), 130.53 (2 × C), 131.84 (2 × CH), 138.91 (2 × C), 155.66 (2 × C), 164.82 (2 × C=O). HRMS (TOF MS ESI): *m*/*z* for C_26_H_20_N_2_O_4_Se_2_ + Na^+^ calculated: 606.9646; found: 606.9676.

*Bis[2-(2-hydroxy-4-methylphenylcarbamoyl)]phenyl Diselenide* **4d**. The general procedure starting from 2-hydroxy-4-methylaniline (129 mg, 1.05 mmol) at RT was employed, with a 24-h reaction time to obtain **4d**. Yield: (183 mg, 60% calculated on benzoyl chloride **3** used); m.p.: 221.0–223.5 °C; as a beige powder (CH_2_Cl_2_). IR (KBr, cm^−1^) *ν* 3394 (NH), 3250 (br OH), 1640 (C=O), 1603, 1541 (CONH), 1288 (C–N), 1228 (C–O), 809, 736, 647 (C–Se), 589. ^1^H-NMR (300 MHz, DMSO-*d*_6_): δ 2.25 (s, 6H, CH_3_), 6.66 (dd, *J* = 8.2 Hz, *J* = 1.2 Hz, 2H, Ar-H), 6.76 (d, *J* = 1.2 Hz, 2H, Ar-H), 7.39 (ddd, *J* = 7.2 Hz, *J* = 7.0 Hz, *J* = 1.0 Hz, 2H, Ph-H), 7.43 (d, *J* = 8.2 Hz, 2H, Ar-H), 7.44 (ddd, *J* = 7.8 Hz, *J* = 7.0 Hz, *J* = 1.6 Hz, 2H, Ph-H), 7.77 (dd, *J* = 7.8 Hz, *J* = 1.0 Hz, 2H, Ph-H), 8.00 (dd, *J* = 7.2 Hz, *J* = 1.6 Hz, 2H, Ph-H), 9.60 (s, 2H, OH), 9.72 (s, 2H, NH). ^13^C-NMR (75 MHz, DMSO-*d*_6_): δ 20.7 (2 × CH_3_), 116.4 (2 × CH), 119.5 (2 × CH), 122.5 (2 × C), 125.0 (2 × CH), 126.3 (2 × CH), 128.4 (2 × CH), 130.0 (2 × CH), 131.9 (2 × CH), 132.2 (2 × C), 133.0 (2 × C), 135.7 (2 × C), 149.9 (2 × C), 166.1 (2 × C=O). HRMS (TOF MS ESI): *m*/*z* for C_28_H_24_N_2_O_4_Se_2_ + Na^+^ calculated: 634.9959; found: 634.9949. Anal. Calcd. for C_28_H_24_N_2_O_4_Se_2_ (610.42): C, 55.09; H, 3.96; N, 4.59. Found: C, 55.18; H, 3.82; N, 4.52.

*Bis[2-(2-hydroxy-5-methylphenylcarbamoyl)]phenyl Diselenide* **4e**. The general procedure starting from 2-hydroxy-5-methylaniline (129 mg, 1.05 mmol) and benzyl chloride **3** (240 mg, 0.55 mmol) at RT was employed, with a 96-h reaction time to obtain **4e**. Yield: (285 mg, 89% calculated on 2-hydroxy-5-methylaniline); m.p.: 191.5–194.5 °C; as a beige powder (CH_2_Cl_2_). IR (KBr, cm^−1^) ν 3409 (NH), 3235 (br OH), 1640 (C=O), 1599, 1547 (CONH), 1504, 1279 (C–N), 1236 (C–O), 1200, 1027, 809, 740, 619 (C–Se), 452. ^1^H-NMR (300 MHz, DMSO-*d*_6_): δ 2.25 (s, 6H, CH_3_), 6.83 (d, *J* = 8.2 Hz, 2H, Ar-H), 6.89 (dd, *J* = 8.2 Hz, *J* = 1.7 Hz, 2H, Ar-H), 7.39 (ddd, *J* = 7.5 Hz, *J* = 7.1 Hz, *J* = 1.0 Hz, 2H, Ph-H), 7.46 (d, *J* = 1.7 Hz, 2H, Ar-H), 7.47 (ddd, *J* = 7.8 Hz, *J* = 7.1 Hz, *J* = 1.4 Hz, 2H, Ph-H), 7.78 (dd, *J* = 7.8 Hz, *J* = 1.0 Hz, 2H, Ph-H), 8.00 (dd, *J* = 7.5 Hz, *J* = 1.4 Hz, 2H, Ph-H), 9.49 (s, 2H, NH), 9.71 (s, 2H, OH). ^13^C-NMR (75 MHz, DMSO-*d*_6_): δ 20.2 (2 × CH_3_), 115.7 (2 × CH), 124.8 (2 × C), 125.2 (2 × CH), 126.4 (2 × CH), 126.5 (2 × CH), 127.5 (2 × C), 128.4 (2 × CH), 130.1 (2 × CH), 132.0 (2 × CH), 132.2 (2 × C), 133.0 (2 × C), 147.5 (2 × C), 166.1 (2 × C=O). HRMS (TOF MS ESI): *m*/*z* for C_28_H_24_N_2_O_4_Se_2_ + Na^+^ calculated: 632.9967; found: 632.9987. Anal. Calcd. for C_28_H_24_N_2_O_4_Se_2_ (610.42): C, 55.09; H, 3.96; N, 4.59. Found: C, 55.01; H, 3.88; N, 4.64.

*Bis[2-(2-hydroxy-6-methylphenylcarbamoyl)]phenyl Diselenide* **4f**. The general procedure starting from 2-hydroxy-6-methylaniline (129 mg, 1.05 mmol) at RT was employed, with a 168-h reaction time to obtain **4f**. Yield: (282 mg, 88% calculated on 2-hydroxy-6-methylaniline used); m.p.: 264–267 °C; as a pale beige powder (CH_2_Cl_2_). IR (KBr, cm^−1^) ν 3360 (NH), 3251 (OH), 1622 (C=O), 1592, 1515 (CONH), 1472, 1294 (C–N), 1191 (C–O), 1026, 781, 737, 549 (C–Se). ^1^H-NMR (300 MHz, DMSO-*d*_6_): δ 2.19 (s, 6H, CH_3_), 6.74 (d, *J* = 7.7 Hz, 2H, Ar-H), 6.78 (d, *J* = 8.2 Hz, 2H, Ar-H), 7.05 (dd, *J* = 8.2 Hz, *J* = 7.7 Hz, 2H, Ar-H), 7.39 (dd, *J* = 7.6 Hz, *J* = 6.0 Hz, 2H, Ph-H), 7.45 (dd, *J* = 6.8 Hz, *J* = 6.0 Hz, 2H, Ph-H), 7.77 (d, *J* = 7.6 Hz, 2H, Ph-H), 8.09 (d, *J* = 6.8 Hz, 2H, Ph-H), 9.39 (s, 2H, NH), 9.78 (s, 2H, OH). ^13^C-NMR (75 MHz, DMSO-*d*_6_): δ 17.9 (2 × CH_3_), 113.5 (2 × CH), 120.4 (2 × CH), 123.4 (2 × C), 126.1 (2 × CH), 127.3 (2 × CH), 128.5 (2 × CH), 129.9 (2 × CH), 131.7 (2 × CH), 132.4 (2 × C), 133.0 (2 × C), 136.8 (2 × C), 153.3 (2 × C), 166.5 (2 × C=O). HRMS (TOF MS ESI): *m*/*z* for C_28_H_24_N_2_O_4_Se_2_ + Na^+^ calculated: 632.9967; found: 632.9971. Anal. Calcd. for C_28_H_24_N_2_O_4_Se_2_ (610.42): C, 55.09; H, 3.96; N, 4.59. Found: C, 55.16; H, 4.09; N, 4.45.

*Bis[2-(2-hydroxy-4-chlorophenylcarbamoyl)]phenyl Diselenide* **4g**. The general procedure starting from 2-hydroxy-4-chloroaniline (151 mg, 1.05 mmol) at RT was employed, with a 96-h reaction time to obtain **4g**. Yield: (202 mg, 59% calculated on 2-hydroxy-4-chloroaniline used); m.p.: 230–234 °C; as a brownish gray powder (CH_2_Cl_2_). IR (KBr, cm^−1^) ν 3398 (NH), 3176 (br OH), 1639 (C=O), 1532 (CONH), 1414, 1342, 1265 (C–N), 1226 (C–O), 911, 734, 648, 590 (C–Se), 452. ^1^H-NMR (300 MHz, DMSO-*d*_6_): δ 6.92 (dd, *J* = 8.5 Hz, *J* = 2.2 Hz, 2H, Ar-H), 6.98 (d, *J* = 2.2 Hz, 2H, Ar-H), 7.40 (dd, *J* = 7.2 Hz, *J* = 7.0 Hz, 2H, Ph-H), 7.46 (ddd, *J* = 7.9 Hz, *J* = 7.0 Hz, *J* = 1.2 Hz, 2H, Ph-H), 7.63 (d, *J* = 8.5 Hz, 2H, Ar-H), 7.76 (d, *J* = 7.9 Hz, 2H, Ph-H), 8.02 (dd, *J* = 7.2 Hz, *J* = 1.2 Hz, 2H, Ph-H), 9.86 (s, 2H, NH), 10.37 (s, 2H, OH). ^13^C-NMR (75 MHz, DMSO-*d*_6_): δ 115.5 (2 × CH), 118.7 (2 × CH), 124.3 (2 × C), 126.4 (2 × CH), 126.6 (2 × CH), 128.6 (2 × CH), 129.6 (2 × C), 130.0 (2 × CH), 132.1 (2 × CH), 132.3 (2 × C), 132.7 (2 × C), 151.3 (2 × C), 166.2 (2 × C=O). HRMS (TOF MS ESI): *m*/*z* for C_26_H_18_Cl_2_N_2_O_4_Se_2_ + Na^+^ calculated: 672.8874; 674.8866; found: 672.8876; 674.8884. Anal. Calcd. for C_26_H_18_Cl_2_N_2_O_4_Se_2_ (651.26): C, 47.95; H, 2.79; N, 4.30. Found: C, 48.08; H, 2.54; N, 4.50.

*Bis[2-(2-hydroxy-5-chlorophenylcarbamoyl)]phenyl Diselenide* **4h**. The general procedure starting from 2-hydroxy-5-chloroaniline (151 mg, 1.05 mmol) at RT was employed, with a 48-h reaction time to obtain **4h**. Yield: (206 mg, 63% calculated on benzoyl chloride **3**); m.p.: 219.5–221.5 °C; as a gray-brown powder (CH_2_Cl_2_). IR (KBr, cm^−1^) ν 3400 (NH), 3218 (br OH), 1638 (C=O), 1596, 1543 (CONH), 1428, 1270, 1239 (C–N), 1195 (C–O), 1027, 731 (C–Cl), 655 (C–Se), 614. ^1^H-NMR (400 MHz, DMSO-*d*_6_): δ 6.96 (d, *J* = 8.7 Hz, 2H, Ar-H), 7.13 (dd, *J* = 8.7 Hz, *J* = 2.6 Hz, 2H, Ar-H), 7.41 (dd, *J* = 7.6 Hz, *J* = 7.3 Hz, 2H, Ph-H), 7.47 (dd, *J* = 7.9 Hz, *J* = 7.3 Hz, 2H, Ph-H), 7.77 (d, *J* = 2.6 Hz, 2H, Ar-H), 7.78 (d, *J* = 7.6 Hz, 2H, Ph-H), 8.01 (d, *J* = 7.9 Hz, 2H, Ph-H), 9.82 (s, 2H, OH), 10.18 (s, 2H, NH). ^13^C-NMR (101 MHz, DMSO-*d*_6_): δ 116.89 (2 × CH), 121.93 (2 × C), 124.06 (2 × CH), 125.56 (2 × CH), 126.38 (2 × C), 126.41 (2 × CH), 128.61 (2 × CH), 130.10 (2 × CH), 132.18 (2 × CH), 132.30 (2 × C), 132.68 (2 × C), 148.76 (2 × C), 166.19 (2 × C=O). HRMS (TOF MS ESI): *m*/*z* for C_26_H_18_Cl_2_N_2_O_4_Se_2_ + Na^+^ calculated: 674.8866; found: 674.8862. Anal. Calcd. for C_26_H_18_Cl_2_N_2_O_4_Se_2_ (651.26): C, 47.95; H, 2.79; N, 4.30. Found: C, 48.03; H, 2.56; N, 4.25.

*Bis[2-(2-hydroxy-5-carboxymethylphenylcarbamoyl)]phenyl Diselenide* **4i**. The general procedure starting from 2-hydroxy-5-carboxymethylaniline (175 mg, 1.05 mmol) at RT was employed, with a 72-h reaction time to obtain **4i**. Yield: (209 mg, 57% calculated on 2-hydroxy-5-carboxymethylaniline used); m.p.: 281–282 °C; as a cream powder (CH_2_Cl_2_). IR (KBr, cm^−1^) ν 3323 (br NH and OH), 1709, 1691 (OC=O), 1639 (NC=O), 1604, 1537 (CONH), 1430, 1298 (C–N), 1236 (C–O), 1120 (C–O), 737, 606 (C–Se). ^1^H-NMR (400 MHz, DMSO-*d*_6_): δ 3.83 (s, 6H, OCH_3_), 7.05 (d, *J* = 8.5 Hz, 2H, Ar-H), 7.41 (ddd, *J* = 7.6 Hz, *J* = 7.3 Hz, *J* = 1.2 Hz, 2H, Ph-H), 7.48 (ddd, *J* = 7.9 Hz, *J* = 7.3 Hz, *J* = 1.2 Hz, 2H, Ph-H), 7.74 (dd, *J* = 8.5 Hz, *J* = 2.2 Hz, 2H, Ar-H), 7.79 (dd, *J* = 7.9 Hz, *J* = 1.2 Hz, 2H, Ph-H), 8.03 (dd, *J* = 7.6 Hz, *J* = 1.2 Hz, 2H, Ph-H), 8.31 (d, *J* = 2.2 Hz, 2H, Ar-H), 9.89 (s, 2H, NH), 10.86 (s, 2H, OH). ^13^C-NMR (101 MHz, DMSO-*d*_6_): δ 51.76 (2 × OCH_3_), 115.58 (2 × CH), 120.14 (2 × C), 125.09 (2 × C), 126.27 (2 × CH), 126.42 (2 × CH), 127.95 (2 × CH), 128.61 (2 × CH), 130.10 (2 × CH), 132.16 (2 × CH), 132.30 (2 × C), 132.74 (2 × C), 154.64 (2 × C), 165.83 (2 × C=O), 166.25 (2 × C=O). HRMS (TOF MS ESI): *m*/*z* for C_30_H_24_N_2_O_8_Se_2_ + Na^+^ calculated: 720.9763; found: 720.9755. Anal. Calcd. for C_30_H_24_N_2_O_8_Se_2_ (698.44): C, 51.59; H, 3.46; N, 4.01. Found: C, 51.43; H, 3.60; N, 3.95.

*Bis[2-(3-hydroxy-4-methoxyphenylcarbamoyl)]phenyl Diselenide* **4j**. The general procedure starting from 3-hydroxy-4-methoxyaniline (146 mg, 1.05 mmol) at RT was employed, with a 120-h reaction time to obtain **4j**. Yield: (317 mg, 94% calculated on 3-hydroxy-4-methoxyaniline used); m.p.: 232.5–236.5 °C; as a white powder (CH_2_Cl_2_). IR (KBr, cm^−1^) ν 3500 (NH), 3270 (OH), 1670, 1636 (C=O), 1513 (CONH), 1270 (C–N), 1239 (C–O), 1175 (C–O), 1024 (C–O), 738, 565 (C–Se). ^1^H-NMR (300 MHz, DMSO-*d*_6_): δ 3.76 (s, 6H, 2OCH_3_), 6.91 (d, *J* = 8.8 Hz, 2H, Ar-H), 7.13 (dd, *J* = 8.8 Hz, *J* = 2.5 Hz, 2H, Ar-H), 7.37 (d, *J* = 2.5 Hz, 2H, Ar-H), 7.36–7.46 (m, 4H, Ph-H), 7.78 (dd, *J* = 7.7 Hz, *J* = 1.2 Hz, 2H, Ph-H), 7.93 (dd, *J* = 7.3 Hz, *J* = 1.4 Hz, 2H, Ph-H), 7.06 (br s, 2H, OH), 10.32 (s, 2H, NH). ^13^C-NMR (75 MHz, DMSO-*d*_6_): δ 55.8 (2 × OCH_3_), 108.9 (2 × CH), 111.4 (2 × CH), 112.3 (2 × CH), 126.3 (2 × CH), 128.4 (2 × CH), 130.0 (2 × CH), 131.7 (2 × CH), 131.9 (2 × C), 132.1 (2 × C), 133.9 (2 × C), 144.4 (2 × C), 146.3 (2 × C), 165.8 (2 × C=O). HRMS (TOF MS ESI): *m*/*z* for C_28_H_24_N_2_O_6_Se_2_ + Na^+^ calculated: 664.9865; 666.9857; found: 664.9854; 666.9868. Anal. Calcd. for C_28_H_24_N_2_O_6_Se_2_ (642.42): C, 52.35; H, 3.77; N, 4.36. Found: C, 52.30; H, 3.52; N, 4.45.

*Bis[2-(2-methoxyphenylcarbamoyl)]phenyl Diselenide* **4k**. The general procedure starting from 2-methoxyaniline (129 mg, 1.05 mmol) at RT was employed, with a 168-h reaction time to obtain **4k**. Yield: (0.301 g, 94% calculated on 2-methoxyaniline used); m.p.: 214–215 °C (m.p.: 216–217 °C [53]); as yellow crystals (MeOH, 2 L/g). IR (KBr, cm^−1^) ν 3411 (NH), 1646 (C=O), 1602, 1528 (CONH), 1462, 1434, 1340, 1289 (C–N), 1251 and 1026 (C–O), 745, 629 (C–Se), 591, 561, 457. ^1^H-NMR (300 MHz, DMSO-*d*_6_): δ 3.84 (s, 6H, OCH_3_), 7.00 (ddd, *J* = 7.7 Hz, *J* = 7.4 Hz, *J* = 1.0 Hz, 2H, Ar-H), 7.13 (dd, *J* = 8.3 Hz, *J* = 1.0 Hz, 2H, Ar-H), 7.24 (ddd, *J* = 8.3 Hz, *J* = 7.4 Hz, *J* = 1.6 Hz, 2H, Ar-H), 7.40 (dd, *J* = 7.4 Hz, *J* = 7.4 Hz, 2H, Ph-H), 7.47 (dd, *J* = 7.9 Hz, *J* = 7.4 Hz, 2H, Ph-H), 7.73 (dd. *J* = 7.7 Hz, *J* = 1.6 Hz, 2H Ar-H), 7.77 (d, *J* = 7.9 Hz, 2H, Ph-H), 8.01 (d, *J* = 7.4 Hz, 2H, Ph-H), 9.83 (s, 2H, NH). ^13^C-NMR (75 MHz, DMSO-*d*_6_): δ 55.56 (2 × OCH_3_), 111.56 (2 × C), 120.17 (2 × C), 125.15 (2 × C), 126.16 (2 × C), 126.39 (2 × C), 126.42 (2 × C), 128.49 (2 × C), 130.06 (2 × C), 132.04 (2 × C), 132.30 (2 × C), 132.96 (2 × C), 152.04 (2 × C), 166.12 (2 × C=O). The HRMS (TOF MS ESI) is in agrement with literature data [26].

*Bis[2-(3-methoxyphenylcarbamoyl)]phenyl Diselenide* **4l**. Preparation according to Reference [35]. The general procedure starting from 3-methoxyaniline (129 mg, 1.05 mmol) at RT was employed, with a 72-h reaction time to obtain **4l**. Yield: (0.300 g, 98% calculated on benzoyl chloride **3** used); m.p.: 221–223 °C (221–223 °C [35]); as a colorless powder (CHCl_3_:EtOAc, 1:2, *v*/*v*). Yield of the analytically pure sample: (226 mg, 74%). The ^1^H-NMR, ^13^C-NMR, and HRMS (TOF MS ESI) spectra are in agreement with literature data [35].

*Bis[2-(4-methoxyphenylcarbamoyl)]phenyl Diselenide* **4m**. Preparation according to a modified literature procedure [35]. The general procedure starting from 4-methoxyaniline (129 mg, 1.05 mmol) and bis[(2-chlorocarbonyl)phenyl)] diselenide **3** (0.229 g, 0.525 mmol) at RT was employed, with a 48-h reaction time to obtain **4m**. Yield: (0.300 g, 94%); m.p.: 285.5–287.5 °C (290–292 °C [53]); as a colorless powder (CHCl_3_:EtOAc, 1:2, *v*/*v*). Yield of the analytically pure sample: (266 mg, 83%). The ^1^H-NMR, ^13^C-NMR, and HRMS (TOF MS ESI) spectra are in agreement with literature data [35].

*Bis[2-(2,4-dimethoxyphenylcarbamoyl)]phenyl Diselenide* **4n**. The general procedure starting from 2,4-dimethoxyaniline (161 mg, 1.05 mmol) at RT was employed, with a 168-h reaction time, with separation by acidification with 3.5% HCl to a pH of 2.5, extraction with CH_2_Cl_2_, dry with anhydrous Na_2_SO_4_, filtration, and solvent evaporation to obtain **4n**. Yield: (348 mg, 99% calculated on 2,4-dimethoxyaniline used); m.p.: 211.0–213.5 °C; as a white powder (CH_2_Cl_2_). IR (KBr, cm^−1^) ν 3436 (NH), 1640 (C=O), 1531 (CONH), 1414, 1283 (C–N), 1205, and 1036 (C–O), 831, 732, 578 (C–Se), 453. ^1^H-NMR (300 MHz, DMSO-*d*_6_): δ 3.79 (s, 6H, 2OCH_3_), 3.82 (s, 6H, 2OCH_3_), 6.57 (dd, *J* = 8.8 Hz, *J* = 2.6 Hz, 2H, Ar-H), 6.69 (d, *J* = 2.6 Hz, 2H, Ar-H), 7.35–7.48 (m, 4H, Ph-H), 7.47 (d, *J* = 8.8 Hz, 2H, Ar-H), 7.75 (d, *J* = 7.3 Hz, 2H, Ph-H), 8.01 (d, *J* = 7.2 Hz, 2H, Ph-H), 9.76 (s, 2H, NH). ^13^C-NMR (75 MHz, DMSO-*d*_6_): δ 55.33 (2 × OCH_3_), 55.69 (2 × OCH_3_), 98.94 (2 × C), 104.25 (2 × C), 119.06 (2 × C), 126.28 (2 × C), 126.79 (2 × C), 128.39 (2 × C), 129.98 (2 × C), 131.88 (2 × C), 132.30 (2 × C), 132.93 (2 × C), 153.74 (2 × C), 158.28 (2 × C), 166.20 (2 × C=O). HRMS (TOF MS ESI): *m*/*z* for C_30_H_28_N_2_O_6_Se_2_ + Na^+^ calculated: 693.0178; found: 693.0176. Anal. Calcd. for C_30_H_28_N_2_O_6_Se_2_ (670.47): C, 53.74; H, 4.21; N, 4.18. Found: C, 53.62; H, 4.13; N, 4.27.

*Bis[2-(3,4-dimethoxyphenylcarbamoyl)]phenyl Diselenide* **4o**. The general procedure starting from 3,4-dimethoxyaniline (161 mg, 1.05 mmol) at RT was employed, with a 96-h reaction time to obtain **4o**. Yield: (253 mg, 72%); m.p.: 231.5–234.5 °C (m.p.: 235 °C [53]); as a pale yellow powder (CH_2_Cl_2_). IR (KBr, cm^−1^) ν 3291 (NH), 1641 (C=O), 1513 (CONH), 1452, 1263 (C–N), 1237 (C–O), 1134, 1026 (C–O), 741, 681, 574 (C–Se), 454. ^1^H-NMR (300 MHz, DMSO-*d*_6_): δ 3.76 (s, 6H, 2OCH_3_), 3.77 (s, 6H, 2OCH_3_), 6.96 (d, *J* = 8.8 Hz, 2H, Ar-H), 7.30–7.45 (m, 6H, Ph-H and Ar-H), 7.47 (s, 2H, Ar-H), 7.83 (s, br., 2H, Ph-H), 7.95 (d, *J* = 7.7 Hz, 2H, Ph-H), 10.51 (s, 2H, NH). ^13^C-NMR (75 MHz, DMSO-*d*_6_): δ 55.41 (2 × OCH_3_), 55.67 (2 × OCH_3_), 105.81 (2 × CH), 111.84, (2 × CH), 112.72 (2 × CH), 126.08 (2 × CH), 128.36 (2 × CH), 130.10 (2 × CH), 131.59 (2 × CH), 132.19 (2 × C), 132.30 (2 × C), 133.63 (2 × C), 145.46 (2 × C), 148.42 (2 × C), 165.78 (2 × C=O). HRMS (TOF MS ESI): *m*/*z* for C_30_H_28_N_2_O_6_Se_2_ + Na^+^ calculated: 693.0178. Found: 693.0178.

*Bis[2-(3,4,5-trimethoxyphenylcarbamoyl)]phenyl Diselenide* **4p**. The general procedure starting from 3,4,5-trimethoxyaniline (192 mg, 1.05 mmol) at RT was employed, with a 96-h reaction time to obtain **4p**. Yield: (0.291 g, 76%); m.p.: 193–195 °C; as a white powder (CH_3_OH, 100 g/L). IR (KBr, cm^−1^) ν 3322 (NH), 1605 (C=O), 1507 (CONH), 1450, 1411, 1313, 1235 (C–N), 1128 (C–O), 1027 (C–O), 1004 (C–O), 738, 623 (C–Se). ^1^H-NMR (300 MHz, DMSO-*d*_6_): δ 3.65 (s, 6H, 2OCH_3_), 3.77 (s, 12H, 4OCH_3_), 7.20 (s, 4H, Ar-H), 7.38 (dd, *J* = 7.3 Hz, 2H, Ph-H), 7.44 (dd, *J* = 7.3 Hz, 2H, Ph-H), 7.80–8.00 (m, 4H, Ph-H), 10.56 (s, 2H, NH). ^1^H-NMR (600 MHz, DMSO-*d*_6_): δ 3.67 (s, 6H, 2OCH_3_), 3.80 (s, 12H, 4OCH_3_), 7.21 (s, 4H, Ar-H), 7.42 (dd, *J* = 7.3 Hz, *J* = 6.6 Hz, 2H, Ph-H), 7.46 (dd, *J* = 7.9 Hz, *J* = 6.6 Hz, 2H, Ph-H), 7.81 (d, *J* = 7.9 Hz, 2H, Ph-H), 7.95 (d, *J* = 7.3 Hz, 2H, Ph-H), 10.47 (s, 2H, NH). ^13^C-NMR (75 MHz, DMSO-*d*_6_): δ 56.33 (4 × OCH_3_), 60.64 (2 × OCH_3_), 99.21 (4 × CH), 126.6 (2 × CH), 128.9 (2 × CH), 130.5 (2 × CH), 132.1 (2 × CH, 2 × C), 134.4 (2 × C), 134.6 (2 × C), 135.8 (2 × C), 153.2 (4 × C), 166.5 (2 × C=O). ^13^C-NMR (151 MHz, DMSO-*d*_6_): δ 55.75 (4 × OCH_3_), 60.10 (2 × OCH_3_), 98.24 (4 × CH), 126.31 (2 × CH), 128.53 (2 × CH), 130.12 (2 × CH), 131.97 (2 × CH, 2 × C), 133.71 (2 × C), 134.05 (2 × C), 134.75 (2 × C), 152.64 (4 × C), 166.10 (2 × C=O).HRMS (TOF MS ESI): *m*/*z* for C_32_H_32_N_2_O_8_Se_2_ + Na^+^ calculated: 755.0381; found: 755.0381. Anal. Calcd. for C_32_H_32_N_2_O_8_Se_2_ (730.53): C, 52.61; H, 4.42; N, 3.83. Found: C, 52.46; H, 4.56; N, 3.72.

*Bis[2-(N-morpholinecarbamoyl)]phenyl Diselenide* **5**. To a stirred solution of morpholine (436 mg, 0.44 mL, 5.0 mmol) in dry CH_2_Cl_2_ (10 mL), the solution of bis[(2-chlorocarbonyl)phenyl]diselenide **3** (437 mg, 1.0 mmol) in dry CH_2_Cl_2_ was added and the reaction was continued at RT (+20 °C) for 120 h until no spot of chloride **3** was observed during TLC control (silica gel, eluent CHCl_3_:EtOAc, 4:1, *v*/*v*). When the reaction was completed, the mixture was poured into water (50 mL), acidified with 3.5% HCl to a pH of 1.0, extracted with CH_2_Cl_2_ (3 × 25 mL), and dried with Na_2_SO_4_. After evaporation of the solvent, the residue was washed with n-hexane and DEE (25 mL) was added to the oily residue, followed by stirring until the product solidified and was filtered off to obtain **5**. Yield: (458 mg, 85% calculated on diselenide **3** used); m.p.: 149–152 °C; as a pale yellow powder (DEE). IR (KBr, cm^−1^) ν 3059, 2972, 2959, 2902, 2854 (CH), 1625 (C=O), 1608, 1430, 1279, 1248, 1111 (C–N), 1021 (C–O), 1014 (C–O), 757, 596, 559 (C–Se), 461. ^1^H-NMR (300 MHz, DMSO-*d*_6_): δ 3.57 (s, 16H, 8CH_2_), 7.28–7.37 (m, 4H, Ph-H), 7.39 (ddd, *J* = 7.7 Hz, *J* = 6.4 Hz, *J* = 2.7 Hz, 2H, Ph-H), 7.74 (dd, *J* = 8.2 Hz, *J* = 1.2 Hz, 2H, Ph-H). ^13^C-NMR (75 MHz, DMSO-*d*_6_): δ 66.04 (8 × CH_2_), 126.84 (2 × CH), 127.36 (2 × CH), 129.05 (2 × C), 130.38 (2 × CH), 131.71 (2 × CH), 136.22 (2 × C), 167.48 (2 × C=O). HRMS (TOF MS ESI): *m*/*z* for C_22_H_24_N_2_O_4_Se_2_ + Na^+^ calculated: 558.9986; 560.9967; 562.9959; found: 559.0029; 560.9987; 562.9982. Anal. Calcd. for C_22_H_24_N_2_O_4_Se_2_ (538.36): C, 49.08; H, 4.49; N, 5.20. Found: C, 48.96; H, 4.62; N, 5.29.

*Bis[2-(N-benzamidecarbamoyl)]phenyl Diselenide* **6**. The general procedure starting from benzhydrazide (143 mg, 1.05 mmol) at RT was employed, with a 120-h reaction time to obtain **6**. Yield: (335 mg, 100%); m.p.: 296.5 °C (with decomp.); as a pale yellow wool (AcOH, 1.5 g/L). ^1^H-NMR (601 MHz, DMSO-*d*_6_): δ 7.42 (ddd, *J* = 7.7 Hz, *J* = 7.3 Hz, *J* = 0.8 Hz, 2H, Ph-H), 7.50 (ddd, *J* = 8.0 Hz, *J* = 7.3 Hz, *J* = 1.1 Hz, 2H, Ph-H), 7.54 (dd, *J* = 7.8 Hz, *J* = 7.4 Hz, 4H, Ar-H), 7.62 (t, *J* = 7.3 Hz, 2H, Ar-H), 7.78 (dd, *J* = 8.0 Hz, *J* = 0.8 Hz, 2H Ph-H), 7.97 (d, *J* = 7.8 Hz, 4H, Ar-H), 8.00 (dd, *J* = 7.7 Hz, *J* = 1.1 Hz, 2H, Ph-H), 10.68 (s, 2H, NH), 10.83 (s, 2H, NH). ^13^C-NMR (151 MHz, DMSO-*d*_6_): δ 126.40 (2 × CH), 127.45 (4 × CH), 128.30 (2 × CH), 128.51 (4 × CH), 130.12 (2 × CH), 130.70 (2 × C), 131.94 (2 × CH), 132.30 (2 × C), 132.44 (2 × CH), 132.47 (2 × C), 165.77 (2 × C=O), 166.86 (2 × C=O). HRMS (TOF MS ESI): *m*/*z* for C_28_H_22_N_4_O_4_Se_2_ + Na^+^ calculated: 656.9891; 658.9872; found: 656.9968; 658.9862. Anal. Calcd. for C_28_H_22_N_4_O_4_Se_2_ (636.42): C, 52.84; H, 3.48; N, 8.80. Found: C, 53.02; H, 3.29; N, 8.79.

*2-Hydroxyacetanilide phenolic ester of bis(2-carboxy)phenyl diselenide* **7**. The general procedure starting from 2-hydroxyacetanilide (151 mg, 1.0 mmol) dissolved in dry CH_2_Cl_2_ (10 mL) at RT was employed, with a 96-h reaction time to obtain **7**. Yield: (119 mg, 36% calculated on 2-hydroxyacetanilide used); m.p.: 178–181 °C; as a pale solid (CH_2_Cl_2_). IR (KBr, cm^−1^) ν 3402 (NH), 1760 (OC=O), 1659 (NC=O), 1545 (CONH), 1455, 1370, 1285 (C–N), 1216 (C–O), 1116, 767, 622 (C–Se), 457. ^1^H-NMR (300 MHz, DMSO-*d*_6_): δ 2.09 (s, 6H, CH_3_), 6.75 (dd, *J* = 7.9 Hz, *J* = 7.1 Hz, 2H, Ph-H), 6.85 (d, *J* = 7.0 Hz, 2H, Ph-H), 6.93 (dd, *J* = 7.9 Hz, *J* = 6.6 Hz, 2H, Ph-H), 7.36 (dd, *J* = 7.6 Hz, *J* = 7.0 Hz, 2H, Ph-H), 7.49 (dd, *J* = 7.2 Hz, *J* = 7.0 Hz, 2H, Ph-H), 7.66 (d, *J* = 6.6 Hz, 2H, Ar-H), 7.67 (d, *J* = 7.9 Hz, 2H, Ph-H), 8.33 (d, *J* = 7.2 Hz, 2H, Ph-H), 9.72 (s, 2H, NH). HRMS (TOF MS ESI): *m*/*z* for C_30_H_24_N_2_O_6_Se_2_ + Na^+^ calculated: 688.9865; 690.9857; found: 688.9866; 690.9855. Anal. Calcd. for C_30_H_24_N_2_O_6_Se_2_ (666.44): C, 54.07; H, 3.63; N, 4.20. Found: C, 53.85; H, 3.90; N, 4.06.

*(2R,3S,6S,7R,8R)-3-(3-Formamido-2-hydroxybenzamido)-8-n-alkyl-2,6-dimethyl-4,9-dioxo-1,5-dioxonan-7-yl 3-Methylbutanoate (antimycin A) phenolic ester of bis(2-carboxy)phenyl diselenide* **8**. The general procedure starting from naturally occurred antimycin A (0.21 g, 0.40 mmol) dissolved in dry CH_2_Cl_2_ (5 mL), and Na_2_CO_3_ (0.16 g, 1.5 mmol), diselenide **3** (87 mg, 0.20 mmol) at RT was employed, with a 10-day reaction time to obtain phenolic ester **8** as a co-crystallizable mixture of different alkyl substituents with R=C_5_H_11_ predominantly. Yield: (0.277 g, 97% calculated on antimycin A used); m.p.: >300 °C; as a pale solid. In ^13^C-NMR, a number of signals is observed and only diagnostic ones are reported ^13^C-NMR (101 MHz, DMSO-*d*_6_): δ 13.88, 17.72, 18.92, 19.00, 19.02, 20.28, 21.85, 21.94, 22.85, 24.43, 25.66, 26.81, 27.18, 28.10 and 30.89 (C_aliphatic_); 58.05, 58.49, 66.77, 66.80 and 77.35 (CHO and CNO); 107.05, 107.47, 117.11, 117.21, 117.28, 118.94, 124.16, 130.59, 131.94 and 136.79 (C_Ar_, C_Ar_-H); 151.23 (C_Ar_-CON), 166.95, 167.16, 167.26, 167.43, 170.42, 174.84, 174.98, 175.09 and 175.11 (C=O). HRMS (TOF MS ESI): *m*/*z* for C_64_H_74_N_4_O_20_Se_2_ + Na^+^ calculated: 1401.3119; found: 1401.3433; for C_65_H_76_N_4_O_20_Se_2_ + Na^+^ calculated: 1411.3291; 1413.3283; found: 1411.3070; 1413.3112; for C_66_H_78_N_4_O_20_Se_2_ + Na^+^ calculated: 1427.3440; 1429.3432; found: 1427.3264; 1429.3787; for C_67_H_80_N_4_O_20_Se_2_ + Na^+^ calculated: 1441.3596; found: 1441.3926; for C_68_H_82_N_4_O_20_Se_2_ + Na^+^ calculated: 1455.3753; 1457.3745; found: 1455.4171; 1457.4158; for C_69_H_84_N_4_O_20_Se_2_ + Na^+^ calculated: 1469.3909; 1471.3902; found: 1469.4357; 1471.4440; for C_70_H_86_N_4_O_20_Se_2_ + Na^+^ calculated: 1481.4085; 1483.4066; 1485.4058; 1486.4092; found: 1481.3927; 1483.4473; 1485.5210; 1486.4746. Anal. Calcd. for C_68_H_82_N_4_O_20_Se_2_ (1433.31): C, 56.98; H, 5.77; N, 3.91. Found: C, 57.01; H, 5.82; N, 3.77.

*2-(Chloroseleno)benzoyl chloride* **9**. The compound was prepared from 2,2’-diselenobisbenzoic acid **2** (8.0 g, 20 mmol) using excess of thionyl chloride (6.0 g, 50 mmol) and DMF (1.5 g, 20 mmol) in boiling benzene (150 mL), following the method previously reported [60]. Crude oily product was recrystallized from n-hexane (ca. 15 mL/g) and dried under vacuum (20 mmHg) to give **9**. Yield: (8.1 g, 80%); m.p.: 65–67 °C (65–66 °C [60]); as yellow needles (n-hexane).

*General synthetic procedure for benzisoselenazol-3(2H)-ones* **10a**–**g**. To a stirred solution of substituted aniline (13.0 mmol) in anhydrous CH_2_Cl_2_ (5 mL) a solution of (2-chloroseleno)benzoyl chloride **9** (1.02 g, 4.0 mmol) in anhydrous CH_2_Cl_2_ (25 mL) was added dropwise. The reaction was continued for 2–24 h. The progress of reaction was controlled by TLC (silica gel, eluent CHCl_3_:EtOAc, 4:1, *v*/*v*). After the reaction was finished, the solvent was evaporated, HCl (3.5%, w/w, 40 mL) was added, and the mixture was stirred at RT overnight, then filtered off and left to dry in the air. Crude products were purified by column chromatography on silica gel (70–230 mesh) eluted with CHCl_3_ to obtain pure **10a**–**g**.

*2-(2-Hydroxyphenyl)-1,2-benzisoselenazol-3(2H)-one* **10a**. The general procedure starting from 2-hydroxyaniline (0.44 g, 4.0 mmol) and anhydrous Et_3_N (1.01 g, 1.4 mL, 10 mmol) at RT was employed, with a 20-h reaction time with separation of the product from the crude reaction mixture by column chromatography using EtOAc as an eluent to obtain **10a**. Yield: (0.46 g, 40% calculated on benzoyl chloride **9** used); m.p.: 195.5–197.5 °C (194–196 °C [54]); as orange flakes (MeCN:H_2_O, 84:16, *v*/*v*). ^1^H-NMR (400 MHz, CDCl_3_): δ 6.99 (ddd, *J* = 8.0 Hz, *J* = 7.2 Hz, *J* = 1.5Hz, 1H, Ar-H), 7.13 (dd, *J* = 8.6 Hz, *J* = 1.5 Hz, 1H, Ar-H), 7.24–7.30 (m, 2H, Ar-H), 7.47–7.54 (m, 1H, Ph-H), 7.65–7.71 (m, 2H, Ph-H), 8.13 (d, *J* = 8.1 Hz, 1H, Ar-H), 8.55 (s, 1H, OH) [57]. ^13^C-NMR (101 MHz, DMSO-*d*_6_): δ 116.94 (2 × CH), 119.22 (2 × CH), 125.73 (2 × CH), 125.81 (2 × CH), 125.97 (C), 127.52 (C), 127.76 (CH), 128.76 (CH), 129.32 (CH), 131.82 (CH), 140.50 (C), 153.25 (C), 165.76 (C=O) [57]. The ^77^Se NMR, and HRMS are in agreement with literature data [26,57].

*2-(3-Hydroxypyridin-2-yl)-1,2-benzisoselenazol-3(2H)-one* **10b**. The general procedure starting from 2-amino-3-hydroxypyridine (0.44 g, 4.0 mmol) and anhydrous Et_3_N (0.95 g, 1.3 mL, 9.4 mmol) at anhydrous MeCN (40 mL) using benzoyl chloride **9** in anhydrous MeCN was employed for a 2-h reaction time at RT. Solvent was evaporated, water (100 mL) was added portionwise, and the solution was stirred for 1 h. The solid was filtered off, washed with H_2_O, and air-dried to obtain **10b**. Yield: (0.76 g, 65%); m.p.: 229 °C (decomp.) with phase transfer at 199 °C (m.p.: 199–201 °C [55]); as a greenish yellow powder (DMSO). ^1^H-NMR (DMSO-*d*_6_): δ 7.26 (dd, *J* = 8.0 Hz, *J* = 4.6 Hz, 1H, Pyr-H), 7.38 (dd, *J* = 8.0 Hz, *J* = 1.4 Hz, 1H, Pyr-H), 7.50 (ddd, *J* = 7.8 Hz, *J* = 7.2 Hz, *J* = 0.9 Hz, 1H, Ar-H), 7.72 (ddd, *J* = 8.0 Hz, *J* = 7.2 Hz, *J* = 1.3 Hz, 1H, Ar-H), 7.95 (dd, *J* = 7.8 Hz, *J* = 0.9 Hz, 1H Ar-H), 7.98 (dd, *J* = 4.6 Hz, *J* = 1.4 Hz, 1H, Pyr-H), 8.09 (dd, *J* = 8.0 Hz, *J* = 1.3 Hz, 1H, Ar-H), 12.18 (s, 1H, OH). The ^13^C-NMR is in agreement with literature data [55].

*2-(2-Methoxyphenyl)-1,2-benzisoselenazol-3(2H)-one* **10c**. The general procedure starting from 2-methoxyaniline (1.60 g, 13 mmol) at RT was employed, with a 24-h reaction time followed by separation by column chromatography to obtain **10a**. Yield: (0.86 g, 71% calculated on the benzoyl chloride **9**); m.p.: 189.0–191.5 °C (m.p.: 172–174 °C [63]); as yellow prisms (CHCl_3_). IR (KBr, cm^−1^) ν 1621 (C=O), 1270 (C–N), 1108 (C–O), 1020 (C–O). ^1^H-NMR (300 MHz, CDCl_3_) [63]: δ 3.85 (s, 3H, OCH_3_), 6.98–7.07 (m, 2H, Ph-H), 7.36 (ddd, *J* = 8.2 Hz, *J* = 7.6 Hz, *J* = 1.7 Hz, 1H, Ph-H), 7.44 (ddd, *J* = 8.0 Hz, *J* = 6.4 Hz, *J* = 1.7 Hz, 1H, Ar-H), 7.48 (dd, *J* = 8.0 Hz, *J* = 1.7 Hz, 1H, Ph-H), 7.58–7.70 (m, 2H, Ar-H), 8.13 (d, *J* = 7.8 Hz, 1H, Ar-H). ^13^C-NMR (75 MHz, CDCl_3_): δ 55.80 (OCH_3_), 112.22 (CH), 120.74 (CH), 123.86 (CH), 126.01 (CH), 126.44 (C), 126.74 (C), 129.21 (CH), 129.66 (CH), 129.89 (CH), 132.17 (CH), 139.31 (C), 155.39 (C), 166.70 (C=O).

*2-(3-Methoxyphenyl)-1,2-benzoisoselenazole-3(2H)-one* **10d**. Prepared according to a modified procedure [35]. The general procedure starting from 3-methoxyaniline (0.493 g, 4.0 mmol) and dry Et_3_N (1.02 g, 1.4 mL, 10 mmol) in anhydrous MeCN (40 mL) at RT with a 2-h reaction time was employed. Water was added portionwise and the solution was left in the refrigerator for crystallization. Then, the solid was filtered off, washed with water and MeCN, and dried in the air to obtain **10d**. Yield: (0.79 g, 65%), m.p: 166–168 °C (165.5–167.5°C [35]); as yellow prisms in (MeCN:H_2_O, 1:1, *v*/*v*). The ^1^H-NMR and ^13^C-NMR are in agreement with literature data [35].

*2-(4-Methoxyphenyl)-1,2-benzoisoselenazole-3(2H)-one* **10e**. Prepared according to a modified procedure [35]. The general procedure starting from 3-methoxyaniline (0.49 g, 4.0 mmol) and Et_3_N (1.4 mL, 1.02 g, 10 mmol) in anhydrous MeCN (40 mL) at RT with a 20-h reaction time was employed. Water (40 mL) was added portionwise and the solution was left in the refrigerator for crystallization. Then, the solid was filtered off, washed with water, dried in the air, and the crude product was recrystalized from EtOAc (30 mL/g) to obtain **10e**. Yield: (0.85 g, 70%); m.p.: 180–181 °C (180.5–181.5°C [35]); as yellow prisms (EtOAc). The ^1^H-NMR and ^13^C-NMR data are in agreement with literature data [26,35].

*2-(2,4-Dimethoxyphenyl)benzisoselenazol-3(2H)-one* **10f**. The general procedure starting from 2,4-dimethoxyaniline (1.99 g, 13 mmol) at RT was employed, with a 6-h reaction time with separation by column chromatography using CHCl_3_ as an eluent to obtain **10f**. Yield: (0.87 g, 65% calculated on the benzoyl chloride **9**); m.p.: 239–240 °C; as pale yellow prisms (CHCl_3_, 75 mL/g). IR (KBr, cm^−1^) ν 3050, 1601 (C=O), 1588, 1560, 1510, 1442, 1208 (C–N), 1162 (C–O), 1044 (C–O), 747 (C–Se). ^1^H-NMR (300 MHz, CDCl_3_): δ 3.79 (s, 3H, OCH_3_), 3.84 (s, 3H, OCH_3_), 6.54 (dd, *J* = 8.6 Hz, *J* = 2.6 Hz, 1H, Ph-H), 6.59 (d, *J* = 2.6 Hz, 1H, Ph-H), 7.25 (d, *J* = 8.6 Hz, 1H, Ph-H), 7.41 (ddd, *J* = 7.8 Hz, *J* = 7.2 Hz, *J* = 1.2 Hz, 1H, Ar-H), 7.60 (ddd, *J* = 7.8 Hz, *J* = 7.2 Hz, *J* = 1.1 Hz, 1H, Ar-H), 7.95 (dd, *J* = 7.2 Hz, *J* = 1.2 Hz, 1H, Ar-H), 7.99 (dd, *J* = 8.0 Hz, *J* = 1.1 Hz, 1H, Ar-H). ^1^H-NMR (400 MHz, DMSO-*d*_6_): δ 3.75 (s, 3H, OCH_3_), 3.81 (s, 3H, OCH_3_), 6.58 (dd, *J* = 8.6 Hz, *J* = 2.7 Hz, 1H, Ph-H), 6.70 (d, *J* = 2.7 Hz, 1H, Ph-H), 7.23 (d, *J* = 8.6 Hz, 1H, Ph-H), 7.45 (ddd, *J* = 7.6 Hz, *J* = 7.3 Hz, *J* = 1.1 Hz, 1H, Ar-H), 7.65 (ddd, *J* = 7.9 Hz, *J* = 7.3 Hz, *J* = 1.5 Hz, 1H, Ar-H), 7.85 (ddd, *J* = 7.6 Hz, *J* = 1.5 Hz, *J* = 0.6 Hz, 1H, Ar-H), 8.05 (ddd, *J* = 7.9 Hz, *J* = 1.1 Hz, *J* = 0.6 Hz, 1H, Ar-H). ^13^C-NMR (101 MHz, DMSO-*d*_6_): δ 55.45 (OCH_3_), 55.68 (OCH_3_), 99.39, 104.86, 119.75, 125.74, 125.78, 127.30, 127.74, 130.33, 131.77, 140.29, 156.29, 160.09, 165.76 (C=O). HRMS (TOF MS ESI): *m*/*z* for C_15_H_13_NO_3_Se + Na^+^ calculated: 357.9953; found: 357.9961. Anal. Calcd. for C_15_H_13_NO_3_Se (334.2286): C, 53.90; H, 3.92; N, 4.19. Found: C, 53.94; H, 3.75; N, 4.11.

*2-(3,4-Dimethoxyphenyl)-1,2-benzoselenazol-3(2H)-one* **10g** [53]. The general procedure starting from 3,4-dimethoxyaniline (0.61 g, 4.0 mmol) and anhydrous Et_3_N (0.81 g, 1.1 mL, 8 mmol) in anhydrous MeCN (30 mL) at RT was employed, with a 48-h reaction time. Distilled water (60 mL) was added portionwise and left to refrigerator overnight to crystallise. The formed solid was filtered off, washed with water, and dried in the air to obtain **10g**. Yield: (0.91 g, 68%). The analytically pure sample was obtained by column chromatography using CHCl_3_:EtOAc in gradient as an eluent with 40% yield; m.p.: 159–161°C; as colorless crystals (EtOAc). ^1^H-NMR (300 MHz, DMSO-*d*_6_): δ 3.78 (s, 6H, 2OCH_3_), 6.99 (d, *J* = 8.6 Hz, 1H, Ph-H), 7.03 (dd, *J* = 8.6 Hz, *J* = 1.9 Hz, 1H, Ph-H), 7.29 (d, *J* = 1.9 Hz, 1H, Ph-H), 7.47 (dd, *J* = 7.8 Hz, *J* = 7.2 Hz, 1H, Ar-H), 7.67 (dd, *J* = 8.1 Hz, *J* = 7.2 Hz, 1H, Ar-H), 7.90 (d, *J* = 7.8 Hz, 1H, Ar-H), 8.09 (d, *J* = 8.1 Hz, 1H, Ar-H). ^13^C-NMR (75 MHz, DMSO-*d*_6_): 55.5 (OCH_3_), 55.6 (OCH_3_), 109.5 (C–H), 111.8 (C–H), 117.2 (C–H), 125.8 (C–H), 126.1 (C–H), 127.8 (C–H), 128.4 (C), 132.0 (C–H), 132.4 (C), 139.0 (C), 147.1 (C), 148.7 (C), 164.9 (C=O). HRMS (TOF MS ESI): *m*/*z* for C_15_H_13_NO_3_Se + Na^+^ calculated: 357.9953; found: 357.9951. Anal. Calcd. for C_15_H_13_NO_3_Se (334.2282): C, 53.90; H, 3.92; N, 4.19. Found: C, 53.79; H, 4.05; N, 4.08.

### 3.2. Evaluation of Antimicrobial Activity

The antimicrobial activity of compounds **4**–**6**, **7**, **10** was determined by the 96-well microdilution assay, according to Clinical and Laboratory Standards Institute (2008), 3rd ed. M27-A3 [73] with slight modifications, using Gram-positive bacterial strains: *Staphylococcus aureus* ATCC 6538, *Staphylococcus epidermidis* ATCC 14990, *Enterococcus faecalis* ATCC 29212, *Enterococcus hirae* ATCC 10541; Gram-negative bacterial strains: *Escherichia coli* ATCC 10536, *Pseudomonas aeruginosa* ATCC 15442; yeast-like fungi: *Candida albicans* ATCC 10231, *Candida glabrata* ATCC 90030; and filamentous fungus *Aspergillus niger.* Standard strains were supplied by American Type Culture Collection (ATCC), while *A. niger* was obtained from the Collection of Microorganisms of Wroclaw Medical University. Bacterial strains were cultured in Lysogeny broth (LB) at 37 °C and yeast strains in Sabouraud dextrose broth at 30 °C.

Briefly, stock solutions in DMSO were serially diluted in LB or Sabouraud dextrose broth to obtain a final range of 0.125–128 µg/mL concentration in microdilution sterile plates (Sarstedt, Stare Babice, Poland). Microbial inocula were prepared to obtain a final optical density at λ = 600 nm (OD600) = 0.1 ± 10% in a well. The plates were cultivated for 24 h at 37 °C (bacteria) or 48 h at 30 °C (fungi). Afterwards, OD600 was measured using a microplate reader ASYS UVM 340 (Biogenet, Józefów, Poland). The concentration which resulted in ≥90% growth inhibition was determined as the minimal inhibitory concentration (MIC_90_; μg/mL).

### 3.3. Evaluation of Antiviral Activity and Cytotoxicity

The compounds **4**–**5**, **7**, **10** at various concentrations were incubated with the following viruses: HHV-1 (human herpes virus type 1), EMCV (encephalomyocarditis virus), and VSV (vesicular stomatitis virus) for 1 h at RT. HHV-1 was used at the dose of 10^6^ TCID_50_/mL, whereas EMCV and VSV were used at the dose of 10^8^ TCID_50_/mL. The TCID_50_ (tissue culture infectious dose) determines the cytopathic effect caused by the virus in about 50% of infected cells. The virus titer was measured in human cell line A549 (human lung adenocarcinoma cell line, ATCC 185) which was observed for cytopathic effect after 48 h. The minimal concentration that caused 1000-fold decrease of virus titer was taken as the minimal inhibitory concentration, MIC (μg/mL).

In parallel, the cytotoxicity of the compounds was determined in the A549 cell line (cell line used in antiviral activity test) by incubation at various concentrations for 48 h at 37 °C in the atmosphere of 5% CO_2_ in air. The minimal concentration which was toxic to approximately 50% of the cells was taken as the TCCD_50_ (Tissue Culture Cytotoxic Dose).

Both, cytotoxicity and virus titers were determined using MTT assay. To ensure that colourful compounds did not interfere with MTT reagents, the extent of cell damage was evaluated by microscopic examination prior to MTT assay.

## 4. Conclusions

The reaction of bis[(2-chlorocarbonyl)phenyl] diselenide **3** with various aminophenols and their derivatives is a convenient synthetic route to bis[2-(hydroxyphenylcarbamoyl)]phenyl diselenides **4** (reduced forms of ebselenols) and other compounds containing protected hydroxyl groups. This general approach allows the production of ebselenol-derived diselenides with higher yields than the alternative method based on the reductive benzisoselenazolone ring opening of ebselenols. The secondary amine such as morpholine and benzohydrazide also reacted on their nitrogen atoms to form bis[2-(*N*-morpholinecarbamoyl)]phenyl and bis[2-(*N*-benzamidecarbamoyl)]phenyl diselenides, respectively, while reaction with aminophenol derivatives containing a blocked amine group such a 2-hydroxyacetanilide and natural antimycin A led to corresponding phenolic esters of bis(2-carboxy)phenyl diselenide. As a result of our studies, 15 new structures have been developed, including 13 diselenides and two benzisoselenazol-3(2*H*)-ones.

The investigation of the antimicrobial and antiviral activities of bis[2-(hydroxyphenylcarbamoyl)]phenyl diselenides and their derivatives as well as cyclic analogues have revealed new biologically active structures. The compounds, particularly bis[2-(hydroxyphenylcarbamoyl)]phenyl diselenides and reference benzisoselenazol-3(2*H*)-ones, exhibited high antimicrobial activity against Gram-positive bacterial species (*Enterococcus* spp., *Staphylococcus* spp.), and some compounds were also active against Gram-negative *E. coli* and fungi (*Candida* spp., *A. niger*). The antiviral activity evaluation allowed the identification of substances with high anti-HHV-1 and moderate anti-EMCV activities. The chemotherapeutic indices for some of these compounds were significantly higher (concentration active against virus was lower than the one showing a cytotoxic effect) than for other organoselenium compounds previously reported in the literature.

As novel molecules with the ability to inhibit microbial and viral functions are in high demand, the results of our studies are encouraging for further exploration of organoselenium compounds as anti-infective agents.

## Figures and Tables

**Table 1:** Characteristics of compounds **4a**–**p**, **5**–**8**, and **10a**–**g**.

Compound	R^1^	R^2^	R^3^	X	Rx Time (h)	Yield (%)	m.p. (°C)	m.p. lit (°C)
**4a**	2-OH	-	-	-	96	92	233.5–236.5	235–238 [53]
**4b**	3-OH	-	-	-	60	84	285.5–287.0	285–286 [53]
**4c**	4-OH	-	-	-	120	73	271.0–272.0	282–284 [53]
**4d**	2-OH	4-Me	-	-	24	60	221.0–223.5	-
**4e**	2-OH	5-Me	-	-	96	89	191.5–194.5	-
**4f**	2-OH	6-Me	-	-	168	88	264.0–267.0	-
**4g**	2-OH	4-Cl	-	-	96	59	230.0–234.0	-
**4h**	2-OH	5-Cl	-	-	48	63	219.5–221.5	-
**4i**	2-OH	5-COOMe	-	-	72	57	281.0–282.0	-
**4j**	3-OH	4-OMe	-	-	120	94	232.5–236.5	-
**4k**	2-OMe	-	-	-	168	94	211.0–214.0	216–217 [53]
**4l**	3-OMe	-	-	-	72	98	221.0–223.0	221–223 [35]
**4m**	4-OMe	-	-	-	48	94	285.5–287.5	290–292 [53]
**4n**	2-OMe	4-Ome	-	-	168	99	211.0–213.5	-
**4o**	3-OMe	4-OMe	-	-	96	72	231.5–234.5	235 [53]
**4p**	3-OMe	4-OMe	5-OMe	-	96	76	193.0–195.0	-
**5**	-	-	-	-	120	85	149.0–152.0	-
**6**	-	-	-	-	120	100	296.5 dec.	-
**7**	-	-	-	-	96	36	178.0–181.0	-
**8**	-	-	-	-	240	97	>300	-
**10a**	2-OH	-	-	CH	20	40	195.5–197.5	194–196 [54]
**10b**	6-OH	-	-	N	2	65	229.0 dec.	199–201 [55] ^a^
**10c**	2-OMe	-	-	CH	24	71	189.0–191.5	172–174 [63]
**10d**	3-OMe	-	-	CH	2	65	166.0–168.0	165.5–167.5 [35]
**10e**	4-OMe	-	-	CH	20	70	180.0–181.0	180.5–181.5 [35]
**10f**	2-OMe	4-Ome	-	CH	6	65	240.5–242.5	-
**10g**	3-OMe	4-Ome	-	CH	48	68	159.0–161.0	-

^a^ Phase transfer was observed at 199 °C.

**Table 2:** Antibacterial activity of compounds **4**–**7**, and **10**.

Compound	Gram-Positive Bacteria ^a^				Gram-Negative Bacteria ^a^	
	*S. aureus*	*S. epidermidis*	*E. faecalis*	*E. hirae*	*E. coli*	*P. aeruginosa*
**4a**	16	8	4	8	>128	>128
**4b**	16	8	2	8	>128	>128
**4c**	16	16	2	8	>128	>128
**4d**	>128	128	2	4	>128	>128
**4e**	16	16	1	2	64	>128
**4f**	>128	>128	4	8	>128	>128
**4g**	128	64	2	4	>128	>128
**4h**	>128	128	>128	>128	>128	>128
**4i**	>128	128	>128	>128	>128	>128
**4j**	>128	128	>128	>128	>128	>128
**4k**	>128	>128	>128	>128	>128	>128
**4l**	>128	128	>128	>128	>128	>128
**4m**	>128	>128	8	>128	>128	>128
**4n**	>128	128	4	64	>128	>128
**4o**	>128	128	16	64	>128	>128
**4p**	>128	128	4	32	>128	>128
**5**	>128	128	>128	>128	>128	>128
**6**	>128	128	4	8	>128	>128
**7**	>128	>128	>128	>128	>128	>128
**10a**	8	4	2	4	32	>128
**10b**	8	4	1	2	>128	>128
**10c**	32	32	2	8	128	>128
**10d**	>128	128	16	32	>128	>128
**10e**	>128	128	4	32	>128	>128
**10f**	64	64	2	4	>128	>128
**10g**	64	32	2	8	>128	>128
Ebs	64	32	2	8	128	>128
AMC	>128	>128	>128	>128	>128	>128
AMP	4	>128	16	>128	16	>128
CHK	>128	>128	128	128	2	>128
ERM	>128	>128	>128	>128	64	>128
STR	32	128	>128	>128	32	128
NAT	>128	>128	>128	>128	16	32

^a^ MIC—Minimum Inhibitory Concentration (µg/mL); AMC—antimycin A; AMP—ampicillin; CHK—chloramphenicol; ERM—erythromycin; STR—streptomycin; NAT—nourseothricin.

**Table 3:** Antifungal activity of compounds **4**–**7**, and **10**.

Compound	Yeasts ^a^		Fungi ^a^
	*C. albicans*	*C. glabrata*	*A. niger*
**4a**	>128	>128	>128
**4b**	>128	>128	>128
**4c**	>128	>128	>128
**4d**	>128	>128	>128
**4e**	32	32	128
**4f**	>128	>128	>128
**4g**	>128	>128	>128
**4h**	>128	>128	>128
**4i**	>128	>128	>128
**4j**	>128	>128	>128
**4k**	>128	>128	>128
**4l**	>128	>128	>128
**4m**	>128	>128	>128
**4n**	>128	>128	>128
**4o**	>128	>128	>128
**4p**	>128	>128	>128
**5**	>128	>128	-
**6**	>128	>128	-
**7**	>128	>128	-
**10a**	16	16	>128
**10b**	>128	>128	>128
**10c**	16	32	64
**10d**	>128	>128	-
**10e**	64	>128	-
**10f**	16	64	64
**10g**	32	64	-
Ebselen	32	64	16
AMC	>128	>128	>128
KLK	>128	>128	-
ITC	>128	>128	-
NAT	64	64	-
AMB	1	1	-

^a^ MIC—Minimum Inhibitory Concentration (µg/mL); AMC—antimycin A; KLK—ketoconazole; ITC—itraconazole; NAT—nourseothricin; AMB—amphotericin B.

**Table 4:** Antiviral activity and cytotoxicity of compounds **4**, **5**, **7**, and **10**.

Compound	TCCD_50_^a^	HHV-1		EMCV		VSV	
		MIC ^b^	I ^c^	MIC ^b^	I ^c^	MIC ^b^	I ^c^
**4a**	4.9	40	0.12	10	0.49	>1000	<0.005
**4c**	3.7	2	1.8	60	0.06	>1000	<0.004
**4d**	4.9	2	2.5	>1000	<0.005	>1000	<0.005
**4e**	4.9	2	2.5	6	0.82	>1000	<0.005
**4f**	9.8	4	2.5	>1000	<0.01	>1000	<0.01
**4g**	4.9	2	2.5	40	0.12	>1000	<0.005
**4h**	4.9	6	0.82	>1000	<0.005	>1000	<0.005
**4i**	7.3	20	0.37	20	0.37	>1000	<0.07
**4j**	4.9	4	1.2	20	0.25	>1000	<0.005
**4k**	312	6	52	400	0.78	>1000	<0.31
**4n**	58	2	29	600	0.10	>1000	<0.06
**4o**	4.9	2	2.5	8	0.61	>1000	<0.005
**4p**	2.4	4	0.61	8	0.30	200	0.01
**5**	4.9	200	0.02	10	0.49	>1000	<0.005
**7**	15	20	0.75	400	0.04	600	0.03
**10c**	9.8	4	2.5	8	1.2	>1000	<0.01
**10f**	156	2	78	200	0.78	>1000	<0.16
Ebselen	15	2	7.5	10	1.5	>1000	<0.02

^a^ TCCD_50_—Tissue Culture Cytotoxic Dose (µg/mL); ^b^ MIC—Minimum Inhibitory Concentration (µg/mL); ^c^ I = TCCD_50_/MIC.

## Supplementary Materials

Supplementary materials are available online.
