# Supplementary material for: Reaction of bis[(2-chlorocarbonyl)phenyl] Diselenide with Phenols, Aminophenols, and Other Amines towards Diphenyl Diselenides with Antimicrobial and Antiviral Properties
Source: Molecules. 2017 Jun 12;22(6):974. doi: 10.3390/molecules22060974 (PMC6152648; doi:10.3390/molecules22060974)
Supplement: Supplementary file 1 [file molecules-22-00974-s001.pdf]

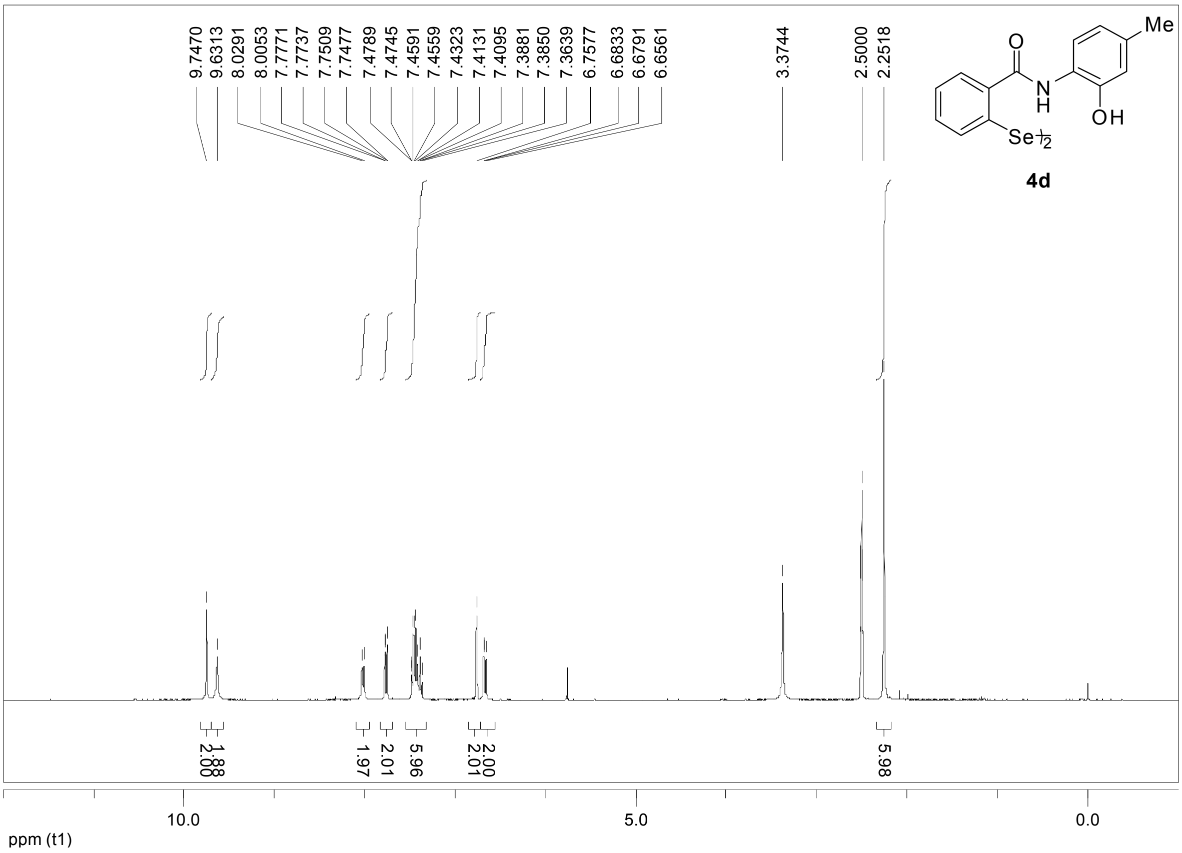

Figure S1. ^{1}H-NMR (DMSO-d$_6$, 300.13 MHz) spectrum of compound **4d**.

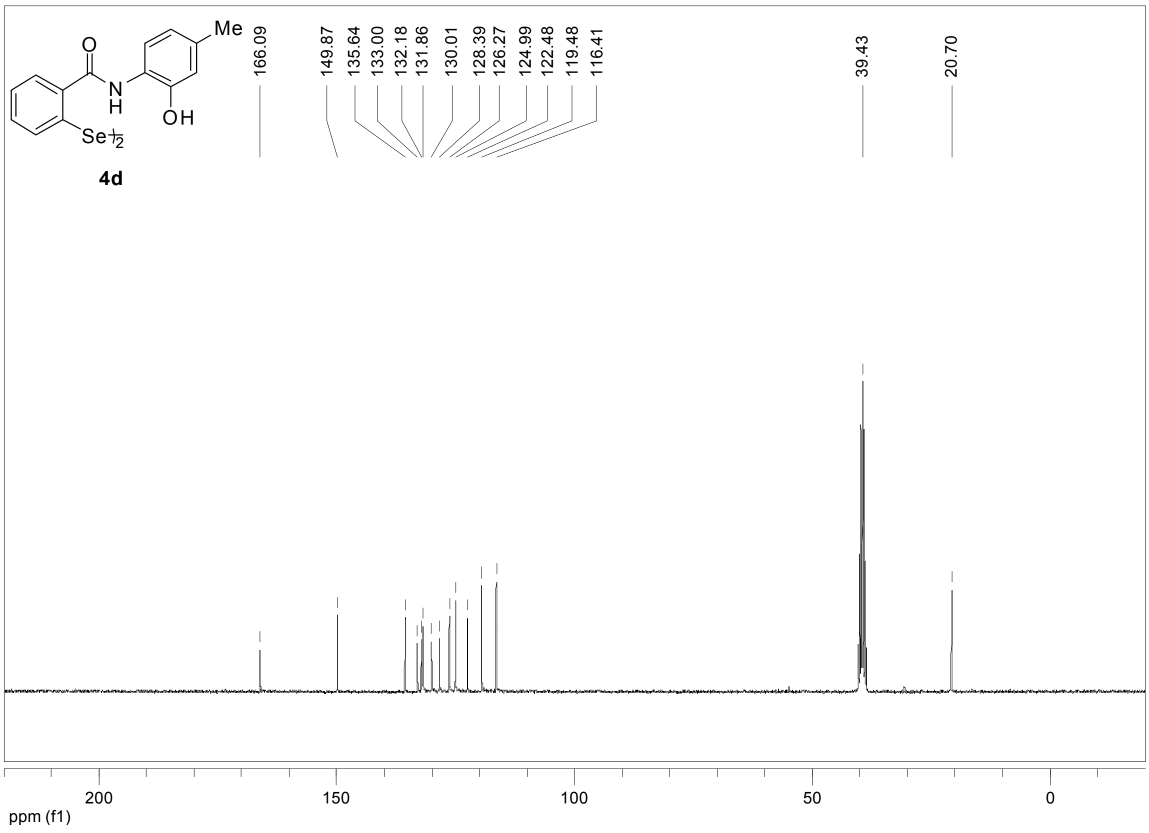

Figure S2. ^{13}C-NMR (DMSO-d$_6$, 75.5 MHz) spectrum of compound **4d**.

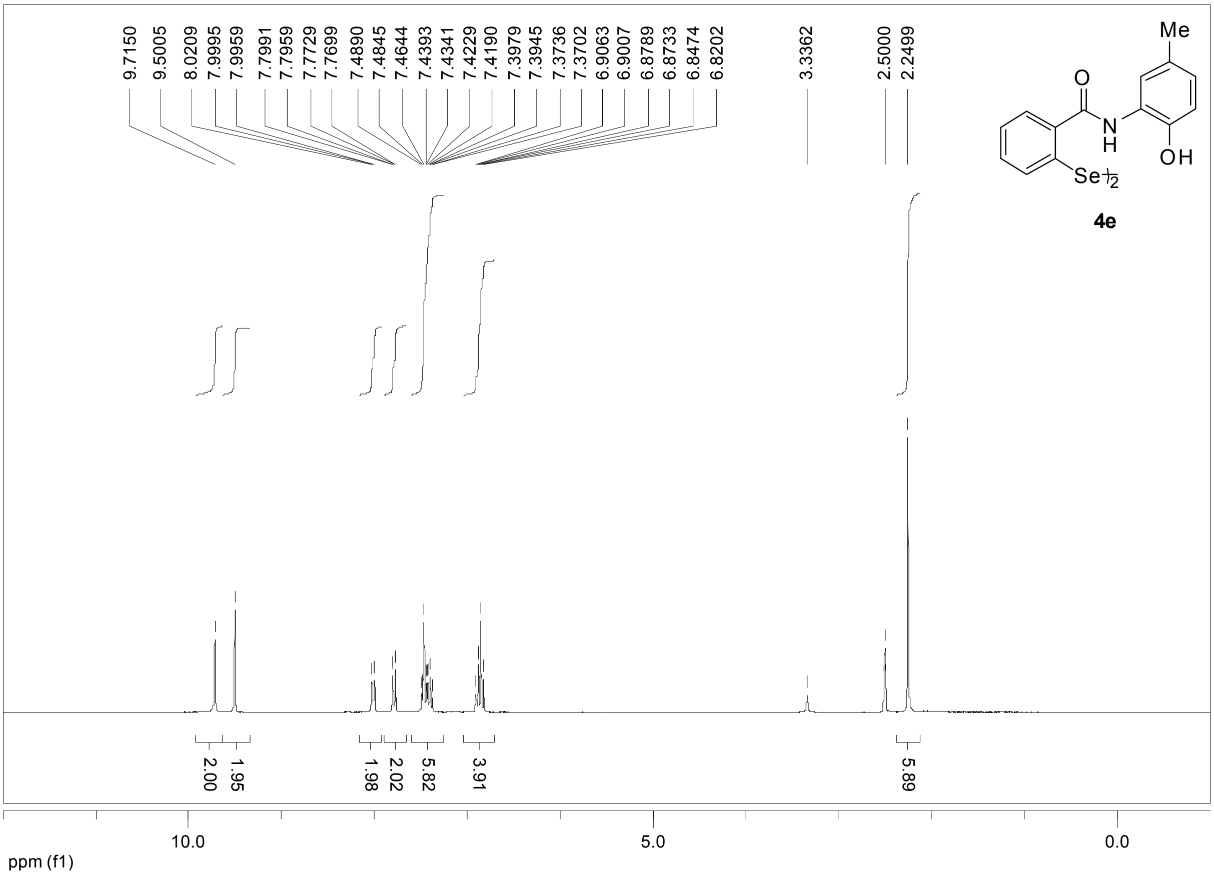

Figure S3. ^{1}H-NMR (DMSO-d$_6$, 300.13 MHz) spectrum of compound **4e**.

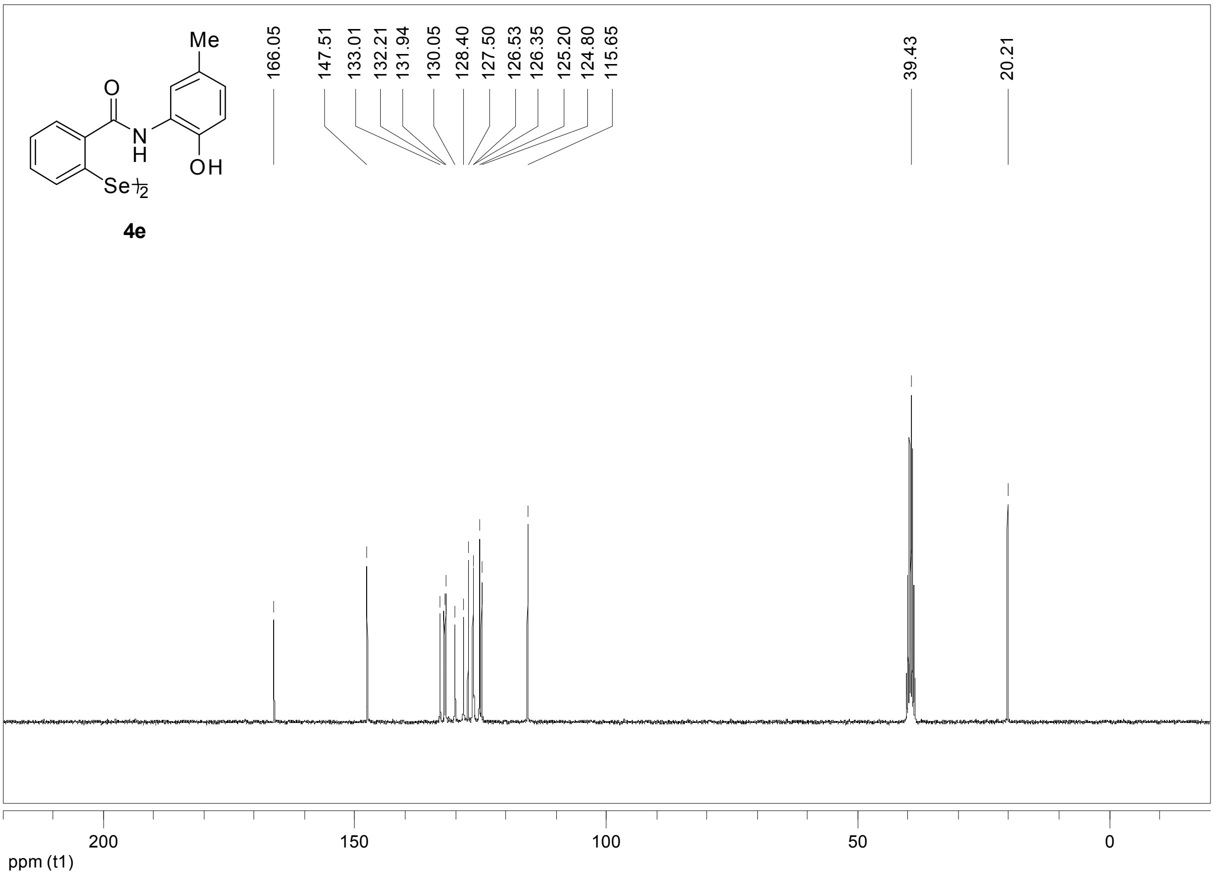

Figure S4. ^{13}C-NMR (DMSO-d$_6$, 75.5 MHz) spectrum of compound **4e.**

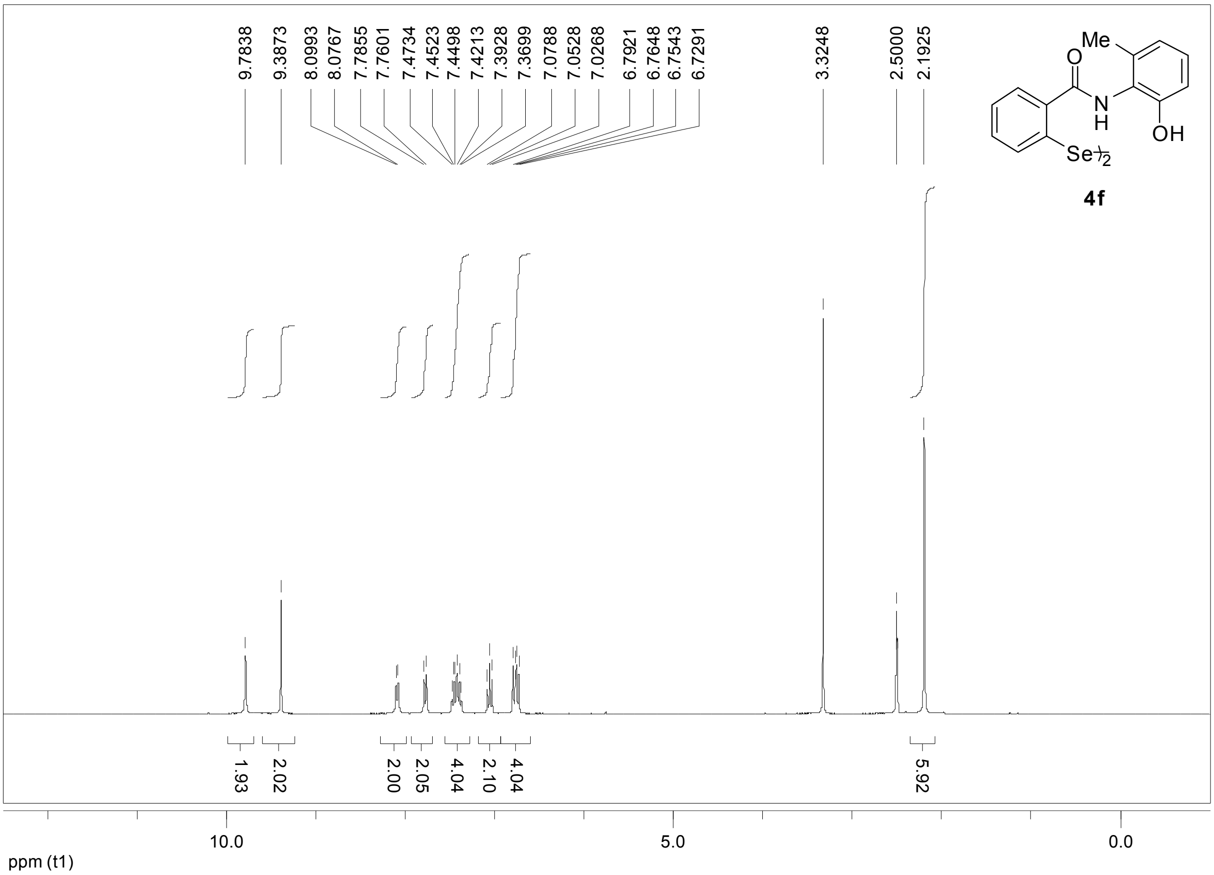

Figure S5. ^{1}H-NMR (DMSO-d$_6$, 300.13 MHz) spectrum of compound **4f.**

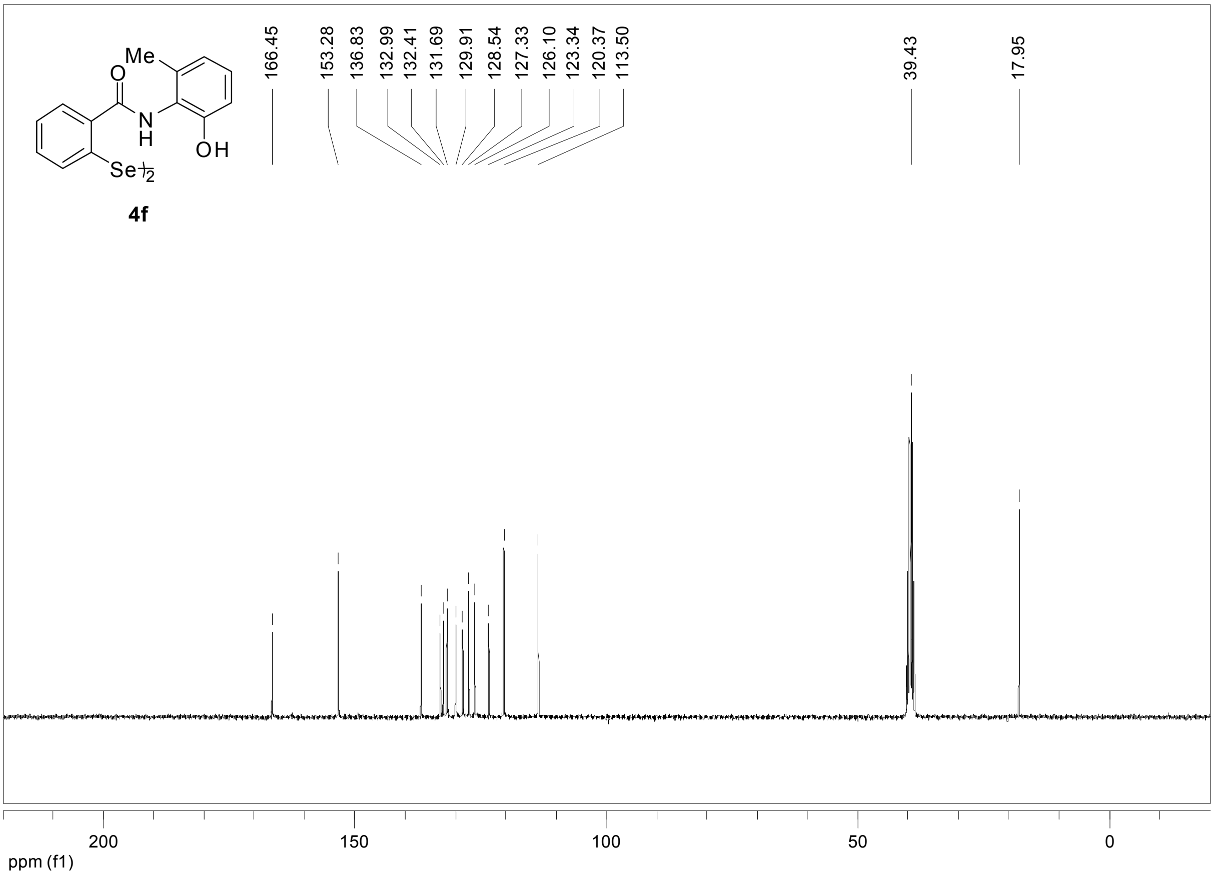

Figure S6. ^{13}C-NMR (DMSO-d$_6$, 75.5 MHz) spectrum of compound **4f.**

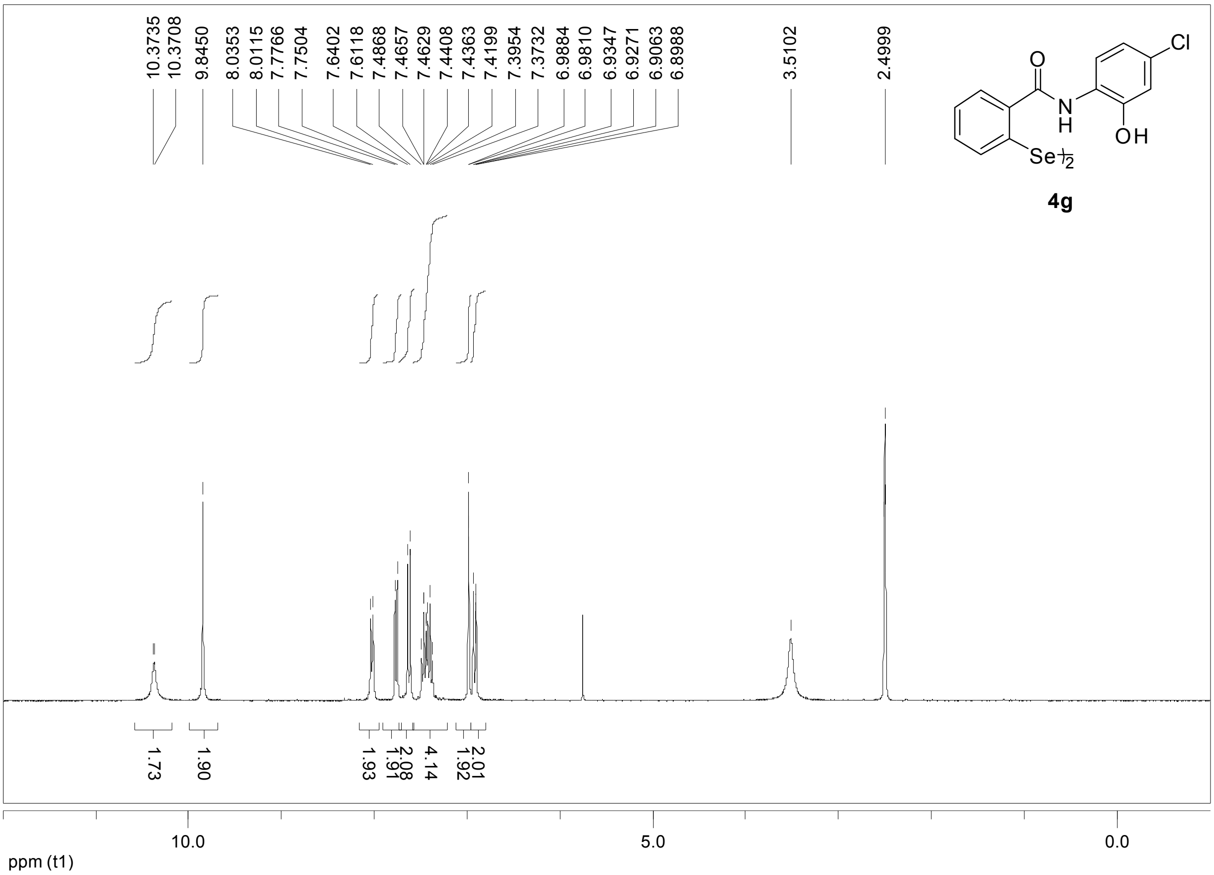

Figure S7. ^{1}H-NMR (DMSO-d$_6$, 300.13 MHz) spectrum of compound **4g.**

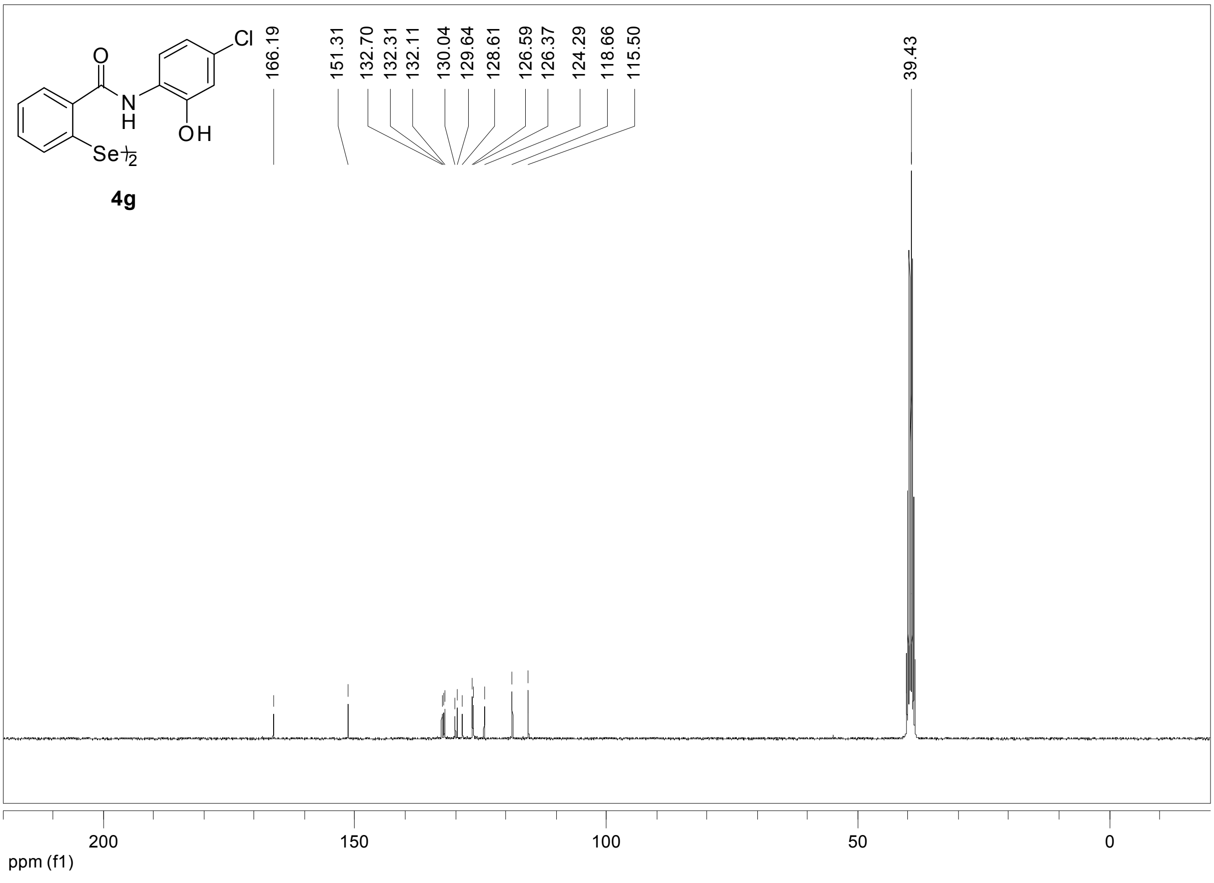

Figure S8. ^{13}C-NMR (DMSO-d$_6$, 75.5 MHz) spectrum of compound **4g.**

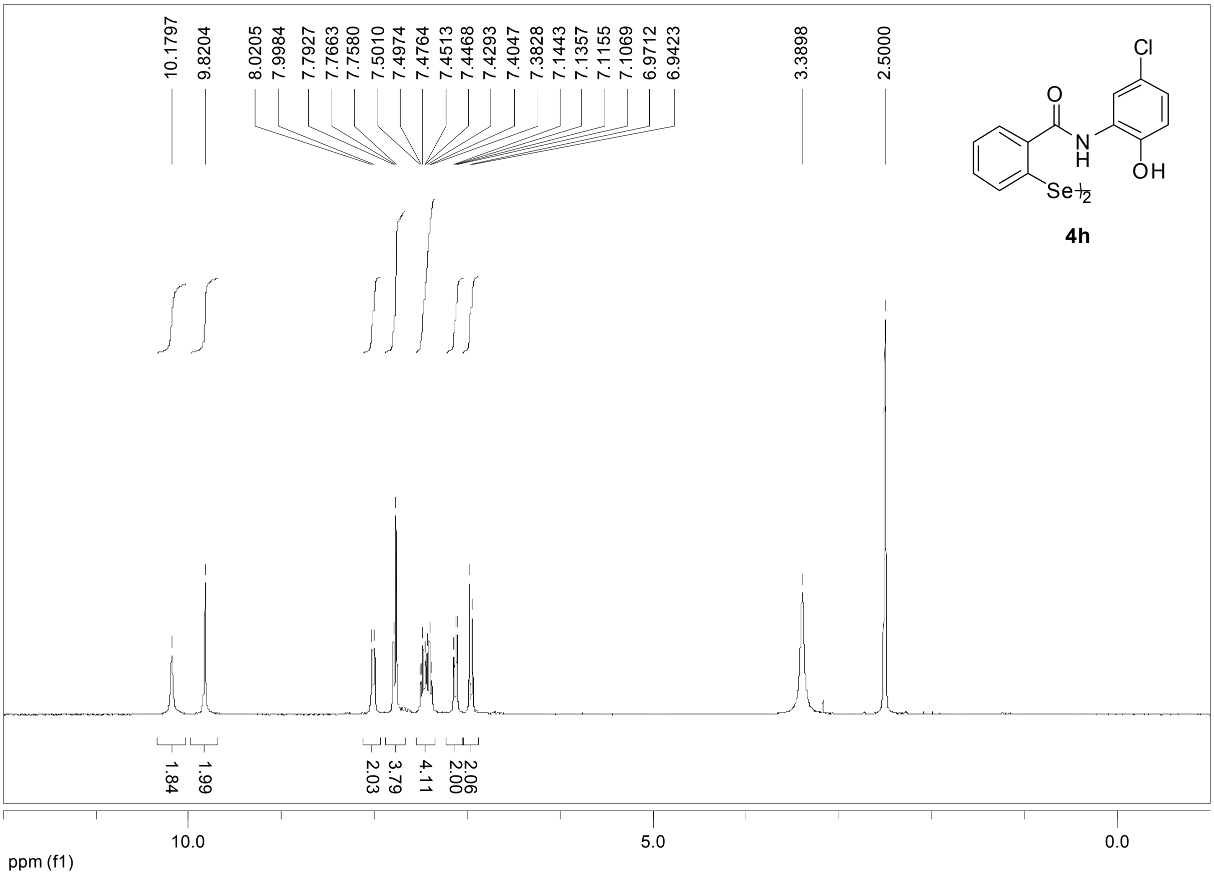

Figure S9. ^{1}H-NMR (DMSO-d$_6$, 300.13 MHz) spectrum of compound **4h.**

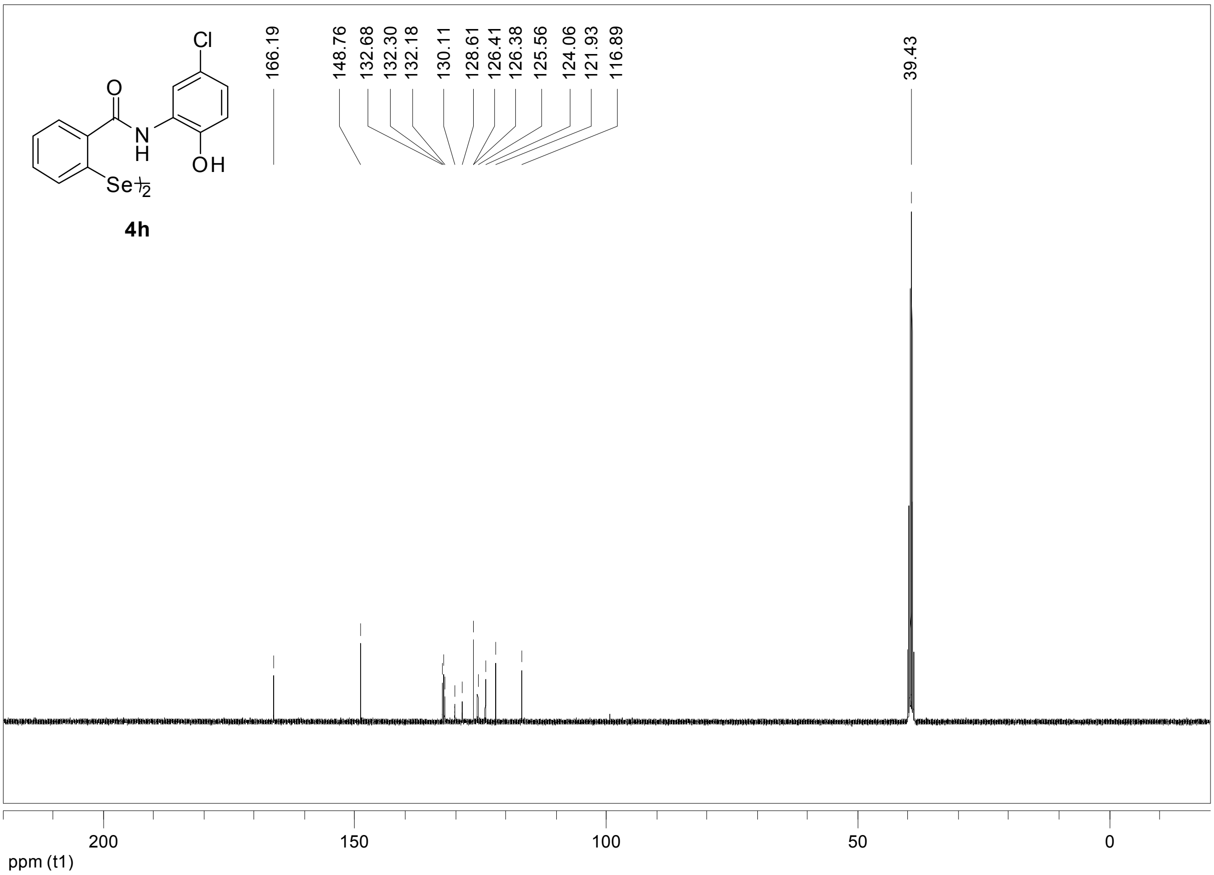

Figure S10. ^{13}C-NMR (DMSO-d$_6$, 100.5 MHz) spectrum of compound **4h.**

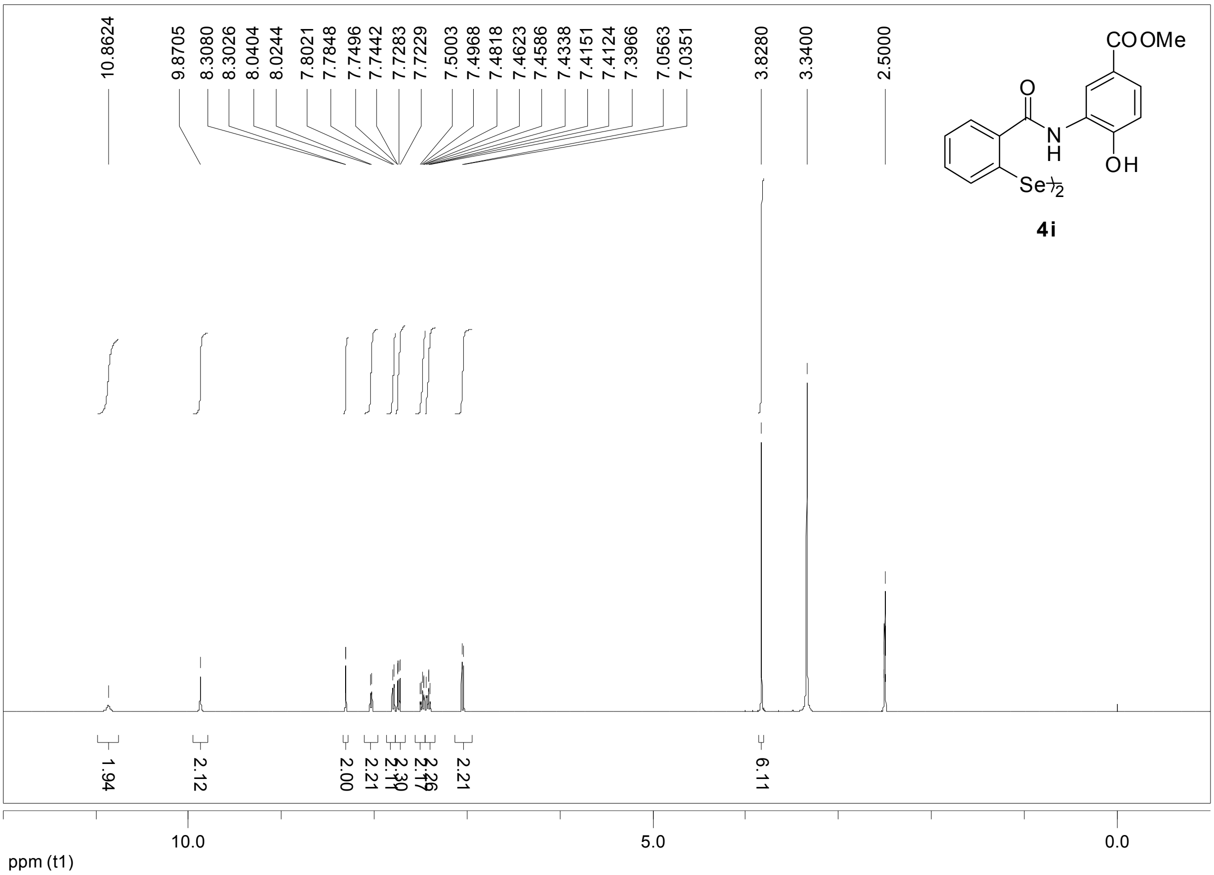

Figure S11. ^{1}H-NMR (DMSO-d$_6$, 399.8 MHz) spectrum of compound **4i.**

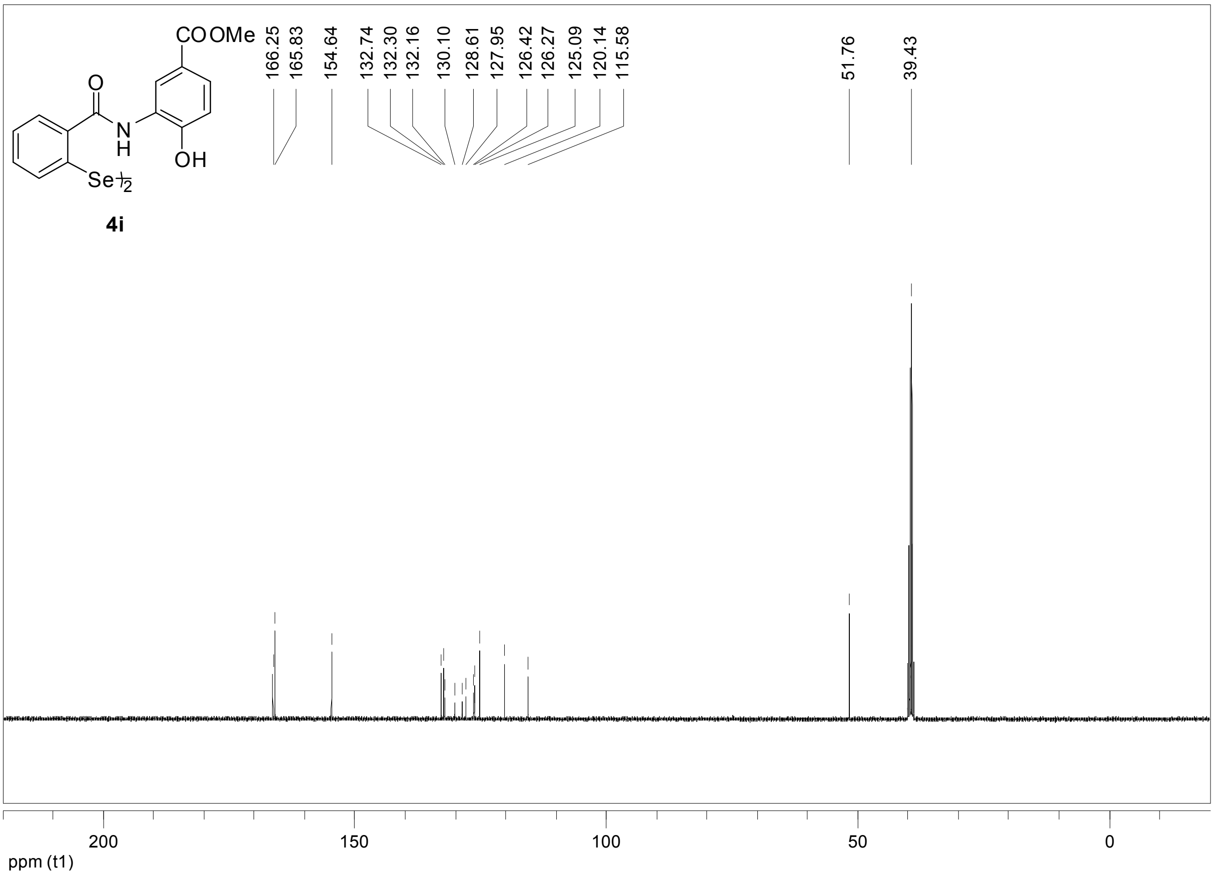

Figure S12. ^{13}C-NMR (DMSO-d$_6$, 100.5 MHz) spectrum of compound **4i.**

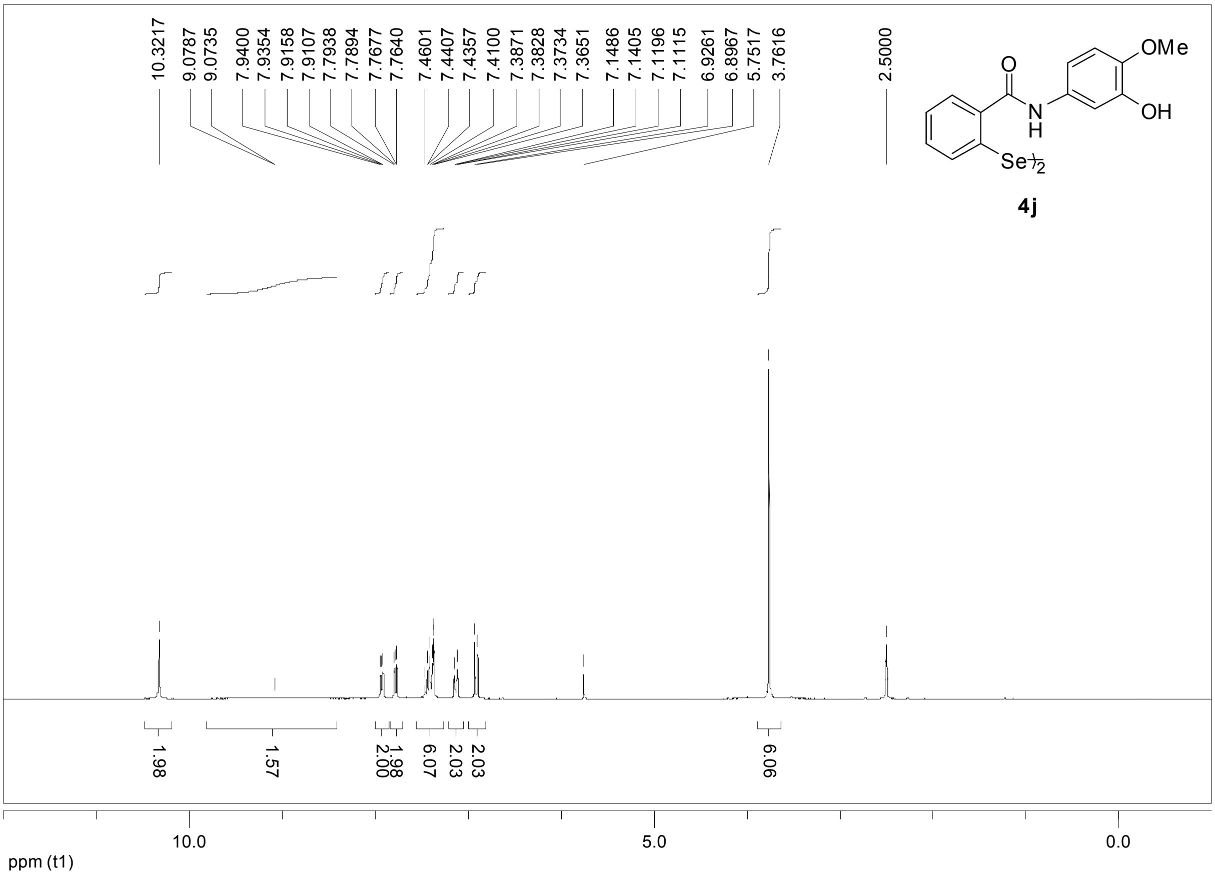

Figure S13. ^{1}H-NMR (DMSO-d$_6$, 300.1 MHz) spectrum of compound **4j.**

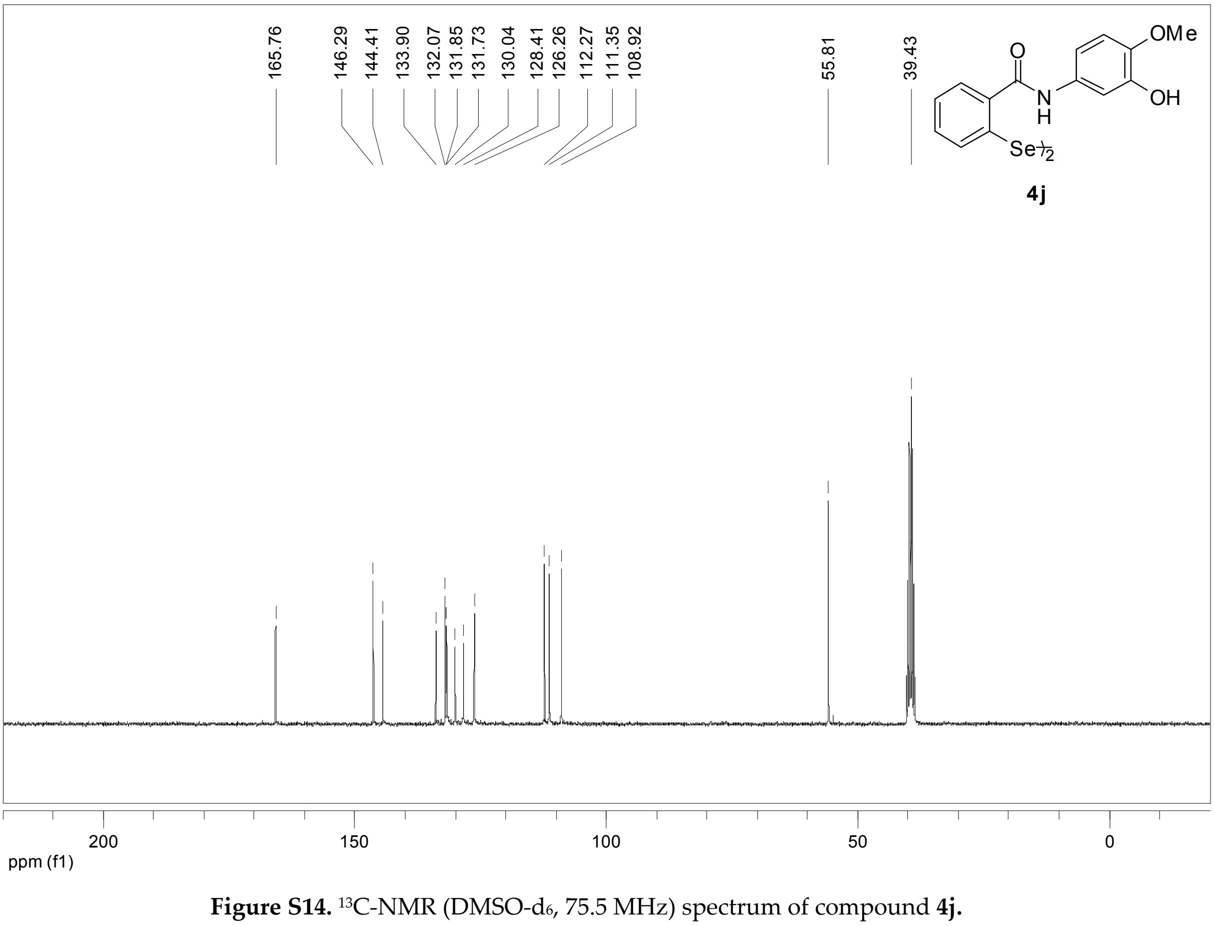

Figure S14. ^{13}C-NMR (DMSO-d$_6$, 75.5 MHz) spectrum of compound **4j.**

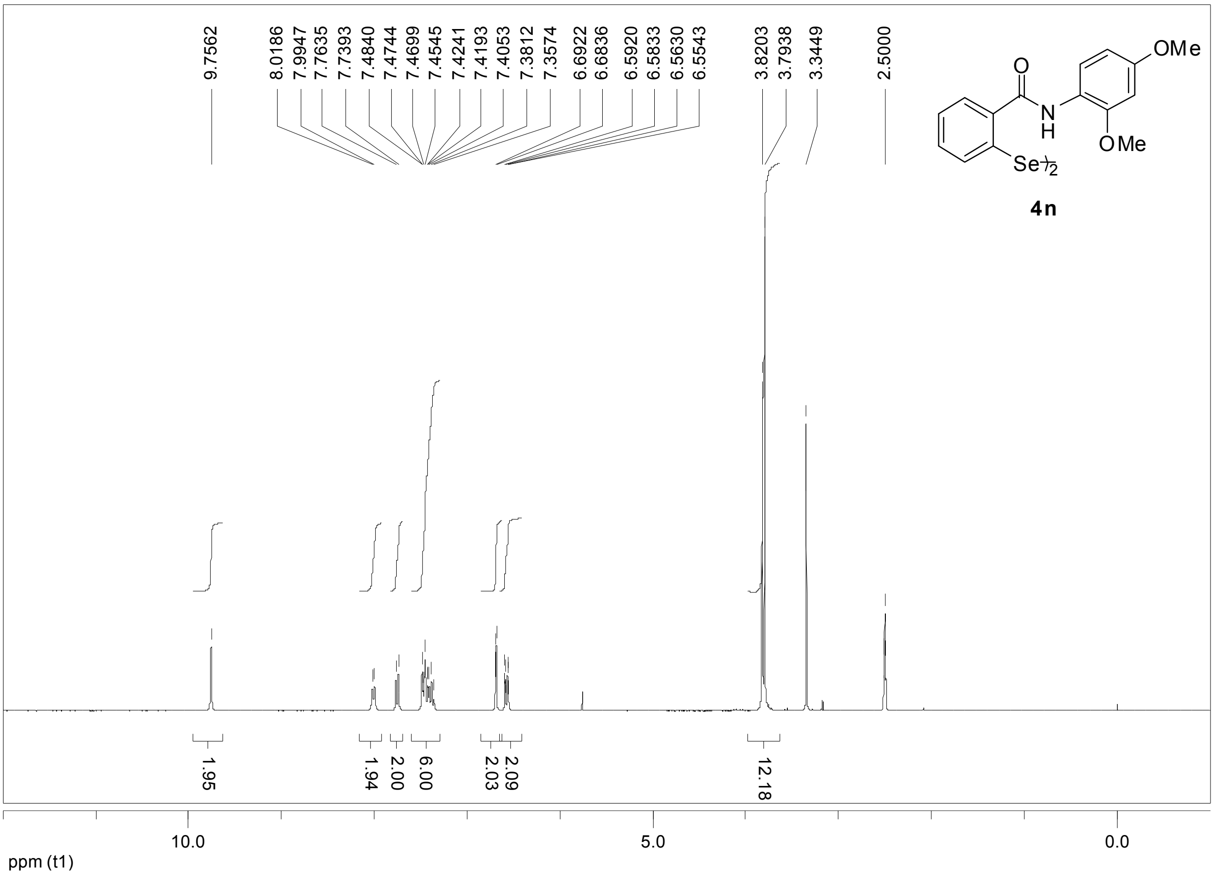

Figure S15. ^{1}H-NMR (DMSO-d$_6$, 300.1 MHz) spectrum of compound **4n.**

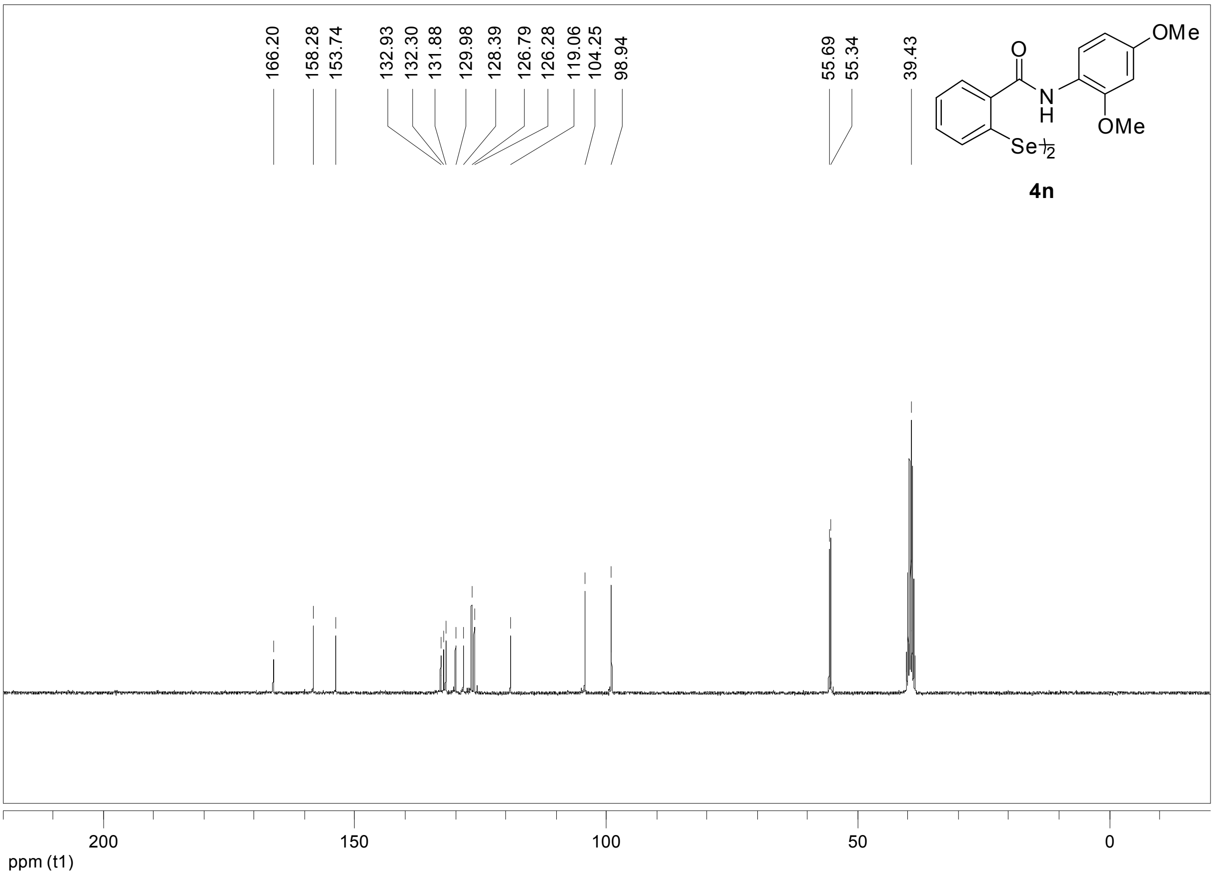

Figure S16. ^{13}C-NMR (DMSO-d$_6$, 75.5 MHz) spectrum of compound **4n.**

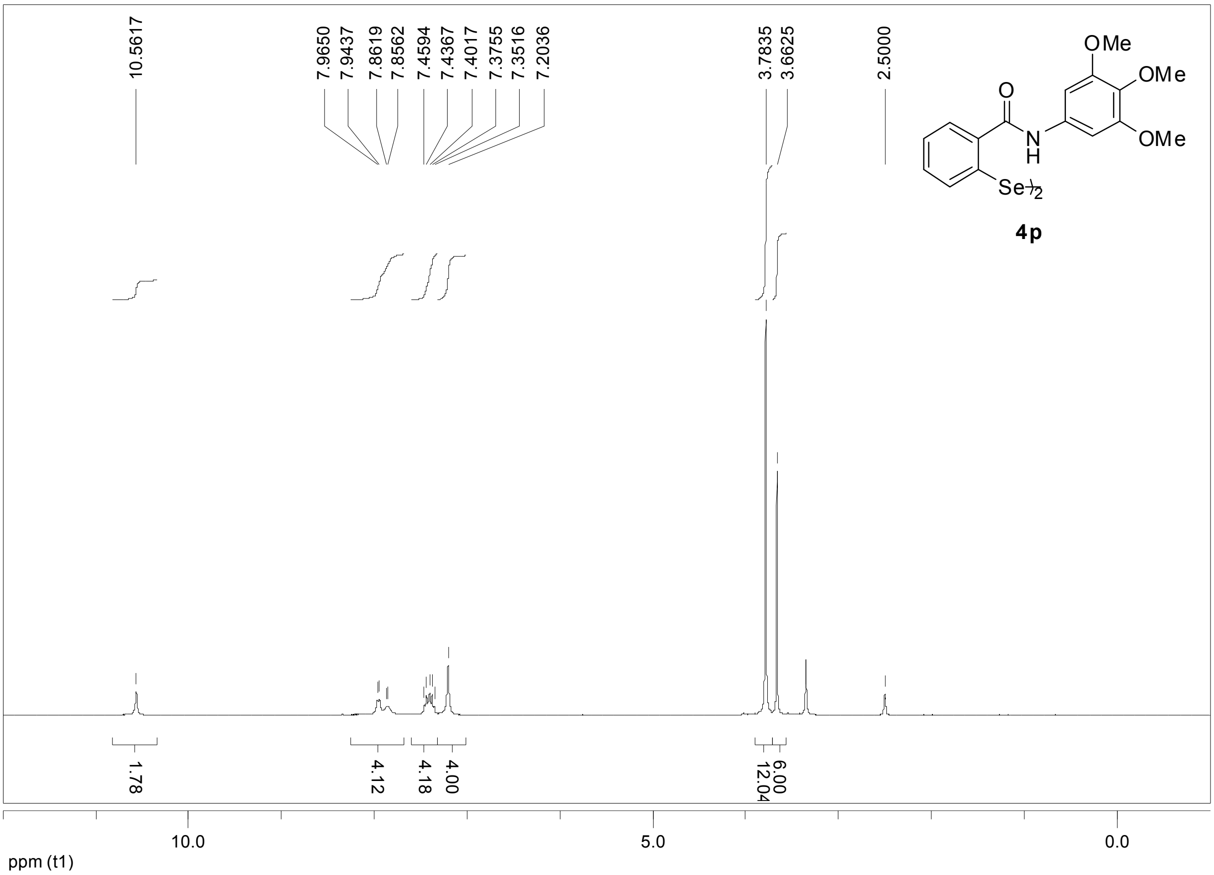

Figure S17. ^{1}H-NMR (DMSO-d6, 300.1 MHz) spectrum of compound **4p.**

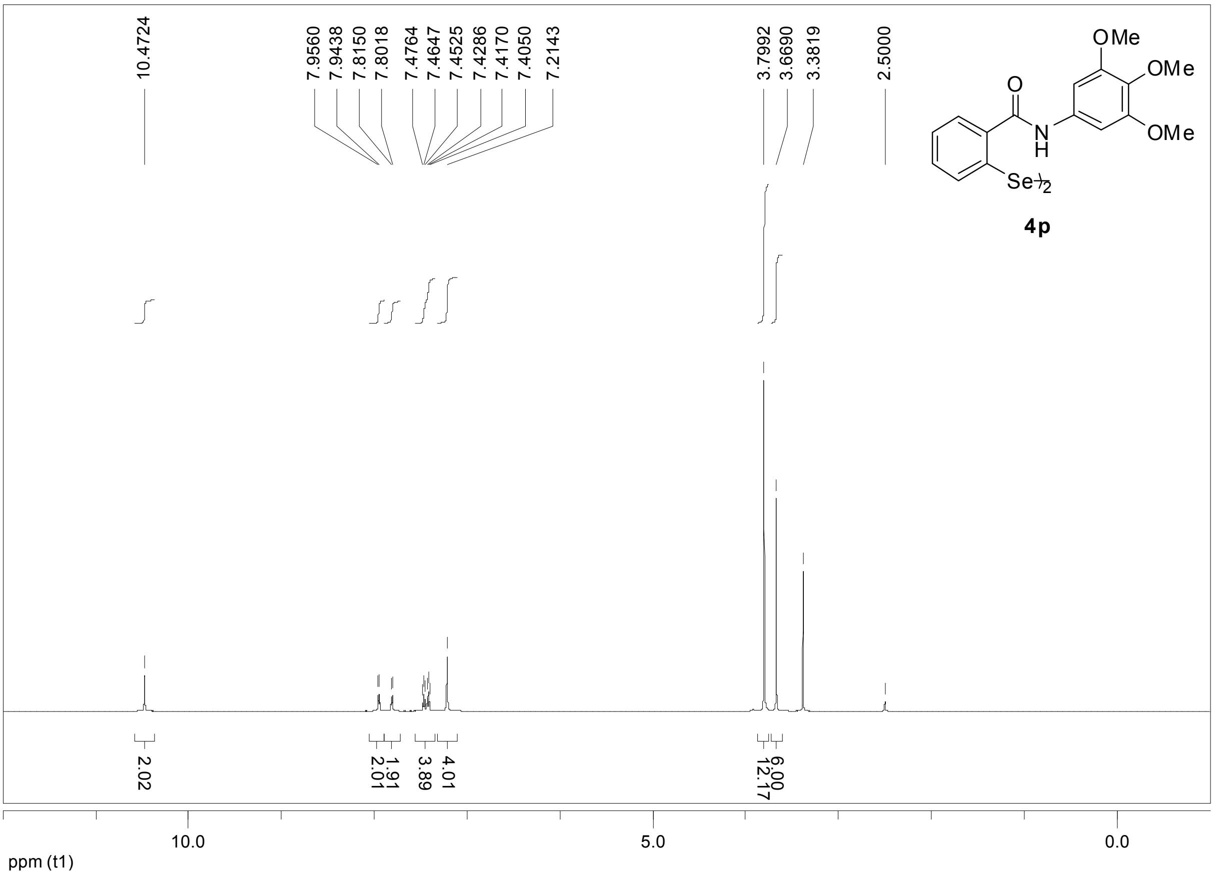

Figure S18. ^{1}H-NMR (DMSO-d6, 600.6 MHz) spectrum of compound **4p.**

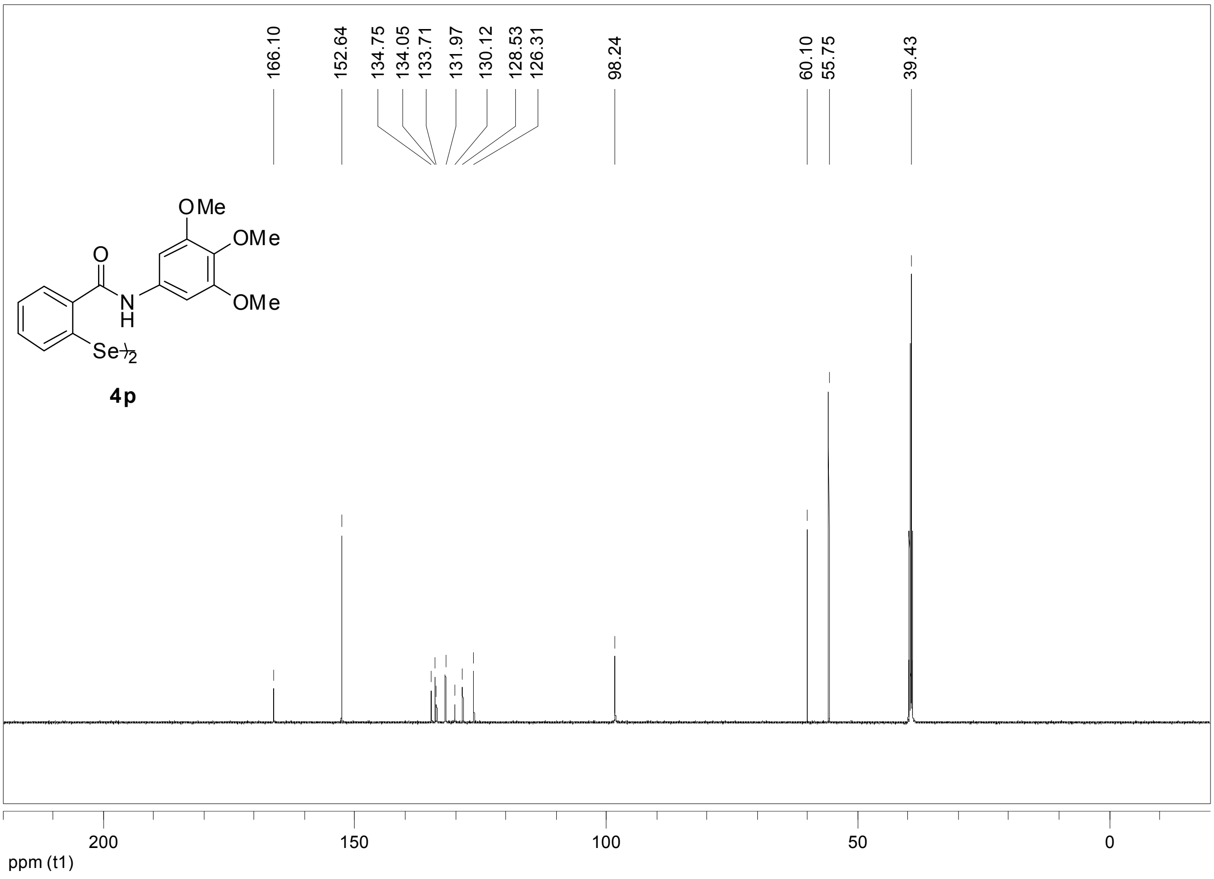

Figure S19. ^{13}C-NMR (DMSO-d_6, 151.0 MHz) spectrum of compound **4p.**

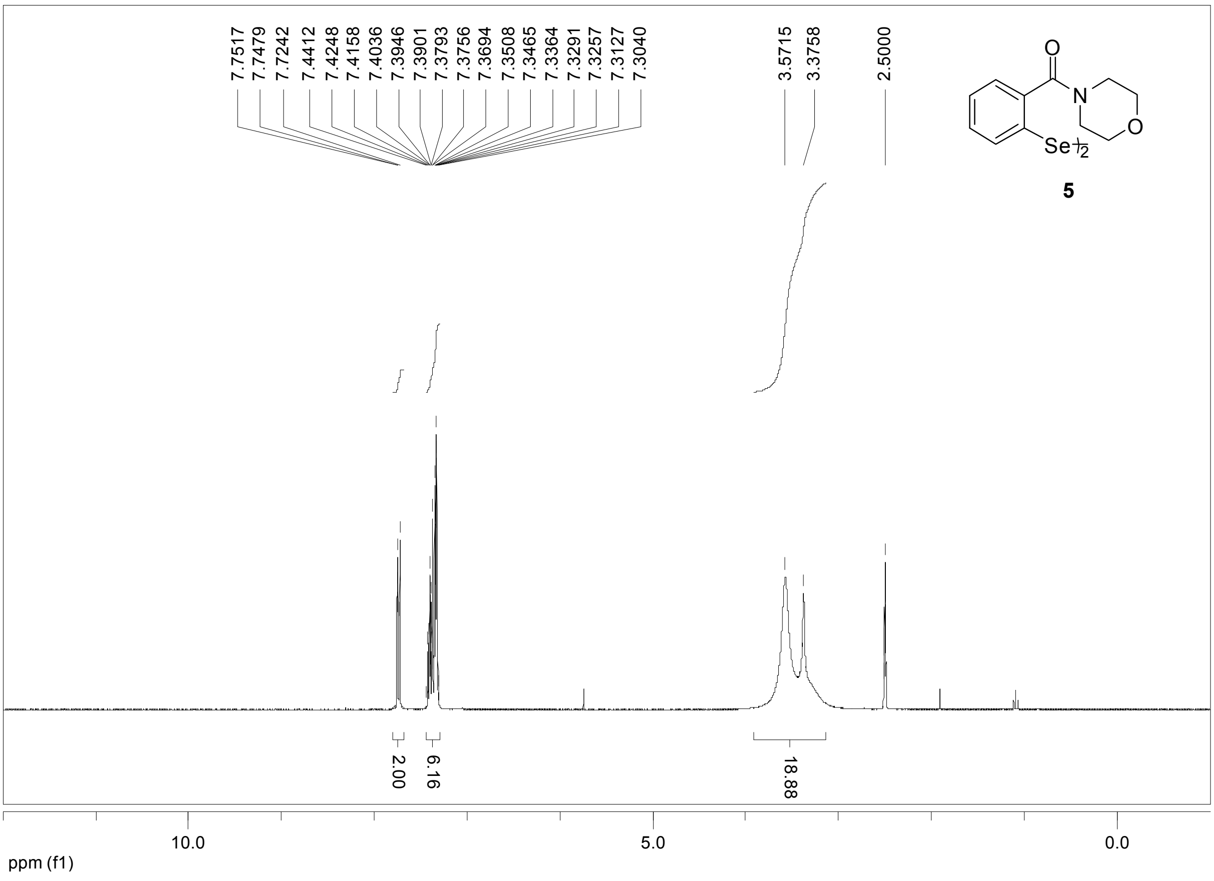

Figure S20. ^{1}H-NMR (DMSO-d_6, 300.1 MHz) spectrum of compound **5.**

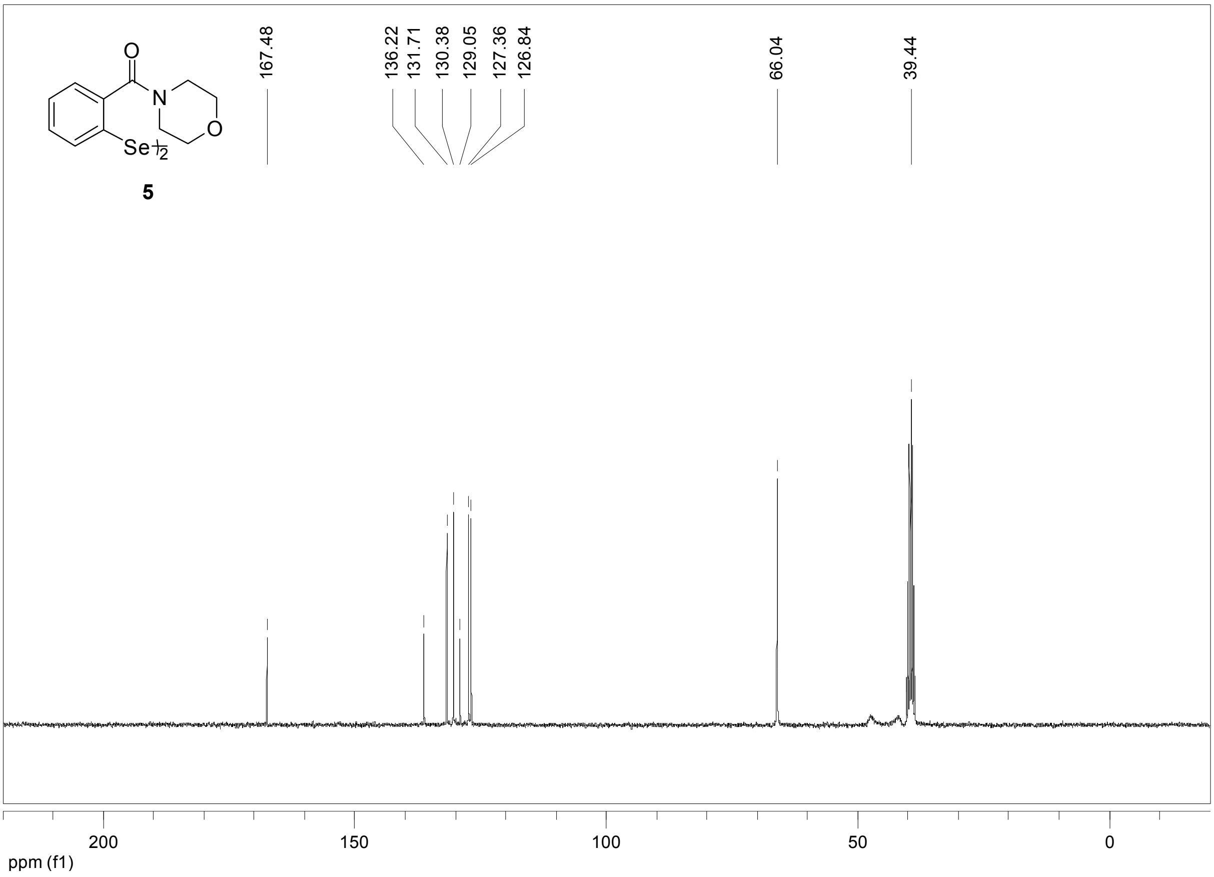

Figure S21. ^{13}C-NMR (DMSO-d$_6$, 75.5 MHz) spectrum of compound **5.**

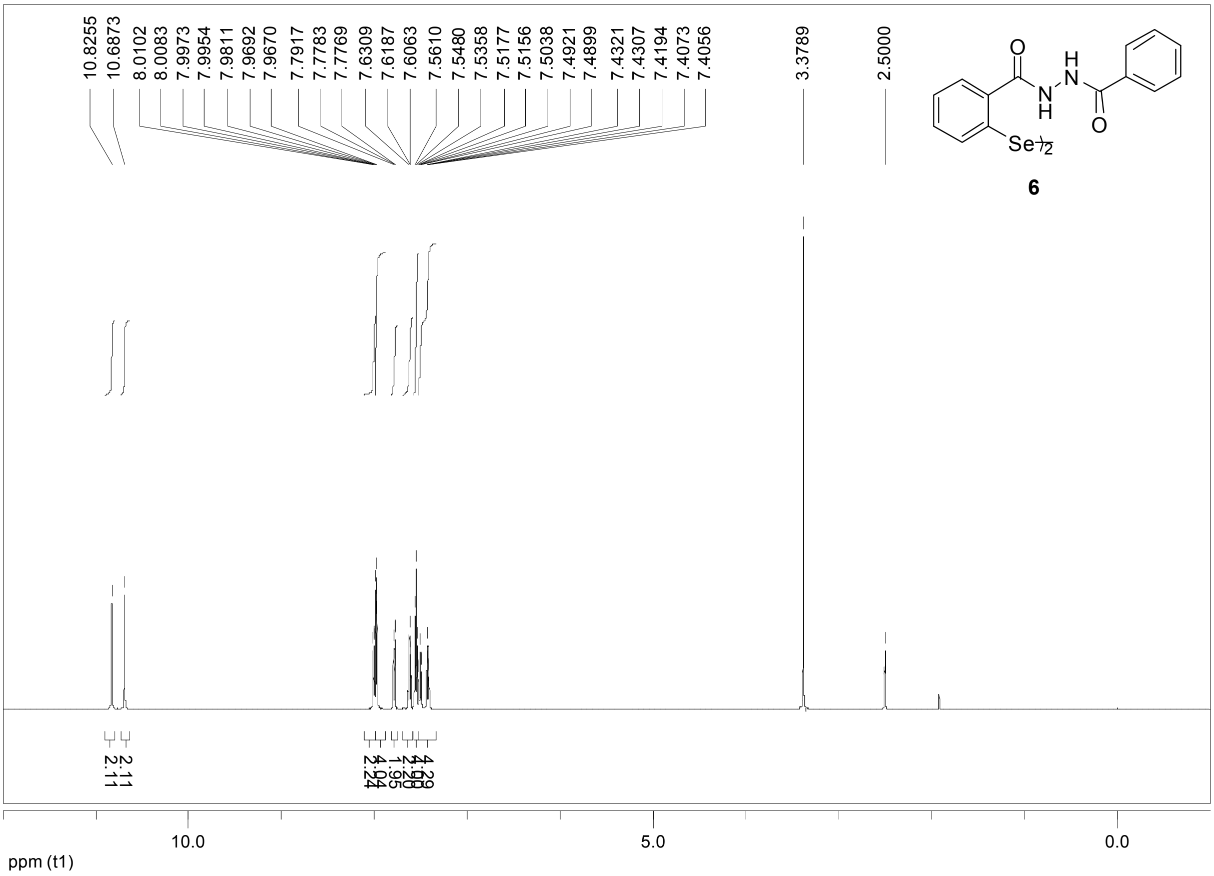

Figure S22. ^{1}H-NMR (DMSO-d$_6$, 600.6 MHz) spectrum of compound **6.**

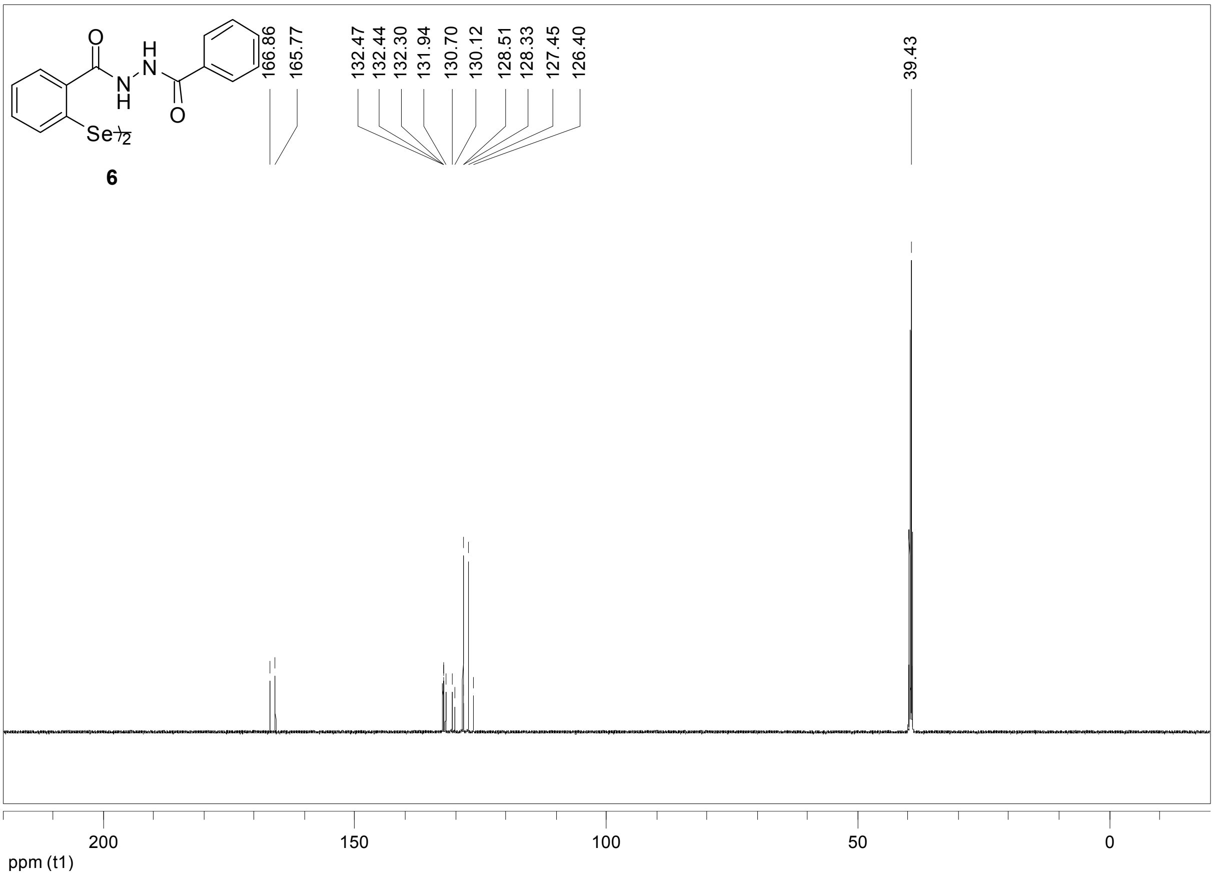

Figure S23. ^{13}C-NMR (DMSO-d$_6$, 151.0 MHz) spectrum of compound **6.**

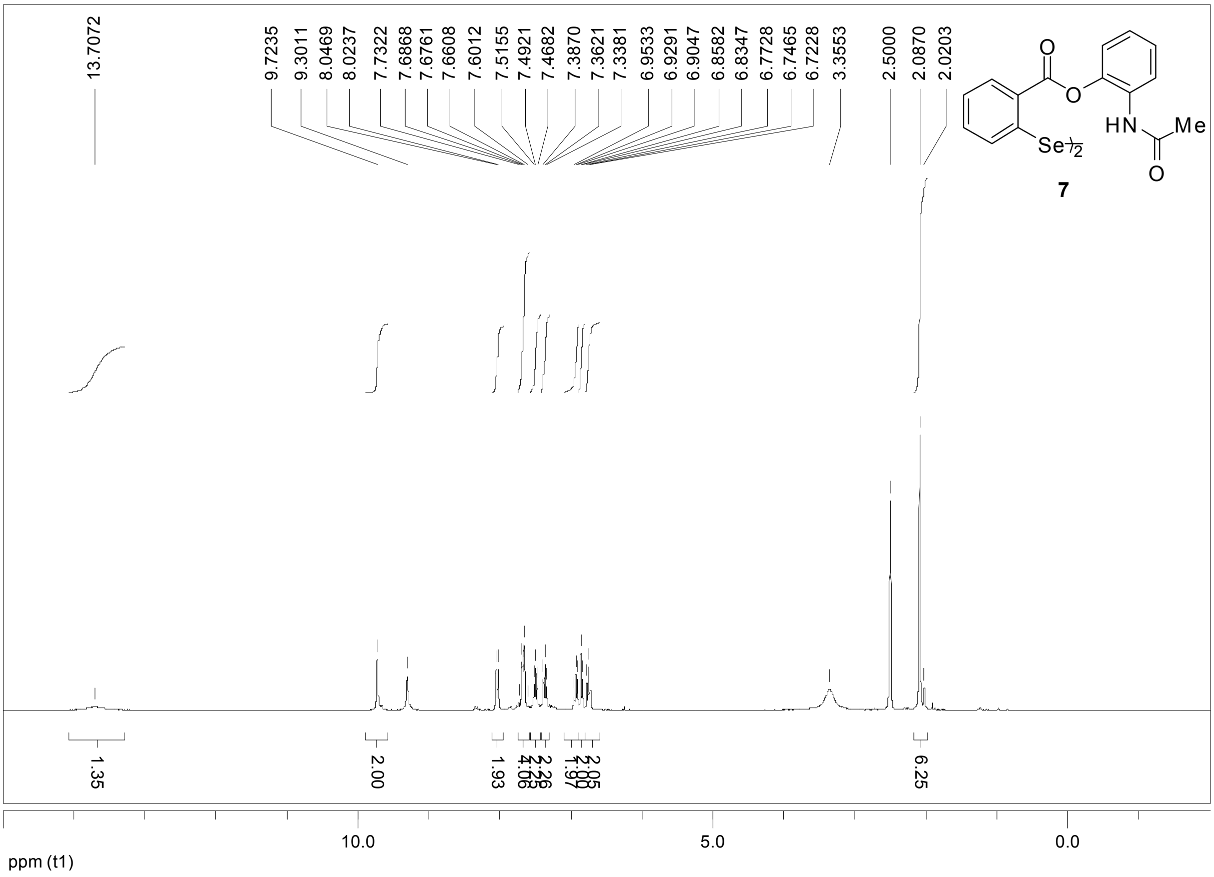

Figure S24. ^{1}H-NMR (DMSO-d$_6$, 300.1 MHz) spectrum of compound **7.**

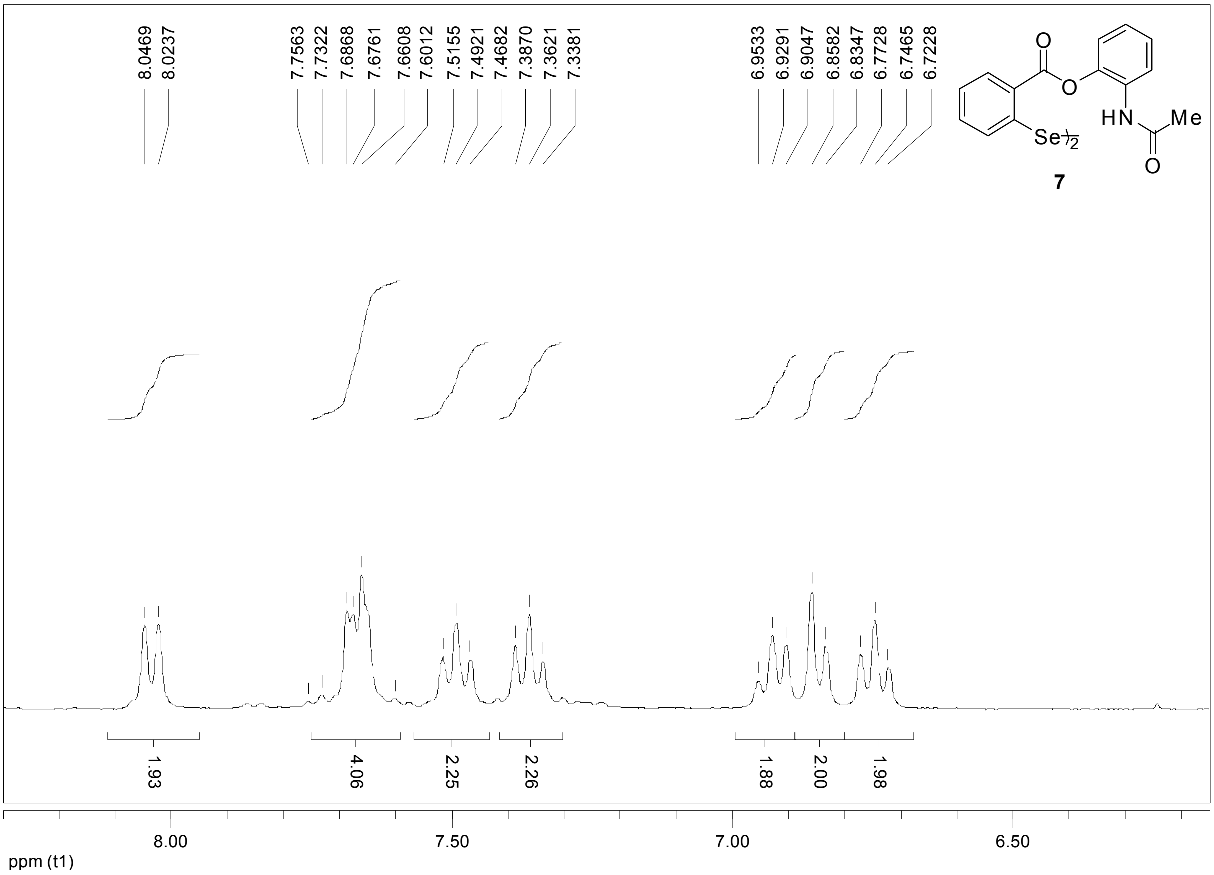

Figure S25. ^{1}H-NMR (DMSO-d_6, 300.1 MHz) expansion spectrum of compound **7.**

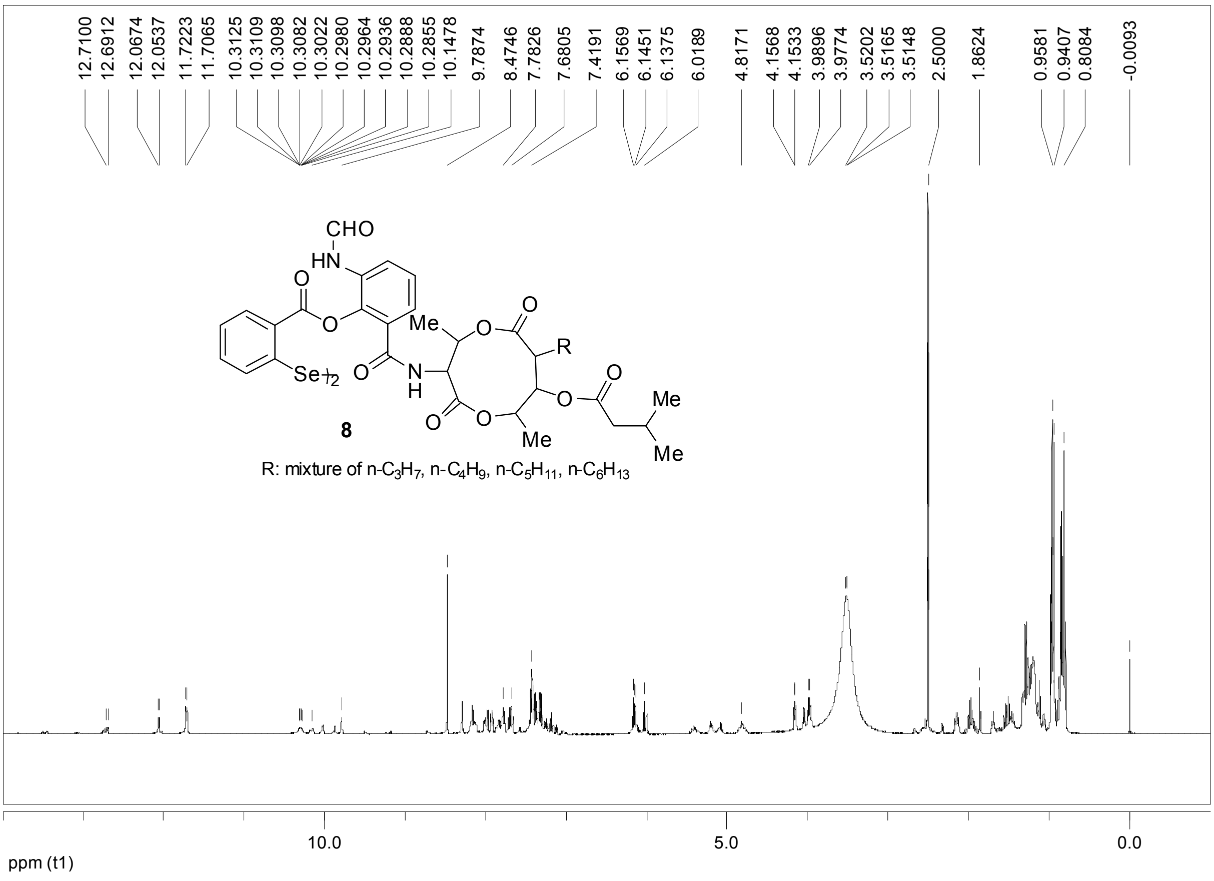

Figure S26. ^{1}H-NMR (DMSO-d_6, 399.8 MHz) spectrum of mixture of compounds **8.**

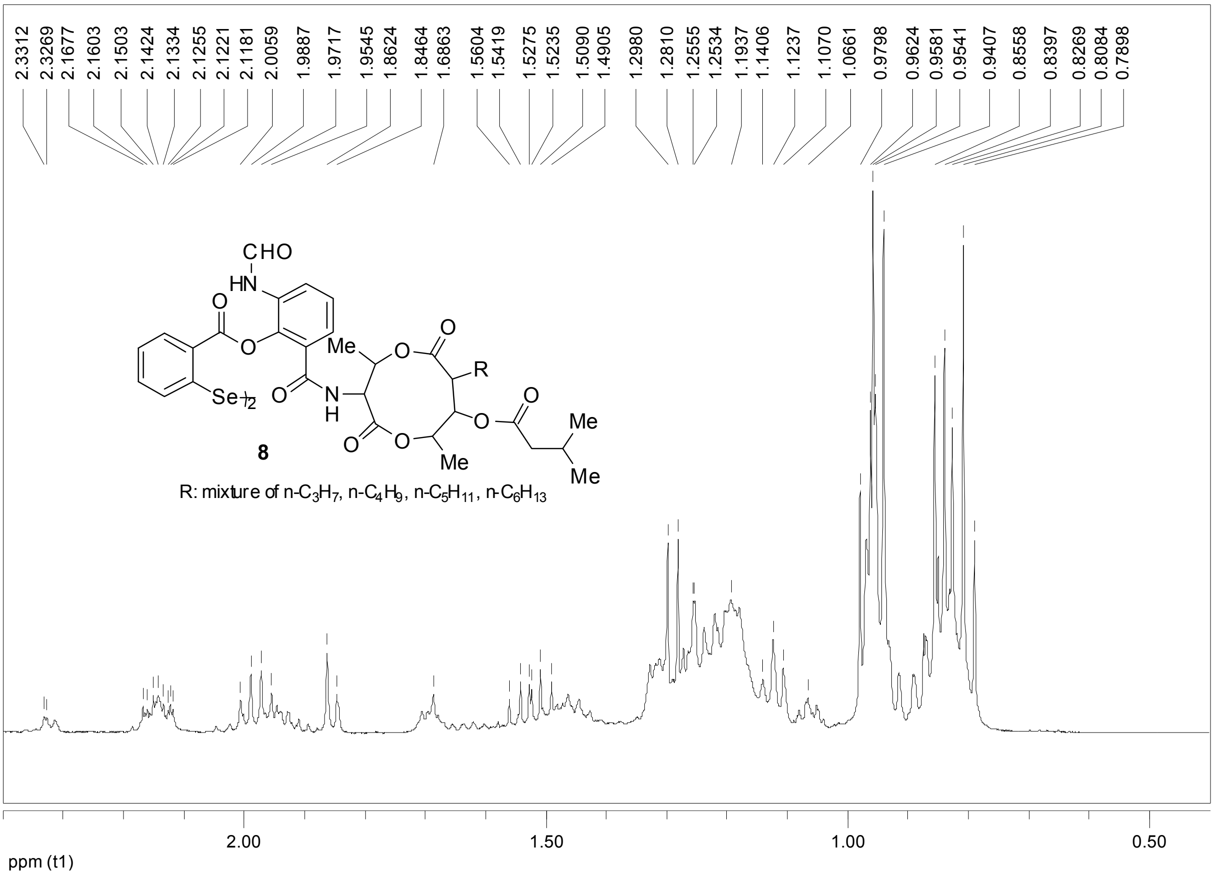

Figure S27. ^{1}H-NMR (DMSO-d_6, 399.8 MHz) expansion spectrum of mixture of compounds **8**.

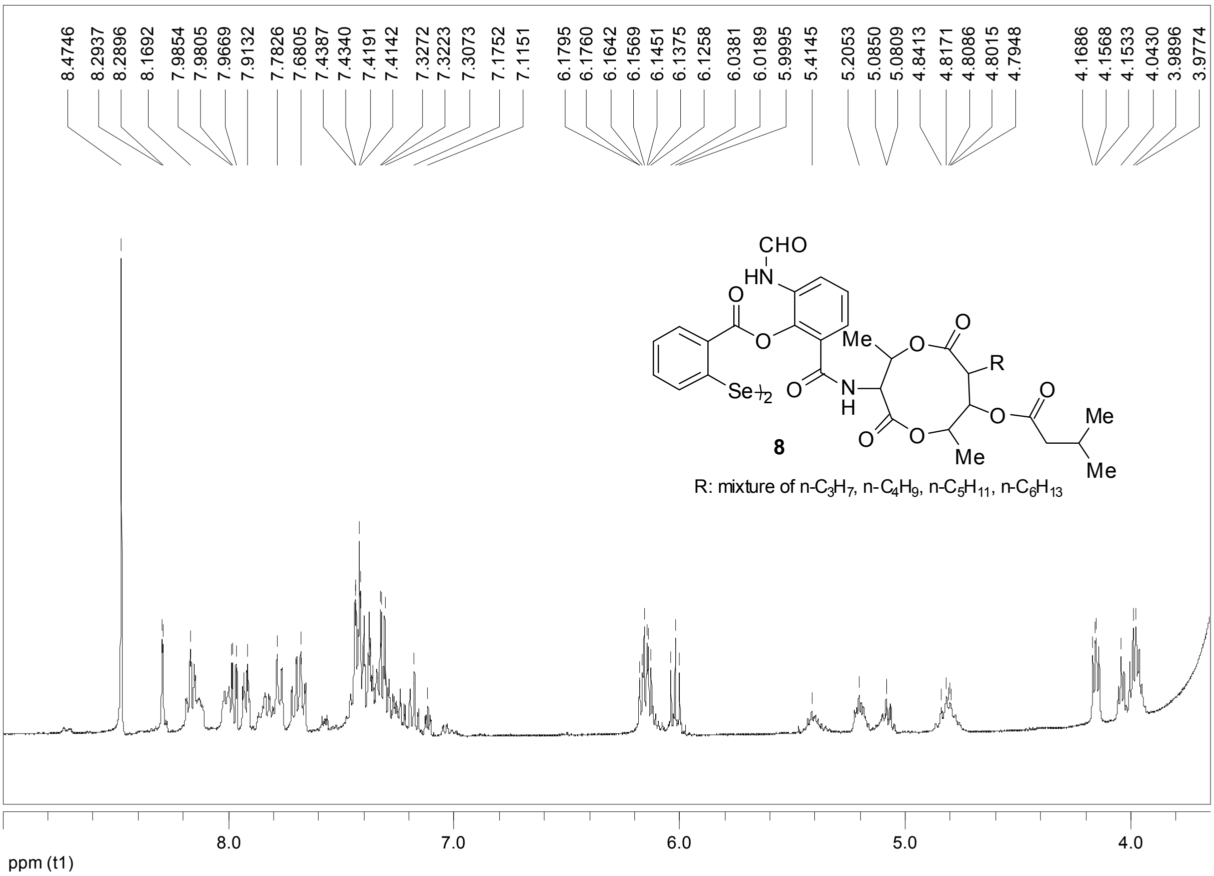

Figure S28. ^{1}H-NMR (DMSO-d_6, 399.8 MHz) expansion spectrum of mixture of compounds **8**.

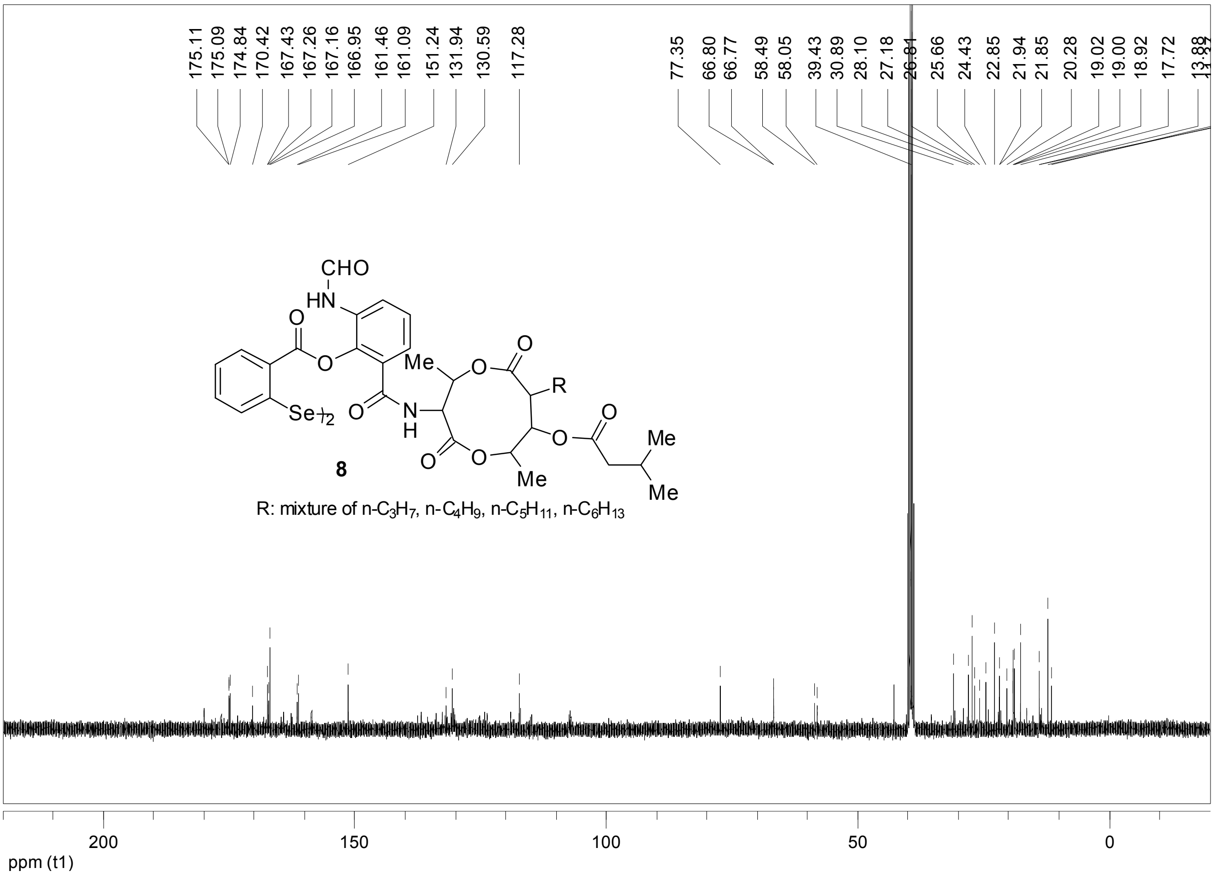

Figure S29. ^{13}C-NMR (DMSO-d$_6$, 151.0 MHz) spectrum of mixture of compounds **8**.

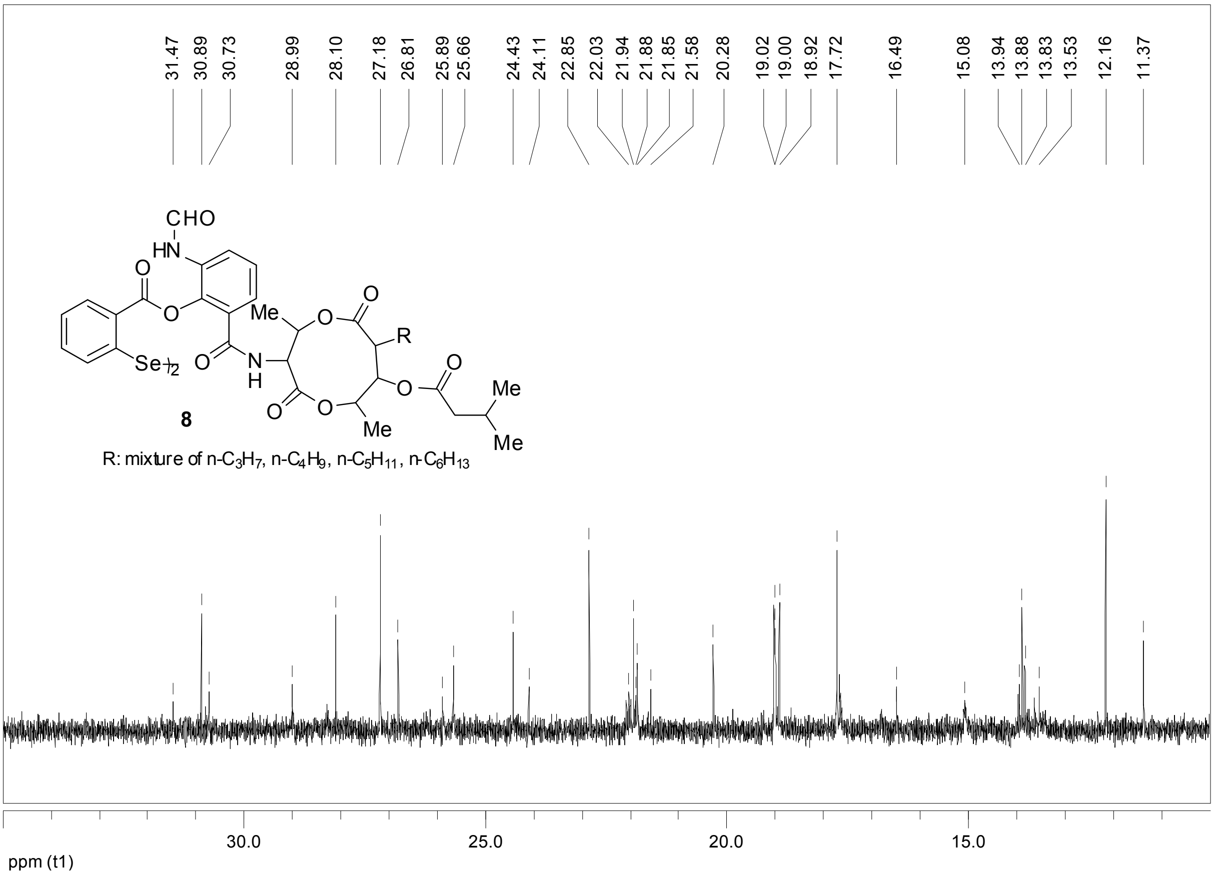

Figure S30. ^{13}C-NMR (DMSO-d$_6$, 151.0 MHz) expansion spectrum of mixture of compounds **8**.

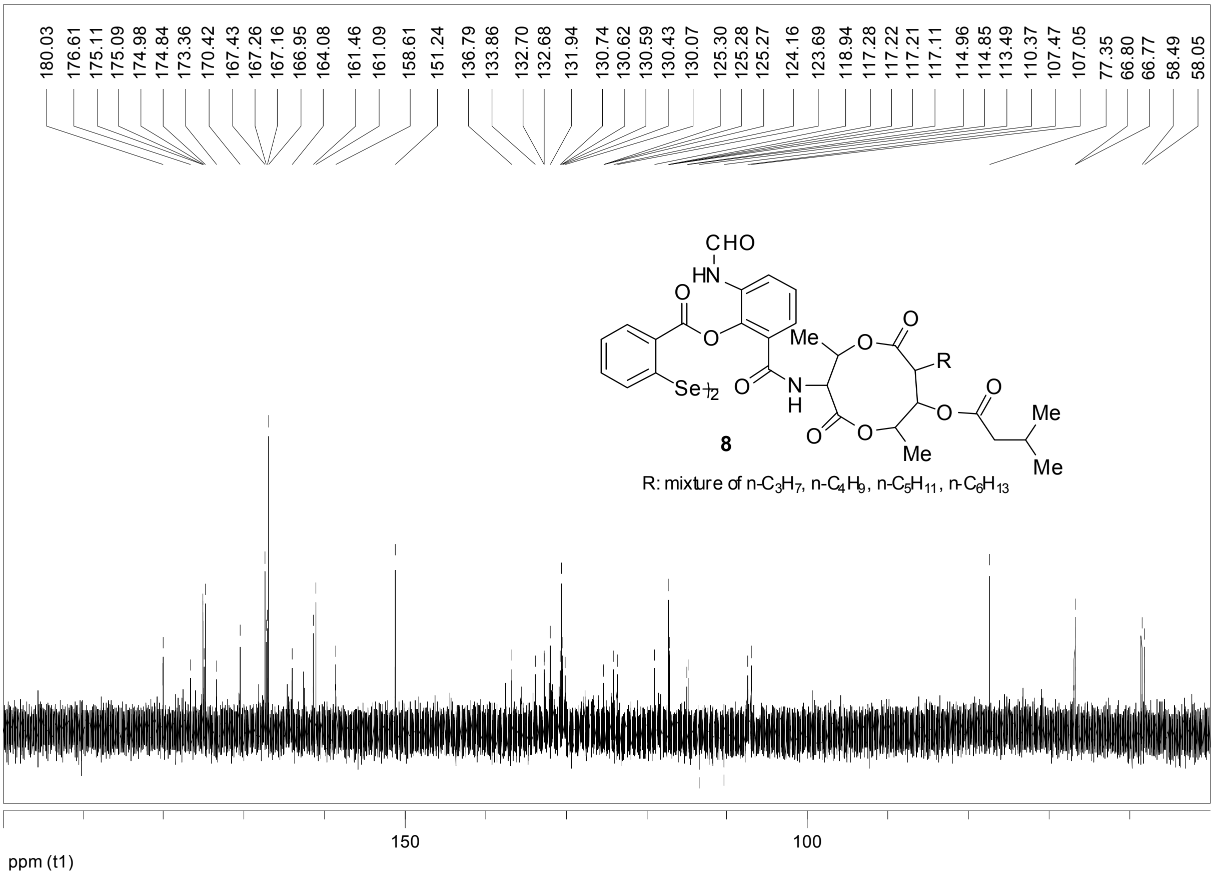

Figure S31. ^{13}C-NMR (DMSO-d_6, 151.0 MHz) expansion spectrum of mixture of compounds **8**.

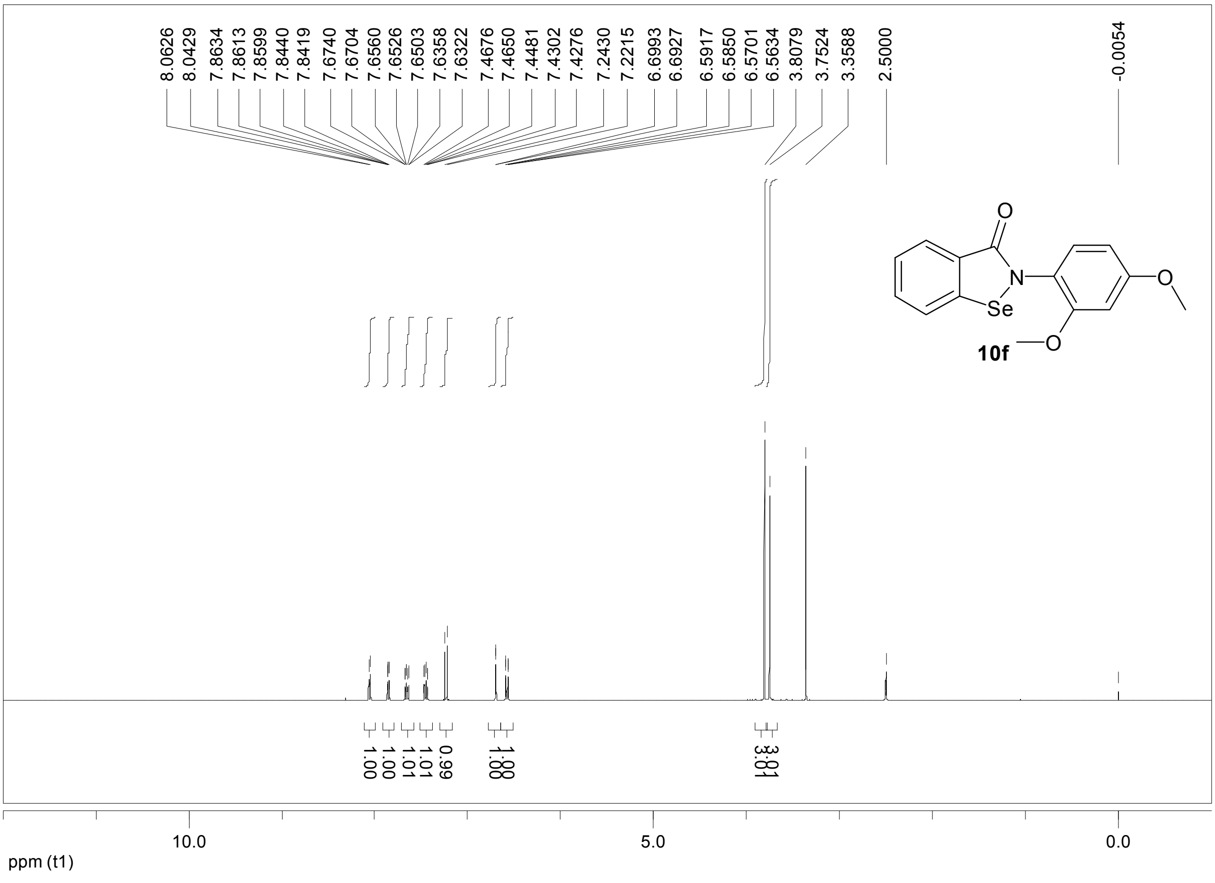

Figure S32. ^{1}H-NMR (DMSO-d_6, 399.8 MHz) spectrum of compound **10f.**

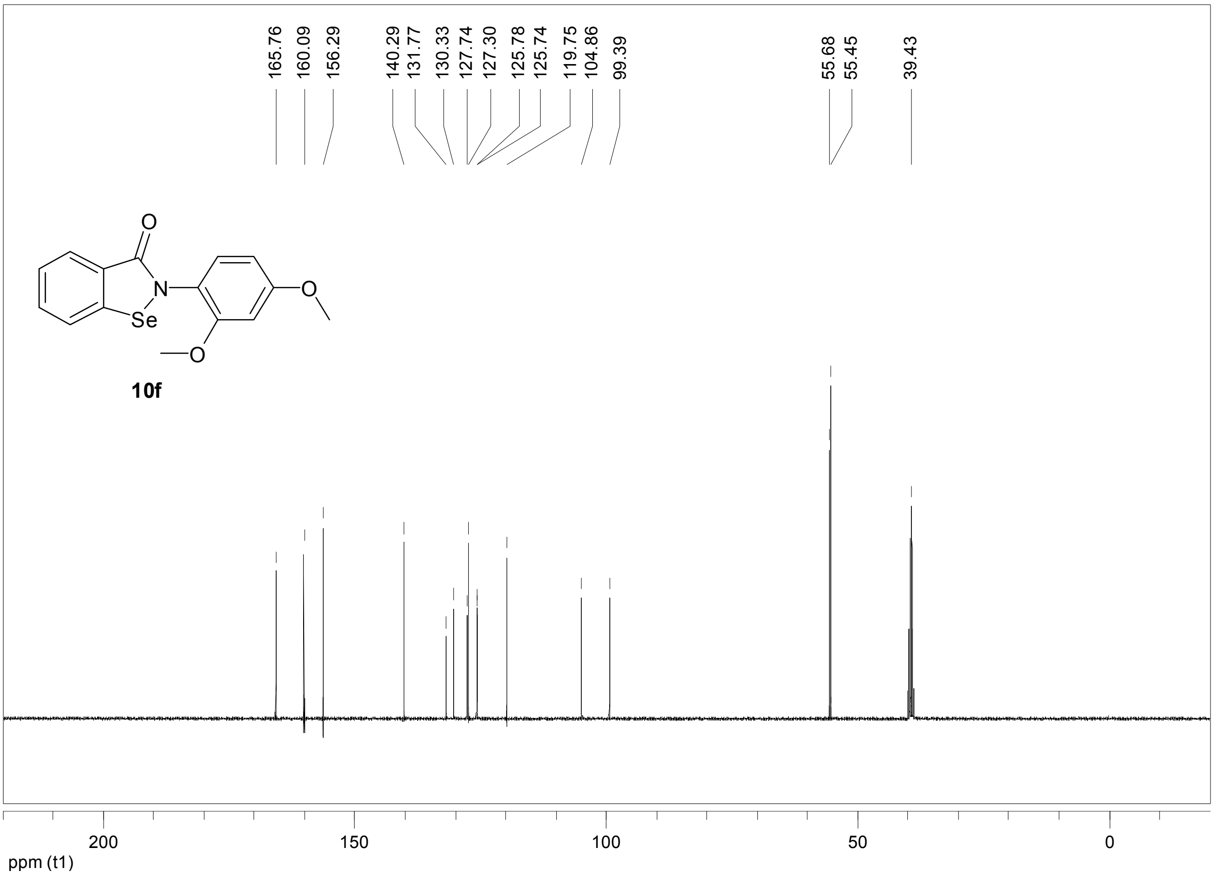

Figure S33. ^{13}C-NMR (DMSO-d$_6$, 100.5 MHz) spectrum of compound **10f.**

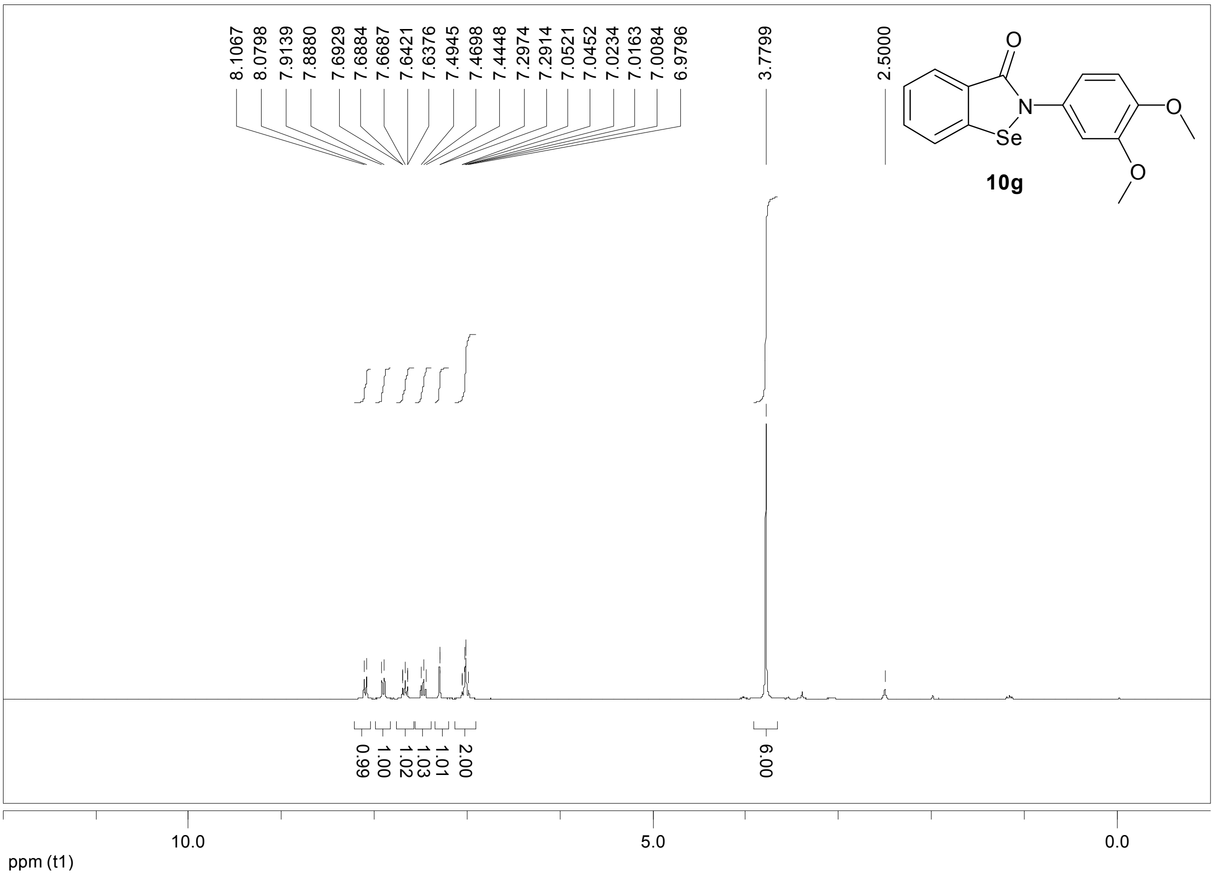

Figure S34. ^{1}H-NMR (DMSO-d$_6$, 300.1 MHz) spectrum of compound **10g.**

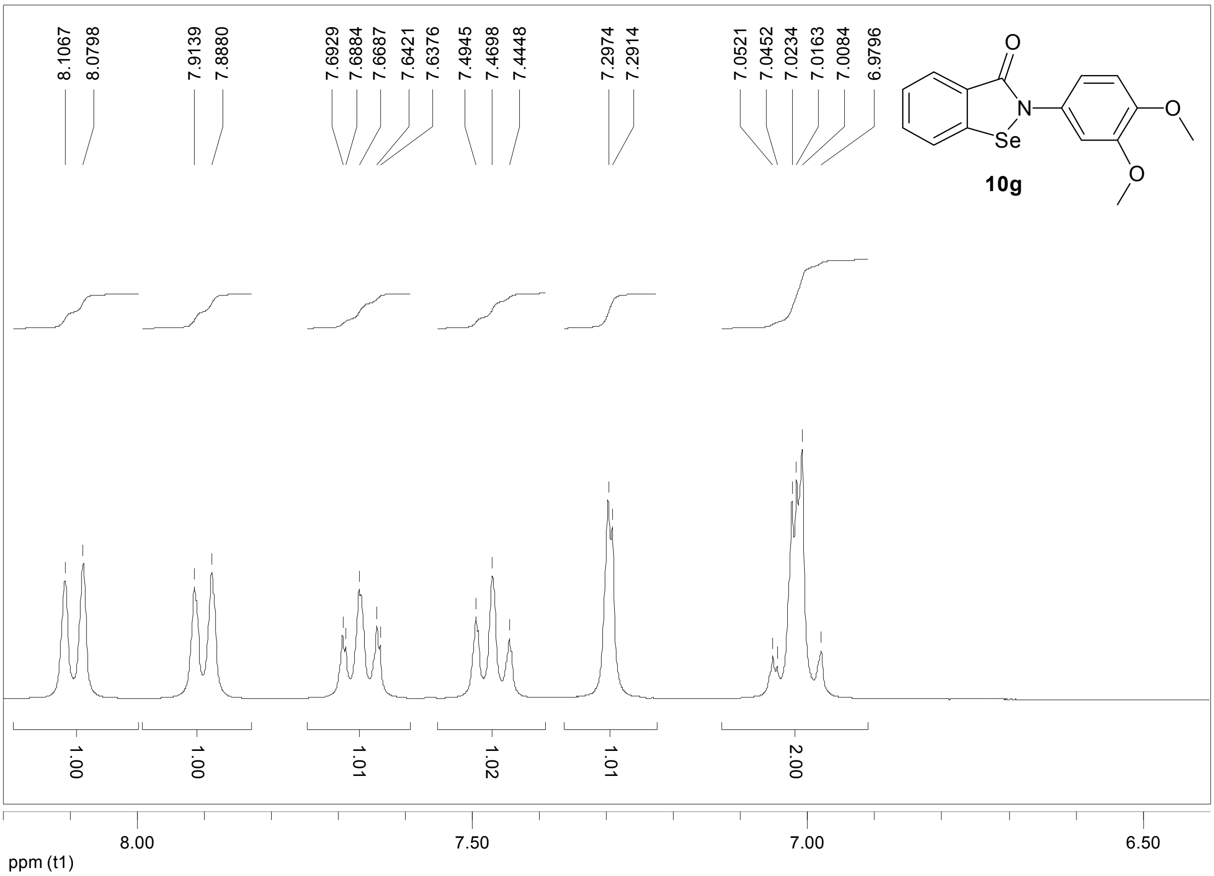

Figure S35. ^{1}H-NMR (DMSO-d_6, 300.1 MHz) expansion spectrum of compound **10g.**

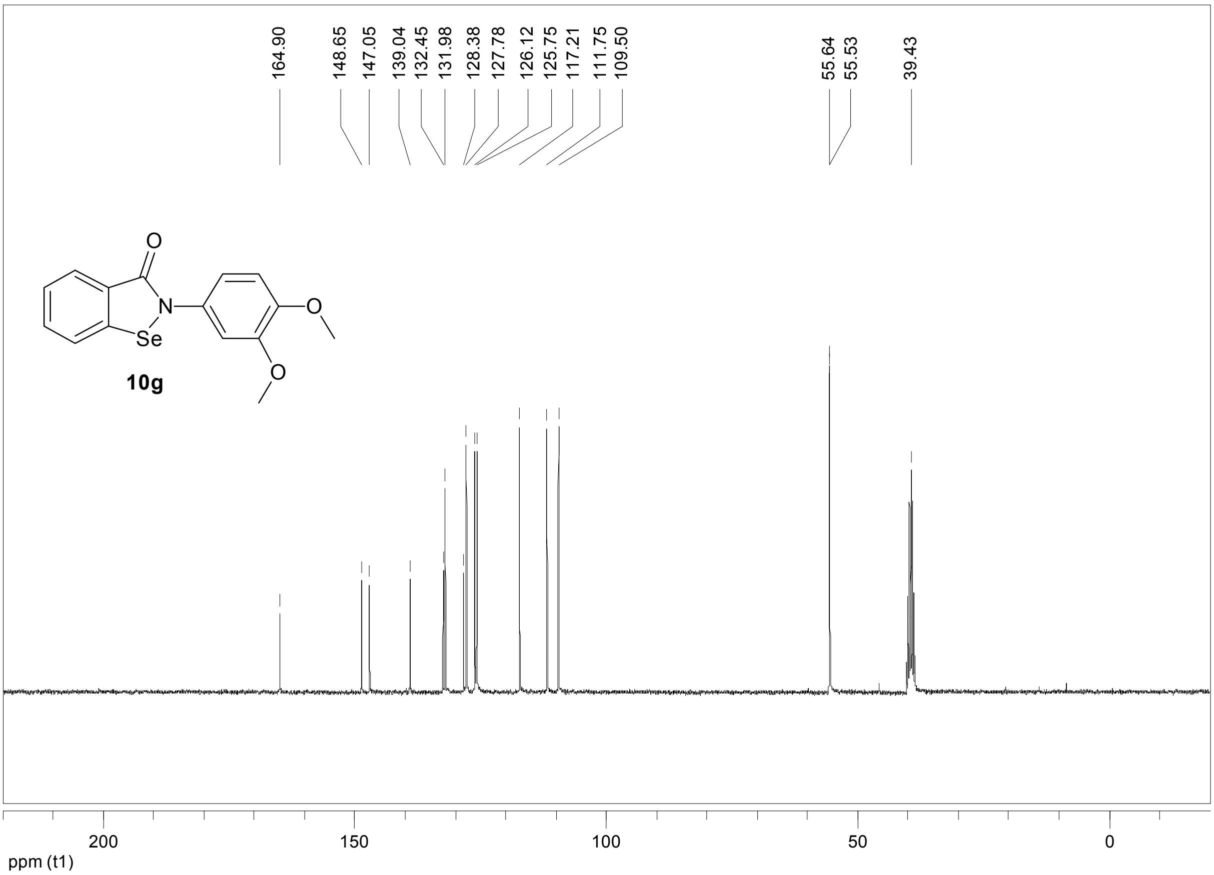

Figure S36. ^{13}C-NMR (DMSO-d_6, 75.5 MHz) spectrum of compound **10g.**
